# Exploring the efficacy of psychotherapies for depression: a multiverse meta-analysis

**DOI:** 10.1136/bmjment-2022-300626

**Published:** 2023-03-13

**Authors:** Constantin Yves Plessen, Eirini Karyotaki, Clara Miguel, Marketa Ciharova, Pim Cuijpers

**Affiliations:** 1 Department of Psychosomatic Medicine, Charite University Hospital Berlin, Berlin, Germany; 2 Department of Clinical, Neuro-, and Developmental Psychology, Vrije Universiteit Amsterdam Faculty of Behavioural and Movement Sciences, Amsterdam, The Netherlands; 3 Amsterdam Public Health Research Institute, Vrije Universiteit Amsterdam, Amsterdam, The Netherlands; 4 WHO Collaborating Center for Research and Dissemination of Psychological Interventions, Vrije Universiteit Amsterdam, Amsterdam, The Netherlands

**Keywords:** Depression & mood disorders

## Abstract

**Background:**

Hundreds of randomised controlled trials and dozens of meta-analyses have examined psychotherapies for depression—yet not all points in the same direction. Are these discrepancies a result of specific meta-analytical decisions or do most analytical strategies reaching the same conclusion?

**Objective:**

We aim to solve these discrepancies by conducting a multiverse meta-analysis containing all possible meta-analyses, using all statistical methods.

**Study selection and analysis:**

We searched four bibliographical databases (PubMed, EMBASE, PsycINFO and Cochrane Register of Controlled Trials), including studies published until 1 January 2022. We included all randomised controlled trials comparing psychotherapies with control conditions without restricting the type of psychotherapy, target group, intervention format, control condition and diagnosis. We defined all possible meta-analyses emerging from combinations of these inclusion criteria and estimated the resulting pooled effect sizes with fixed-effect, random-effects, 3-level, robust variance estimation, *p*-uniform and PET-PEESE (precision-effect test and precision-effect estimate with SE) meta-analysis models. This study was preregistered (https://doi.org/10.1136/bmjopen-2021-050197).

**Findings:**

A total of 21 563 records were screened, and 3584 full texts were retrieved; 415 studies met our inclusion criteria containing 1206 effect sizes and 71 454 participants. Based on all possible combinations between inclusion criteria and meta-analytical methods, we calculated 4281 meta-analyses. The average summary effect size for these meta-analyses was Hedges’ *g*_mean_=0.56, a medium effect size, and ranged from *g*=−0.66 to 2.51. In total, 90% of these meta-analyses reached a clinically relevant magnitude.

**Conclusions and Clinical Implications:**

The multiverse meta-analysis revealed the overall robustness of the effectiveness of psychotherapies for depression. Notably, meta-analyses that included studies with a high risk of bias, compared the intervention with wait-list control groups, and not correcting for publication bias produced larger effect sizes.

**WHAT IS ALREADY KNOWN ON THIS TOPIC**

- To this day, more than 80 meta-analyses have examined the efficacy of psychotherapies for depression. However, recent meta-research projects questioned whether the effect sizes from those published meta-analyses were inflated and discrepancies between these meta-analyses emerged.

**WHAT THIS STUDY ADDS**

- In a so-called multiverse meta-analysis, we calculated over 4000 meta-analyses—most of them (90%) produced small but clinically relevant effect sizes. Our findings suggest that psychotherapies for depression are generally effective, but the specific type, format and other factors can affect the magnitude of the treatment effect slightly. However, meta-analyses that (1) restrict their control group to wait-list control groups, (2) do not exclude high risk of bias studies and (3) do not correct for publication bias are likely to produce larger effect sizes.

**HOW THIS STUDY MIGHT AFFECT RESEARCH, PRACTICE OR POLICY**

- The effect size reported in any given meta-analysis on treatment efficacy for depression depends less on the type of psychotherapy, treatment format, diagnosis or target group but rather on the comparison with wait-list control groups, not excluding the high risk of bias studies, or correcting for publication bias. In general, future meta-analyses that diverge from small-to-medium summary effect size estimates may be indicative of extreme data analytical decisions and may therefore not contribute additional substantive knowledge. However, there are certain circumstances where higher treatment effects might be observed, such as when a future meta-analysis examines a specific subgroup of patients or a new type of therapy not previously studied.

## Background

Over the last four decades, more than 80 meta-analyses have examined the efficacy of psychotherapies for depression.^1^ In these meta-analyses, evidence from more than 700 randomised controlled trials (RCTs) is included, yet not all of these studies are pointing in the same direction.^2 3^ Contested evidence exists on efficacy claims between different psychotherapies for depression (eg, therapies based on cognitive–behavioural therapy (CBT) or other types of psychotherapy),^2–12^ target groups (eg, adults or general medical populations) and delivery formats (eg, individual or group therapy).

Some of the discrepancies in findings may be the result of publication bias leading to an overestimation of the effectiveness of psychotherapy or may be due to variations in inclusion criteria, such as the inclusion of low-quality studies or studies comparing interventions with wait-list control groups only.^13–18^ It is crucial to evaluate the influence such meta-analytical decisions have. For example, does it make a substantial difference when we correct for publication bias or not? Does the evidence depend on whether we include only the best evidence or all evidence? Are the results robust to slightly different inclusion criteria? This exploration is especially important when multiple meta-analyses with overlapping research questions reach different conclusions.^19 20^ To increase trust in the existing evidence, we need to ensure that the published results do not depend on these specific decisions in selecting and analysing the data but rather that most analytical strategies reach the same conclusion.

Although conventional meta-analyses exist on some of these specific aspects, a comprehensive bird’s-eye view of all meta-analyses for depression treatment research is missing. Ideally, this birds-eye view does not only include all published meta-analyses but also all possible meta-analyses based on defensible and reasonable analytical choices. To provide such an overview and fill substantive knowledge gaps, we conducted a so-called multiverse meta-analysis and calculated all possible meta-analyses on the efficacy of psychotherapies for depression in a single analysis. It can (1) integrate multiple meta-analyses like an umbrella review, (2) enable us to identify knowledge gaps and (3) investigate how flexibility in data selection and analysis might affect the overall interpretation of results. In doing so, this new approach can help solve diverging claims on the efficacy of psychotherapies once and for all. We replicated most meta-analyses that have ever been conducted in research on psychotherapy for depression and created additional evidence by conducting thousands of meta-analyses that were still missing in the literature.

Due to the sheer number of published meta-analyses and primary studies on these differences between psychotherapies, we aimed to summarise, integrate and visualise the entire evidence. As a result, we can highlight robustness—or lack thereof—by inspecting all possible meta-analyses to help resolve conflicting meta-analyses and contested evidence, alleviate the associated adverse effects of these phenomena on research progress and provide a birds-eye perspective of the entire field.^21^

## Study selection and analysis

### Search strategy and selection criteria

We searched four major bibliographic sources (PubMed, PsycINFO, EMBASE and Cochrane Library; see online supplemental eMethods 1 for all search strings) for RCTs of psychotherapies for depression published until 1 January 2022.^1 22^ After title and abstract screening, two independent researchers conducted full-text screening of all records. We included all RCTs comparing a psychological intervention with any control condition written in English, German, Spanish or Dutch. We excluded maintenance and relapse prevention trials, dissertations and interventions not aimed at depression. Eligible were both self-reported and clinician-rated instruments measuring depression. Therapies could be delivered by any person trained to deliver the therapy. Two independent researchers extracted information on target groups, intervention formats, psychotherapy types, control conditions and countries. Inconsistencies were resolved by discussion. This study was preregistered (https://doi.org/10.1136/bmjopen-2021-050197).

10.1136/bmjment-2022-300626.supp1 Supplementary data

### Data analysis

Our study protocol outlines the analyses in more detail (see online supplemental eMethods 3 for deviations from our protocol).^23^ The R code and data to reproduce all analyses can be found at the Open Science Framework (https://osf.io/mtx8a/).^24^ All analyses were carried out using R (V.4.1.2)^25^ and the metafor package (V3.4.0).^26^ We calculated standardised mean differences (Hedges’ *g*) for postintervention comparisons between psychotherapy and control conditions based on continuous outcome data provided in the primary studies. If only change score or dichotomous outcome data were reported, we converted these data into Hedges’ *g* with the metapsyTools R package.^27^ We assessed the risk of bias of included studies using four criteria of the risk of bias assessment tool, developed by the Cochrane Collaboration^28^: adequate generation of allocation sequence, concealment of allocation to conditions, masking of assessors and dealing with incomplete outcome data (this criterion was met when intention-to-treat analyses were conducted). All items were rated as positive (the criterion was met) or negative (the criterion was not met or unclear). The total risk of bias score for each study was calculated as the sum of all positive scores (ranging from 0 to 4, with 4 indicating no risk of bias). We rated a study having overall ‘some concern’ for risk of bias when the study had a rating of 1 or higher. Two researchers conducted the risk of bias independently, and disagreements were resolved through discussion.

#### Multiverse meta-analysis

Our multiverse meta-analysis contains every single meta-analysis based on a defensible combination of subgroups (eg, target group of the intervention, type and format of intervention) and statistical methods investigating psychotherapies targeting depression that could possibly be conducted.

##### Descriptive specification curve

We specified seven the so-called *Which* factors—asking which data to meta-analyse—and one *How* factors—asking how to meta-analyse the data. Based on these *Which* factors, meta-analyses could include studies investigating different *target groups* that received different *types of psychotherapies* in different *formats* and *assessments/diagnoses* of depression. These studies could have different *risks of biases’* ratings and could be compared with different *control groups* (see online supplemental eMethods 2 for a detailed description of all *Which* factors). Based on our *How* factor, we used six different meta-analytical methods to analyse the data: random-effects, fixed-effect, 3-level, robust variance estimation (RVE),^29^ precision-effect test and precision-effect estimate with SEs (PET-PEESE)^30^ and *p*-uniform* meta-analysis models.^31^

Some primary studies reported multiple effect sizes per study, that is, when multiple instruments were used to measure depression or when a study consisted of multiple interventions or control groups. These nested effect sizes are not independent as they are correlated (we assumed a correlation of *r*=0.5) and introduce a unit-of-analysis problem.^32 33^ Such effect size dependencies were handled by either averaging the effect sizes included in each study or modelling the dependency directly. For this hierarchical modelling of dependencies among effect sizes within studies, we included 3-level and RVE models. RVE methods allow for the inclusion of all relevant effect sizes in a single meta-regression model, regardless of the specific nature of the dependencies between them.

Additionally, we used two novel methods for addressing ‘small-study effects’, which can arise due to publication bias (eg, when statistically non-significant findings are not published), reporting bias (eg, when statistically significant results are selectively reported) or clinical heterogeneity (eg, when smaller studies include more severely ill patients than larger studies). This phenomenon, which is common in many scientific fields, can cause inflated effect sizes and therefore an overestimation of treatment effectiveness.^34^

PET-PEESE is a regression-based method for addressing the issue of *small-study effects* in meta-analyses.^30 34^ It is based on the relationship between effect sizes and SEs and is part of a broader class of funnel plot–based methods, such as the trim-and-fill method or Egger’s regression test.^35 36^ In the absence of publication bias and reporting bias, the relationship between effect sizes and SEs should be unrelated. However, publication bias often results in a disproportionate number of larger studies being published, while smaller studies are only published if they show statistically significant results. This can lead to an over-representation of imprecise studies with inflated effect size estimates in the published literature.

PET-PEESE aims to correct for this bias and provides more accurate estimates of effect sizes.

*P*-uniform* is a selection model approach that uses a random-effects model as its effect size model.^31^ This method assumes that the probability of publishing a statistically significant or non-significant effect size is constant, but these probabilities may be different from each other. *P*-uniform* works by treating the primary studies’ effect sizes differently based on whether they are statistically significant or not. This method can be considered a selection model approach with a single cut-off value that determines whether an effect size is considered statistically significant.

The results of the multiverse meta-analysis were depicted in a descriptive specification curve plot, which is a graph that shows the results of all conducted meta-analyses, represented by points on the graph with 95% CIs. The summary effects are plotted in order of magnitude, from lower to higher, and connected by a solid line—the specification curve. To evaluate the number of meta-analyses that exceed relevant magnitudes, the plot focuses on two thresholds: meta-analyses that do not include 0 in their 95% CI (ie, meta-analyses that exceed a null effect) and meta-analyses that exceed a clinically relevant effect size of Hedges’ *g*=0.24, which was suggested as the minimal important difference for interventions targeting major depressive disorder.^37^ The plot includes vertical columns that represent different combinations of factors that may influence the meta-analyses, such as target group, therapy type and control group.

##### Inferential specification curve

In a second step, we evaluated if the findings of the descriptive specification curve (the magnitude sorted summary effect sizes from all meta-analyses of the multiverse) are likely to be true or if they could be due to chance. This so-called inferential specification curve analysis involves simulating new random-effect sizes for each primary study under the assumption that the null hypothesis, or the assumption that there is no psychological treatment effect on depression, is true.

These new data sets are created by drawing random values for the effect sizes from a normal distribution with mean zero and an SD that takes into account both the variance of the original effect size estimate for each study and a measure of between-study heterogeneity (τ values obtained from fitting a random-effects model with REML estimator and handling effect size dependency by averaging studies with multiple effect sizes on the entire data set).

We then applied a new descriptive specification curve analysis under three scenarios: a fixed-effect scenario with no heterogeneity, a scenario with heterogeneity equal to the random-effects model of the 415 studies and a scenario with the upper 95% CI estimate of τ from the random-effects model. By repeating this process 1000 times, we were able to identify the lower and upper limits of the resulting 1000 bootstrapped specification curves or their 2.5% and 97.5% quantiles. These limits, or quantiles, represent the range within which we would expect 95% of all meta-analyses to fall if the true underlying effect were a null effect. If the observed treatment effect falls outside of these limits, it is considered to be a deviation from the null hypothesis and is considered to be a likely true effect.

Additionally, we conducted a GOSH plot (Graphical Display of Study Heterogeneity) to identify the overall range of possible summary effect sizes for meta-analyses and visualise the heterogeneity of effect sizes.^38^ This plot is a visual tool that can be thought of as a brute force sensitivity check because it calculates all possible meta-analyses from all possible subsets of included studies. It is not restricted to the more theoretically guided comparisons defined by the descriptive specification curve analysis. The GOSH plot shows the relationship between the effect size of each meta-analysis and its heterogeneity, which is a measure of the variability of effect sizes across studies. We used a reduced set of 100 000 randomly drawn samples as this sensitivity check is computationally infeasible with 415 primary studies, as 2^415^=8.46×10^124^.

##### Conventional meta-analysis

To create a reference point for exploring heterogeneity in the data, we additionally fitted a 3-level model to the entire data set. This resulting summary effect size represented *one* possible meta-analysis—including the broadest inclusion of all *Which* factors and the 3-level *How* factor—out of the entire multiverse of meta-analyses. The amount of heterogeneity was estimated using the restricted maximum likelihood estimator. In addition to the estimate of $\tau^{2}$ , both the Q-test for heterogeneity and the $I^{2}$ statistic were reported.

## Findings

**Figure 1:**
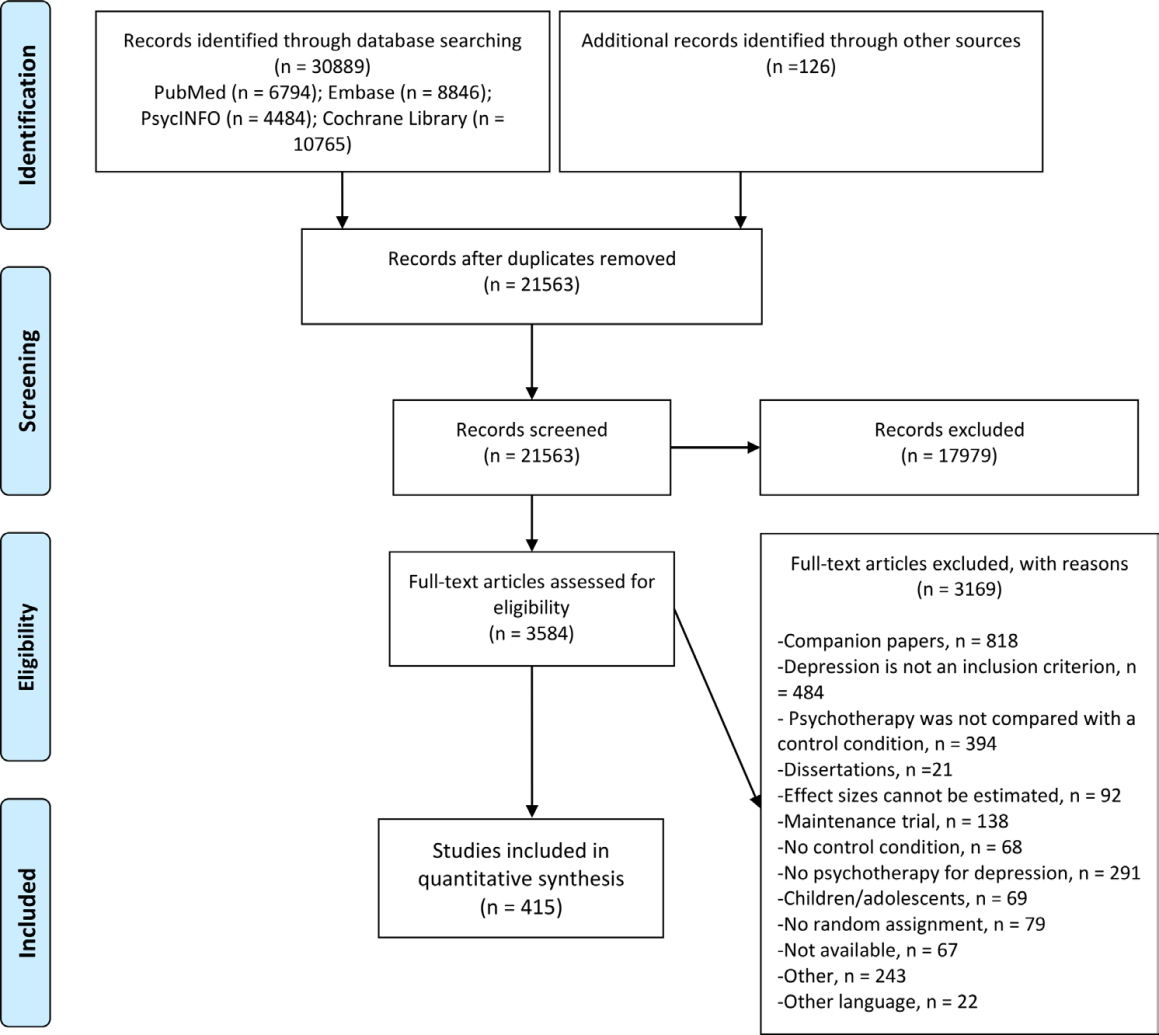
PRISMA (Preferred Reporting Items for Systematic Reviews and Meta-Analyses) flow chart for inclusion of studies.

We screened 21 563 titles and examined 3584 full-text papers, of which 415 were included. The PRISMA (Preferred Reporting Items for Systematic Reviews and Meta-Analyses) flow chart depicts the study inclusion process in figure 1. Overall, *k_es_* =1206 effect sizes from *k_studies_* =415 studies were included. The sample sizes of the included primary studies ranged from n=4 to 1156, *N_mean_* =103, *N_median_* =67. The total sample size of all included samples from all primary studies was *N_total_* =71 454. See online supplemental eTable 1 for a detailed description of all included primary studies.

Most included primary studies investigated the efficacy of psychotherapies for depression in adults (37%) or general medical populations (23%). Most studies were either conducted in Europe (37%) or in Northern America (36%). Most studies used CBT-based interventions (69%) and were primarily delivered in individual therapy format (35%). Care-as-usual was the most common type of control condition (49%), and depressive disorder was diagnosed by a clinician in 49% of studies. Only 35% of studies were rated with a low risk of bias. See table 1 for the study characteristics and online supplemental eTable 2 for the effect size characteristics.

**Table 1:** Summary characteristics of included primary studies

Characteristic	*k_studies_* =415
Target group	
Adults	155 (37%)
General medical	94 (23%)
Older adults	38 (9.2%)
Other target groups	50 (12%)
Perinatal depression	58 (14%)
Student population	20 (4.8%)
Region	
Australia	24 (5.8%)
East Asia	42 (10%)
Europe	152 (37%)
North America	148 (36%)
Other region	49 (12%)
Intervention	
CBT based	285 (69%)
Non-CBT based	130 (31%)
Format	
Group	128 (31%)
Guided self-help	80 (19%)
Individual	147 (35%)
Other formats	60 (14%)
Control	
CAU	203 (49%)
Other control	66 (16%)
Wait-list	146 (35%)
Diagnosis	
Cut-off score	187 (45%)
Diagnosis	203 (49%)
Subclinical depression	25 (6.0%)
Risk of bias	
High	4 (1.0%)
Low	146 (35%)
Some concern	265 (64%)

CAU, care-as-usual; CBT, cognitive–behavioural therapy.

Our multiverse meta-analysis produced 4281 unique meta-analyses, with effect sizes ranging from Hedges’ *g*=−0.66 to 2.51. Half of those effect sizes were in the IQR of Hedges’ *g*=0.42 to 0.71, representing small-to-medium effect sizes. In total, 97% of the effect sizes were greater than 0, and 84% of these had 95% CIs that did not include 0 (ie, estimated *g* was greater than 0 which would have returned a two-tailed p-value of less than 0.05). In total, 90% reached a clinically relevant magnitude of Hedges’ *g* >0.24, and 68% of the summary effect sizes had 95% CIs above the clinically relevant cut-off.

**Figure 2:**
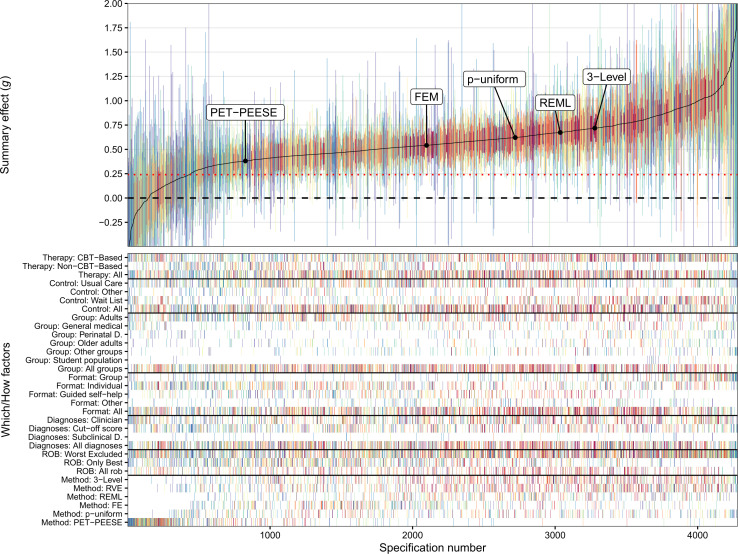
Descriptive specification curve: psychotherapies for depression. The top panel shows the outcome of all 4281 meta-analyses (Hedges’ *g*) with their 95% CIs. The summary effects are sorted by magnitude, from lower to higher. Connecting the different summary effects results in the solid line, which is the specification curve. A horizontal dashed line of no effect is shown at *g*=0, and a red dotted line indicates a clinically relevant effect size at *g*=0.24. The vertical columns in the bottom panel represent factor combinations of *How* factors (different target groups, formats, therapy types, control groups and diagnoses) and *Which* factors (3-LVL, 3-level model; FEM, fixed-effect model; PET-PEESE, *p*-uniform*; REML, random-effects model; RVE, robust variance estimation) constitute a given specification. The location of each *Which* factors’ largest meta-analysis—containing information from all 415 studies—is depicted on the specification curve. Each vertical column is colour-coded, signifying the number of samples included in a specification (hot spectral colours for more included samples vs cool spectral colours for less included samples). CBT, cognitive–behavioural therapy; PET-PEESE, precision-effect test and precision-effect estimate with SE; ROB, risk of bias.

The overall pattern of the descriptive specification curve indicates that larger meta-analyses, including more primary studies, had medium-to-large effect sizes and were close to the median-estimated effect size of the multiverse (see figure 2). More extreme meta-analytical effect sizes were associated with few included studies and therefore broader CIs.

Several *Which* and *How* factors produced—on average—systematically different summary effect size estimates compared with others. In the following, we descriptively summarise the most important results. It is important to note that this examination of these differences is based on descriptive analysis rather than a formal statistical comparison. For a more detailed breakdown of each *Which* factor, see online supplemental eFigures 1–7 and online supplemental eTables 3–9.

### Target group

Meta-analyses including only student populations, mean *g*=0.82, 95% CI (0.51, 1.12) from *k*=54 included meta-analyses, produced larger effect size estimates than meta-analyses on adults, mean *g*=0.51, 95% CI (0.26, 0.75) with *k*=1076.

### Format

Meta-analyses including studies delivered in a group format, mean *g*=0.76, 95% CI (0.32, 1.19) with *k*=536, produced larger effect size estimates than meta-analyses delivered as guided self-help interventions, mean *g*=0.50, 95% CI (0.27, 0.72) with *k*=586.

### Type

Meta-analyses focusing only on CBT-based treatments, mean *g*=0.60, 95% CI (0.29, 0.91) with *k*=1620, produced larger effect size estimates than meta-analyses focusing on non-CBT treatments, mean *g*=0.48, 95% CI (0.19, 0.78) with *k*=733.

### Control group

Meta-analyses that included samples compared with a wait-list control group, mean *g*=0.66, 95% CI (0.35, 0.96) with *k*=836, produced larger effect size estimates than treatments compared with care-as-usual, mean *g*=0.52, 95% CI (0.22, 0.82) with *k*=1194.

### Risk of bias

Meta-analyses that excluded high risk of bias studies, mean *g*=0.61, 95% CI (0.27, 0.95) with *k*=2413 included samples, produced larger effect size estimates than meta-analyses including only low risk of bias studies, mean *g*=0.45, 95% CI (0.19, 0.72) with *k*=1034.

### Diagnosis

Meta-analyses that included studies in which depression was diagnosed by a clinician or was self-reported produced similar results, mean *g*=0.56 and 0.57, respectively.

### Meta-analytical method

Meta-analyses analysed with 3-level models, mean *g*=0.66, 95% CI (0.36, 0.96) with *k*=963, produced larger effect size estimates than meta-analyses analysed with PET-PEESE, mean *g*=0.18, 95% CI (−0.24, 0.59) with *k*=591.

We further investigated the studies in which there was no strong evidence that psychotherapies were effective indicated by including zero in the 95% CI. On closer inspection, we found that the observed null effects were largely caused by using different *How* factors rather than different *Which* factors. Of the 688 meta-analyses that included a zero in their 95% CI, the PET-PEESE method accounted for 408, the RVE estimation method for 143 (as it tends to produce larger CIs), *p*-uniform for 68 and 3-level modelling for 59. The fixed-effect (only one meta-analysis) and REML models (nine meta-analyses) barely produced any statistically non-significant meta-analyses. No other systematic differences were observed among those meta-analyses.

**Figure 3:**
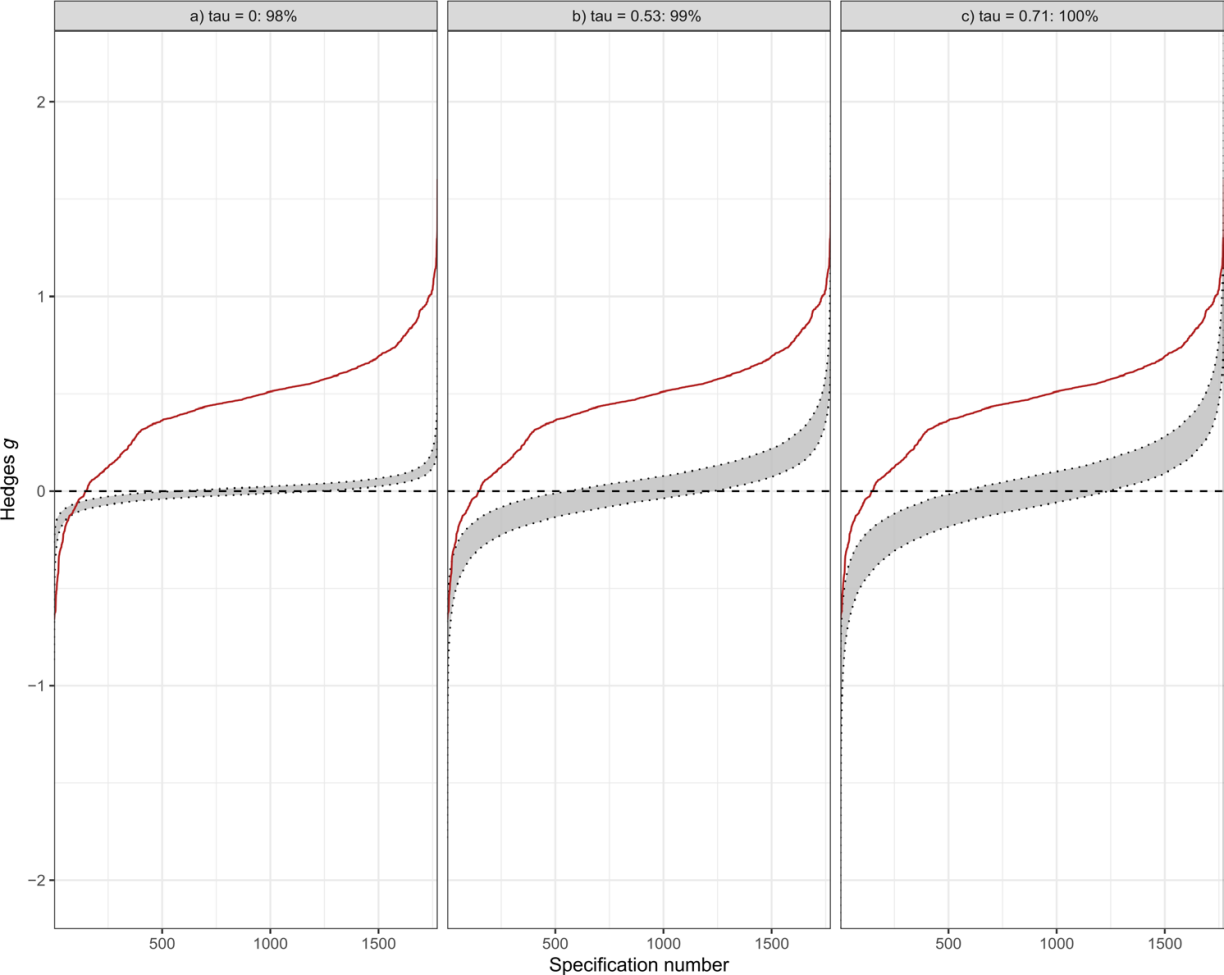
Inferential specification curve. Depicted are three inferential specification curves (red lines), each summarising the magnitude-sorted meta-analytic summary effects and correspond to the descriptive specification curve from figure 2 (for computational reasons only the REML, FE and PET-PEESE models were used). The grey area represents the corresponding 97.5% and 2.5% quantiles of 1000 specification curves that were simulated assuming no true effect. The left panel depicts a fixed-effect scenario of no heterogeneity (*τ*=0), the middle panel a scenario of heterogeneity equal to the random-effects model (*τ*=0.53) and the right panel the scenario of the upper 95% CI estimate of *τ* from the random-effects model (*τ*=0.71). Each is simulated under the null hypothesis for a given specification number using a parametric bootstrap procedure, but they differ in underlying heterogeneity assumptions. If the specification curve exceeds the limits of the 95% CI (as is the case in this plot), there is evidence against the null hypothesis (*g*=0), indicating that there is a substantial effect for the effectiveness of psychotherapies for depression. FE, fixed effect; PET-PEESE, precision-effect test and precision-effect estimate with SE; REM, random-effects model.

The results of the inferential specification curve analysis shown in figure 3 indicate that in most cases, treatments for depression had a substantial effect, as indicated by the deviation from the scenario of no effect (*g*=0). This was true for a range of scenarios, from simulating no heterogeneity (*τ*=0) to the identified heterogeneity in the random-effects model of all 415 studies (*τ*=0.53) and the respective 95% CI upper limit of *τ*=0.71. In fact, 98% of the meta-analyses were outside the expected range under the scenario of no effect, suggesting that the meta-analyses found in the multiverse meta-analysis were significantly different from a null effect. In addition, we conducted a similar analysis for a scenario in which the simulated studies had a clinically relevant effect size of Hedges’ *g*=0.24, rather than *g*=0. We found that, consistent with our previous analysis, most treatments were more effective than the simulated effect sizes in this scenario (see online supplemental eFigure 8).

**Figure 4:**
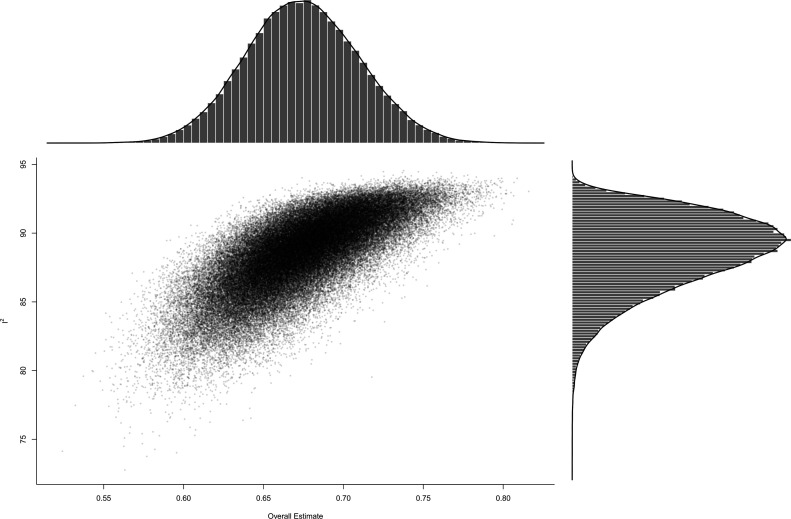
GOSH (Graphical Display of Study Heterogeneity) plot. This GOSH plot visualises the heterogeneity of a random sample of 100 000 subsets for the combinatorial meta-analysis. The y-axis depicts Higgins *I* ^2^ statistics for heterogeneity, and the summary effect size is visualised on the x-axis. Density distributions are visualised next to the respective axes.

The GOSH plot (see figure 4) from 100 000 random samples from all possible subset combinations of 1206 included effect sizes revealed a similar picture as the descriptive specification curve: most meta-analyses fell in the IQR 0.43–0.7 of the multiverse meta-analysis, while heterogeneity is substantial.

We chose to include a conventional 3-level meta-analysis, including all 1206 effect sizes from all 415 primary studies, as an exemplary meta-analytical specification out of the 4281 meta-analyses. The pooled Hedges’ *g* based on this 3-level meta-analytic model was *g*=0.72, 95% CI (0.66, 0.78), p<0.001). The estimated variance components were *τ* ^2^_Level 3_=0.364 and *τ* ^2^_Level 2_=0.004. Overall, *I* ^2^_Level 3_=86% of the total variation can be attributed to between-study and *I* ^2^_Level 2_=1% to within-study heterogeneity. We found that the 3-level model provided a significantly better fit compared with a 2-level model with level 3 heterogeneity constrained to zero (*χ* ^2^ *_1_* =654.03, p<0.001). The overall *I* ^2^ value, indicating how much of the total variance can be attributed to the total amount of heterogeneity, is very large, with approximately 87% of the total variance attributable to heterogeneity.

## Conclusions and clinical implications

We investigated the efficacy of psychotherapies for depression by simultaneously analysing 4281 meta-analyses based on all reasonable combinations of inclusion criteria and meta-analytical methods. We investigated the influence of different treatment groups, types of psychotherapy, treatment formats, control groups, diagnoses for depression, risk of bias assessments and different meta-analytical methods. We found that most meta-analyses produced small-to-medium, but clinically relevant effect sizes, suggesting the overall robustness of psychotherapies for depression. These results are supported by our descriptive and inferential multiverse meta-analysis and the combinatorial meta-analytical approach. Notably, specific patterns emerged in the descriptive specification curve analysis.

Meta-analyses that compared interventions with wait-list control groups had larger effect sizes compared with those including care-as-usual or other control conditions, such as attention placebo. This aligns with previous research suggesting that effect sizes obtained in RCTs should be interpreted differently depending on the used control condition and warning especially against lumping control conditions into one comparison group for network meta-analyses.^14^ It is likely that the larger effect sizes found in meta-analyses that compare interventions with wait-list control groups are due to the fact that wait-list control groups are considered to be less effective treatments than other types of control conditions, such as treatment-as-usual. This means that when interventions are compared with wait-list control groups, the difference in outcome is likely to be greater, resulting in a larger effect size estimate. In other words, the comparison group in these meta-analyses are not as good as other control conditions and therefore the difference with the intervention is greater, which results in a larger effect size.^12 39^ Therefore, it is important to keep in mind that this increase in effect size estimates should be considered when interpreting studies that used wait-list control groups as a comparison.

Our findings indicate that the effect sizes found in depression research might be inflated, even when high risk of bias studies are removed. To support this conclusion, we compared the results of meta-analyses that excluded high risk of bias studies with meta-analyses that did not exclude them. We found that excluding high risk of bias studies did not result in a reduction in effect size estimates. In fact, the effect sizes were very similar to those observed when all studies, regardless of study quality, were included in the meta-analysis. However, when we only included studies with a low risk of bias in our meta-analyses, we did observe a reduction in effect size estimates. This finding is in line with earlier research that has also suggested that effect sizes in depression research may be inflated, as including only low risk of bias studies yields substantially smaller effect size estimates.^13^

Our analysis of publication bias (small-study effects) in psychotherapy research on depression showed that one of the methods used to correct publication bias resulted in substantially smaller effect size estimates compared with conventional methods that did not correct for publication bias. Even though the PET-PEESE estimator may have overcorrected for biases in our data, resulting in negative effect sizes, these negative effect sizes indicate that the meta-analyses under study had no effect when correcting for small-sample effects.^40^ This finding suggests that the previously reported effect sizes for psychotherapy in the treatment of depression may be overestimated and potentially inflated. This is consistent with previous research, which has found significant publication bias for psychotherapies of major depressive disorder,^41^ for digital psychological interventions for depression^42^ and for most evidence-supported therapies for adult depression.^2^ In other words, the true effect size of psychotherapy in treating depression may be smaller than what has been previously reported in the literature.

In addition, we found that meta-analyses focused on student populations and interventions delivered in a group format had higher effect size estimates. This finding is consistent with previous meta-analyses that found interventions to be more effective in young adults compared with middle-aged adults,^43^ although individual formats tend to be at least as effective as group formats.^44^

Our multiverse meta-analysis was able to demonstrate that the inclusion of high and medium risk of bias studies, the comparison with wait-list control groups and models not accounting for publication bias yielded larger—and potentially inflated—effect size estimates for the efficacy of psychotherapies for depression. At the same time, we were able to demonstrate that even after considering this inflation, the effectiveness of psychotherapies remains clinically significant. Despite these strengths of our study, several limitations need to be acknowledged. First, our database contains several studies with unreasonably large effect sizes (Hedges’ *g*>3). These enormous effect sizes might distort meta-analyses with only a few included studies towards more extreme summary effect sizes. For this reason, we included only meta-analyses with more than 10 studies in our multiverse meta-analysis to avoid such extreme meta-analyses. We assessed the influence of several cut-offs in online supplemental eTable 10 and online supplemental eFigure 9. Our sensitivity analyses indicate that the overall mean summary effect size does not change for multiverse meta-analyses limited to at least 10, 25 or 50 primary studies, yet the spread of possible summary effect sizes changes substantially. Second, we had to slightly deviate from our preregistered protocol as we merged several *Which* factor categories to ensure that both computations and visualisations remained feasible. For instance, we initially planned to investigate each therapy type individually, yet we had to create broader categories and combined CBT-based and non-CBT-based therapy approaches together. These merges decreased the level of detail of our analyses, but at the same time ensured the interpretability, as a visualisation of over 40 000 meta-analyses (resulting from not merging different therapy types) was simply not possible. Third, the methods we used for correcting for publication bias have some limitations in performing in environments with low sample size and high heterogeneity—as was the case with the body of evidence in this multiverse meta-analysis. This might have caused the PET-PEESE method to underestimate and *p*-uniform to overestimate the effect size. Overall, the presence of publication bias and its inflating influence on effect size estimates remain highly likely and have been reported in other publications as well.^12 18 45^ It is important to note that the investigated small-study effects can arise from different biases (publication bias, reporting bias), but also might be indicative of a genuine effect. This can, for example, be the case when smaller studies are performed in different populations (ie, difficult to research, high disease burden) or different clinical settings, where the effect is genuinely larger than in larger trials.

Most studies in the present literature only consider one or two of the presented *Which* factors when evaluating the efficacy of psychological treatments for depression. To ensure the comparability of our results with such effect sizes, we also assessed the effect of each ‘Which’ factor separately in our analyses. Presumably, because not all *Which* factors were considered simultaneously as predictors of depressive symptoms, as it is done, for example, in multiple linear regression analyses, effect sizes for each separate factor might have been overestimated. This is true for both the included primary studies and meta-analyses that took such an approach, as well as for the derived effect sizes of our multiverse meta-analysis. This fact, however, again underpins one of the main claims of our study, namely those future meta-analyses that diverge from small-to-medium summary effect size estimates are likely indicative of extreme data analytical decisions and should be evaluated with great care.

In summary, our multiverse meta-analysis completes the evidence that psychotherapies are effective for treating depression in a wide range of patient populations. Because we evaluated the entire multiverse of defensible combinations of inclusion criteria and statistical methods on depression research, our results suggest that this line of research and the debate about whether treatment is effective can now end once and for all. Future research can and should be less concerned with *whether* therapies work but rather investigate *how* they work and who benefits most from which type of intervention. New approaches like individual-patient data meta-analyses and (component) network meta-analyses, as well as longitudinal approaches, are needed to investigate these more relevant and critical issues for the individual patient. Finally, this study provides the most exhaustive overview of psychological depression research that is available and possible today. It can guide future research as knowledge gaps were closed and is a valuable source for policy-makers to inform evidence-based decision-making.

## Data Availability

Data are available in a public, open access repository. The R code and data to reproduce all analyses can be found at the Open Science Framework.
