# Supplementary material for: Exploring the efficacy of psychotherapies for depression: a multiverse meta-analysis
Source: BMJ Ment Health. 2023 Mar 13;26(1):e300626. doi: 10.1136/bmjment-2022-300626 (PMC10035781; doi:10.1136/bmjment-2022-300626)
Supplement: Supplementary data [file bmjment-2022-300626supp001.pdf]

SUPPLEMENTARY MATERIAL TABLE OF CONTENTS

eTables

eTable 1 Detailed description of all included primary studies
eTable 2 Summary characteristics of included primary studies
eTable 3 Distribution of summary effect size estimates for Which factor: Control condition
eTable 4 Distribution of summary effect size estimates for Which factor: Diagnosis
eTable 5 Distribution of summary effect size estimates for Which factor: Format
eTable 6 Distribution of summary effect size estimates for Which factor: Method
eTable 7 Distribution of summary effect size estimates for Which factor: Type
eTable 8 Distribution of summary effect size estimates for Which factor: Risk of bias
eTable 9 Distribution of summary effect size estimates for Which factor: Target Group
eTable 10 Different estimators for effect size-dependency
eTable 11 Sensitivity analyses for multiverse size

eFigures

eFigure 1 Average effect size estimates for Which factor: Control condition
eFigure 2 Average effect size estimates for Which factor: Diagnosis
eFigure 3 Average effect size estimates for Which factor: Format
eFigure 4 Average effect size estimates for Which factor: Method
eFigure 5 Average effect size estimates for Which factor: Type
eFigure 6 Average effect size estimates for Which factor: Risk of bias
eFigure 7 Average effect size estimates for Which factor: Target Group
eFigure 8 Inferential specification curve plot against a simulated clinically relevant effect size
eFigure 9 Sensitivity analyses for multiverse size

eMethods

eMethods 1. Search strings for PubMed.
eMethods 2. Detailed list of Which Factors.
eMethods 3. Deviations from protocol.

eTable 1 Detailed description of all included primary studies

Study	Target group	Intervention	Format	Control	Diagnosis	Risk of bias	Region	N	Hedges´ g	SE
Aagaard, 2017[1]	Adults	CBT-Based	Group	CAU	Diagnosis	Some Concern	Europe	75	0.0000	0.230
Abas, 2018[2]	General Medical	CBT-Based	Individual	CAU	Cut-Off Score	Low	Other Region	28	0.6300	0.390
Ahmadpanah, 2016[3]	General Medical	CBT-Based & Not-CBT-Based	Individual & Group	CAU	Subclinical Depression	Some Concern	Other Region	30	1.6725	0.425
Alexopoulos, 2016[4]	General Medical	CBT-Based	Individual	Other	Diagnosis	Some Concern	North America	168	-0.1964	0.175
Afonso, 2009[5]	Older Adults	Not-CBT-Based	Individual	Other & CAU	Cut-Off Score	Some Concern	Europe	60	2.420	0.345
Alhusen, 2021[6]	Perinatal Depression	CBT-Based	Group	CAU	Cut-Off Score	Some Concern	North America	60	1.460	0.290
Allart Van Dam, 2003[7]	Adults	CBT-Based	Group	CAU	Subclinical Depression	Some Concern	Europe	102	0.5700	0.210
Amani, 2021[8]	Perinatal Depression	CBT-Based	Group	WL	Cut-Off Score	Some Concern	North America	38	1.310	0.360
Ammerman, 2013[9]	Perinatal Depression	CBT-Based	Individual	CAU	Diagnosis	Some Concern	North America	93	0.8797	0.228
Andersson, 2005[10]	Adults	CBT-Based	Guided Self-Help	Other	Cut-Off Score	Some Concern	Europe	85	0.8700	0.230
Araya, 2021[11]	General Medical	CBT-Based	Guided Self-Help	CAU	Cut-Off Score	Low	Other Region	657	0.315	0.095
Arean, 1993[12]	Older Adults	CBT-Based & Not-CBT-Based	Group	WL	Diagnosis	Some Concern	North America	45	0.8756	0.397
Arjadi, 2018[13]	Adults	CBT-Based	Guided Self-Help	Other	Diagnosis	Low	Other Region	276	0.4282	0.125
Ayen, 2004[14]	Other Target Groups	CBT-Based & Not-CBT-Based	Group	WL	Diagnosis	Some Concern	Europe	26	2.4581	0.606
Barber, 2012[15]	Adults	Not-CBT-Based	Individual	Other	Diagnosis	Some Concern	North America	102	0.0769	0.260
Barnhofer, 2009[16]	Adults	CBT-Based	Group	CAU	Diagnosis	Low	Europe	28	1.0423	0.530

Study	Target group	Intervention	Format	Control	Diagnosis	Risk of bias	Region	N	Hedges´ g	SE
Barrett, 2001[17]	Adults	CBT-Based	Individual	Other	Diagnosis	Low	North America	66	0.1542	0.280
Baumeister, 2021[18]	General Medical	CBT-Based	Guided Self-Help	CAU	Diagnosis	Low	Europe	209	0.313	0.140
Baumgartner, 2021[19]	Adults	CBT-Based	Guided Self-Help	WL	Cut-Off Score	Low	Europe	462	0.300	0.090
Beach, 1992[20]	Other Target Groups	CBT-Based	Other Formats & Individual	WL	Diagnosis	Some Concern	North America	30	1.1615	0.400
Bedard, 2014[21]	General Medical	CBT-Based	Group	WL	Cut-Off Score	Some Concern	North America	75	0.3517	0.233
Beeber, 2010[22]	Other Target Groups	Not-CBT-Based	Individual	CAU	Cut-Off Score	Some Concern	North America	70	0.8188	0.265
Bendig, 2021[23]	General Medical	CBT-Based	Guided Self-Help	WL	Cut-Off Score	Low	Europe	34	0.320	0.350
Berger, 2011[24]	Adults	CBT-Based	Guided Self-Help & Other Formats	WL	Diagnosis	Low	Europe	51	0.8819	0.295
Beutel, 2014[25]	General Medical	Not-CBT-Based	Individual	CAU	Diagnosis	Low	Europe	156	0.5200	0.190
Boele, 2018[26]	General Medical	CBT-Based	Guided Self-Help	WL	Cut-Off Score	Low	Europe	53	0.6100	0.290
Boeschoten, 2017[27]	General Medical	CBT-Based	Guided Self-Help	WL	Cut-Off Score	Low	Europe	149	0.1880	0.187
Bohlmeijer, 2011[28]	Adults	CBT-Based	Group	WL	Cut-Off Score	Low	Europe	93	0.6000	0.210
Bolton, 2003[29]	Adults	Not-CBT-Based	Group	CAU	Diagnosis	Some Concern	Other Region	258	1.1484	0.140
Bower, 2021[30]	General Medical	Not-CBT-Based	Group	WL & Other	Cut-Off Score	Some Concern	North America	166	0.145	0.160
Bowman, 1995[31]	Adults	CBT-Based	Guided Self-Help	WL	Cut-Off Score	Some Concern & High	North America	20	0.9458	0.508
Braun, 2021[32]	Other Target Groups	Not-CBT-Based	Guided Self-Help	Other	Cut-Off Score	Low	Europe	340	0.280	0.110

Study	Target group	Intervention	Format	Control	Diagnosis	Risk of bias	Region	N	Hedges´ g	SE
Brown, 1984[33]	Adults	CBT-Based	Group & Individual & Guided Self-Help	WL	Diagnosis	Some Concern	North America	28	0.3934	0.395
Buhrman, 2015[34]	General Medical	CBT-Based	Guided Self-Help	WL	Cut-Off Score	Low	Europe	54	0.5730	0.340
Buntrock, 2015[35]	Adults	Not-CBT-Based	Guided Self-Help	CAU	Subclinical Depression	Low	Europe	405	0.6455	0.113
Burmaoğlu, 2021[36]	Other Target Groups	CBT-Based & Not-CBT-Based	Group	CAU	Diagnosis	Some Concern	Other Region	30	0.360	0.370
Burns, 2007[37]	General Medical	Not-CBT-Based	Individual	CAU	Cut-Off Score	Low	Europe	100	0.4000	0.220
Burns, 2013[38]	Perinatal Depression	CBT-Based	Individual	CAU	Cut-Off Score	Low	Europe	30	0.8731	0.403
Carlbring, 2013[39]	Adults	CBT-Based	Guided Self-Help	WL	Diagnosis	Low	Europe	80	0.7712	0.303
Carr, 2017[40]	Adults	CBT-Based	Group	CAU	Diagnosis	Some Concern	Europe	82	0.3367	0.220
Carta, 2012[41]	Adults	CBT-Based	Individual	CAU	Diagnosis	Some Concern	Europe	64	0.2500	0.250
Casanas, 2012[42]	Adults	CBT-Based	Group	CAU	Diagnosis	Low	Europe	234	0.3490	0.150
Castonguay, 2004[43]	Adults	CBT-Based	Individual	WL	Diagnosis	Some Concern	North America	22	1.6495	0.583
Chan, 2012[44]	Adults	CBT-Based & Not-CBT-Based	Group	WL	Diagnosis	Some Concern	East Asia	33	1.1833	0.400
Chan, 2013[45]	Older Adults	Not-CBT-Based	Individual	CAU	Cut-Off Score	Some Concern	East Asia	26	1.4300	0.440
Chen, 2000[46]	Perinatal Depression	Not-CBT-Based	Group	CAU	Cut-Off Score	Some Concern	East Asia	60	0.6000	0.300
Chesney, 2003[47]	General Medical	Not-CBT-Based	Group	Other & WL	Subclinical Depression	Some Concern	North America	87	0.0920	0.215
Chiang, 2015[48]	Adults	CBT-Based	Group	CAU	Diagnosis	Some Concern	East Asia	62	4.0789	0.460
Cho, 2008[49]	Perinatal Depression	CBT-Based	Individual	CAU	Diagnosis	Some Concern	East Asia	23	0.7876	0.515

Study	Target group	Intervention	Format	Control	Diagnosis	Risk of bias	Region	N	Hedges´ g	SE
Choi, 2012[50]	Other Target Groups	CBT-Based	Guided Self-Help	WL	Diagnosis	Low	Australia	63	0.5429	0.257
Choi, 2014[51]	Older Adults	CBT-Based	Individual & Other Formats	Other	Cut-Off Score	Some Concern	North America	68	0.4000	0.300
Choi, 2020[52]	Other Target Groups	CBT-Based	Other Formats	Other	Cut-Off Score	Some Concern	North America	193	0.8415	0.198
Chowdhary, 2016[53]	Adults	CBT-Based	Individual	CAU	Cut-Off Score	Some Concern	Other Region	50	0.4562	0.300
Choy, 2016[54]	Older Adults	Not-CBT-Based	Group	WL	Cut-Off Score	Some Concern	East Asia	80	0.9679	0.255
Clark, 2003[55]	Perinatal Depression	Not-CBT-Based	Group	WL	Diagnosis	Some Concern	North America	23	0.5310	0.430
Clark, 2008[56]	Perinatal Depression	Not-CBT-Based	Group	WL	Diagnosis	Some Concern	North America	29	1.0900	0.400
Cohen, 2010[57]	Other Target Groups	Not-CBT-Based	Other Formats	WL	Diagnosis	Some Concern	North America	30	0.8371	0.513
Cooper, 2003[58]	Perinatal Depression	CBT-Based & Not-CBT-Based	Individual	CAU	Diagnosis	Low	Europe	92	0.4273	0.220
Cramer, 2011[59]	Other Target Groups	CBT-Based	Group	CAU	Cut-Off Score	Some Concern	Europe	86	0.4757	0.343
D'elia, 2020[60]	Adults	CBT-Based	Group	Other	Diagnosis	Low	North America	16	1.4500	0.580
Davoudi, 2020[61]	General Medical	CBT-Based	Group	Other	Cut-Off Score	Some Concern	Other Region	40	0.7100	0.330
De Groot, 2019[62]	General Medical	CBT-Based	Individual	CAU	Diagnosis	Low	North America	51	0.7908	0.310
De Jong, 2018[63]	General Medical	CBT-Based	Group	WL	Diagnosis	Some Concern	North America	40	0.0600	0.330
Dekker, 2012[64]	General Medical	CBT-Based	Individual	CAU	Cut-Off Score	Low	North America	41	0.4200	0.320
Dennis, 2020[65]	Perinatal Depression	Not-CBT-Based	Other Formats	CAU	Diagnosis	Some Concern	North America	222	0.8501	0.155

Plessen CY, et al. BMJ Ment Health 2023; 26:e300626. doi: 10.1136/bmjment-2022-300626

Study	Target group	Intervention	Format	Control	Diagnosis	Risk of bias	Region	N	Hedges´ g	SE
Derubeis, 2005[66]	Adults	CBT-Based	Individual	Other	Diagnosis	Some Concern	North America	120	0.4500	0.220
Desautels, 2017[67]	General Medical	CBT-Based	Individual	WL	Cut-Off Score	Low	North America	45	1.0041	0.451
Dimidjian, 2006[68]	Adults	CBT-Based	Individual	Other	Diagnosis	Some Concern	North America	82	0.7350	0.338
Dimidjian, 2017[69]	Perinatal Depression	CBT-Based	Other Formats	CAU	Cut-Off Score	Low	North America	146	0.4235	0.187
Dindo, 2012[70]	General Medical	CBT-Based	Group	WL	Diagnosis	Some Concern	North America	54	1.2392	0.432
Dindo, 2019[71]	General Medical	CBT-Based	Group	Other	Diagnosis	Some Concern	North America	110	0.2958	0.240
Dobkin, 2011[72]	General Medical	CBT-Based	Individual	CAU	Diagnosis	Some Concern	North America	81	1.2008	0.283
Dobkin, 2020[73]	General Medical	CBT-Based	Other Formats	CAU	Diagnosis	Low	North America	72	1.0499	0.255
Dobkin, 2021[74]	General Medical	CBT-Based	Other Formats	CAU	Diagnosis	Low	North America	90	0.910	0.220
Doering, 2013[75]	General Medical	CBT-Based	Individual	CAU	Diagnosis	Some Concern	North America	90	1.0268	0.280
Dong, 2019[76]	General Medical	Not-CBT-Based	Other Formats	CAU	Cut-Off Score	Low	East Asia	90	2.2315	0.270
Duarte, 2009[77]	General Medical	CBT-Based	Group	CAU	Diagnosis	Low	Other Region	85	0.8100	0.230
Dwight-Johnson, 2011[78]	Other Target Groups	CBT-Based	Other Formats	CAU	Cut-Off Score	Some Concern	North America	100	1.0628	0.232
Ebert, 2014[79]	Other Target Groups	CBT-Based	Guided Self-Help	WL	Cut-Off Score	Low	Europe	150	0.7580	0.190
Ebert, 2018[80]	Adults	Not-CBT-Based	Guided Self-Help	WL	Subclinical Depression	Low	Europe	189	0.4754	0.166
Ede, 2020[81]	Student Population	CBT-Based	Group	WL	Cut-Off Score	Some Concern	Other Region	162	0.6900	0.160

Study	Target group	Intervention	Format	Control	Diagnosis	Risk of bias	Region	N	Hedges´ g	SE
Ekers, 2011[82]	Adults	CBT-Based	Individual	WL	Diagnosis	Low	Europe	44	0.8173	0.370
Ekkers, 2011[83]	Older Adults	CBT-Based	Group	CAU	Diagnosis	Some Concern	Europe	93	0.8177	0.252
Elkin, 1989[84]	Adults	CBT-Based & Not-CBT-Based	Individual	Other	Diagnosis	Low	North America	121	0.3242	0.196
Embling, 2002[85]	Adults	CBT-Based	Group	WL	Diagnosis	Some Concern	Europe	38	2.4700	0.430
Eseadi, 2018[86]	Other Target Groups	CBT-Based	Group	CAU	Cut-Off Score	Some Concern	Other Region	30	9.5600	1.320
Euteneuer, 2017[87]	Adults	CBT-Based	Individual	WL	Diagnosis	Some Concern	Europe	64	0.7400	0.260
Evans, 1995[88]	General Medical	CBT-Based & Not-CBT-Based	Group	Other	Cut-Off Score	Some Concern	North America	48	0.7530	0.302
Fann, 2015[89]	General Medical	CBT-Based	Individual & Other Formats	CAU	Diagnosis	Some Concern	North America	64	0.1698	0.279
Faramarzi, 2008[90]	Other Target Groups	CBT-Based	Group	CAU	Cut-Off Score	Some Concern	Other Region	58	1.5303	0.350
Fledderus, 2012[91]	Adults	CBT-Based	Guided Self-Help	WL	Cut-Off Score	Some Concern	Europe	250	0.9098	0.185
Floyd, 2004[92]	Older Adults	CBT-Based	Guided Self-Help & Individual	WL	Diagnosis	Some Concern & High	North America	24	1.0631	0.453
Flygare, 2020[93]	Adults	CBT-Based	Guided Self-Help	Other	Cut-Off Score	Low	Europe	75	0.2300	0.230
Folke, 2012[94]	Other Target Groups	CBT-Based	Other Formats	CAU	Diagnosis	Some Concern	Europe	34	0.6600	0.350
Fonagy, 2015[95]	Adults	Not-CBT-Based	Individual	CAU	Diagnosis	Low	Europe	100	0.3906	0.240
Forand, 2018[96]	Adults	CBT-Based	Guided Self-Help	WL	Cut-Off Score	Some Concern	North America	103	1.3944	0.308
Forsell, 2017[97]	Perinatal Depression	CBT-Based	Guided Self-Help	CAU	Diagnosis	Low	Europe	40	0.9008	0.395
Frangou, 2021[98]	General Medical	CBT-Based	Individual	CAU	Cut-Off Score	Some Concern	Europe	67	-0.070	0.240
Freedland, 2009[99]	General Medical	CBT-Based & Not-CBT-Based	Individual	CAU	Diagnosis	Low	North America	82	0.7013	0.245

Study	Target group	Intervention	Format	Control	Diagnosis	Risk of bias	Region	N	Hedges´ g	SE
Freedland, 2015[100]	General Medical	CBT-Based	Individual	CAU	Diagnosis	Low	North America	158	0.6485	0.178
Fry, 1983[101]	Older Adults	Not-CBT-Based	Individual	Other	Cut-Off Score	Some Concern	North America	NA	2.2860	0.260
Fuhr, 2019[102]	Perinatal Depression	CBT-Based	Individual	CAU	Cut-Off Score	Some Concern	Other Region	243	0.2481	0.158
Funderburk, 2021[103]	Other Target Groups	CBT-Based	Individual	CAU	Cut-Off Score	Some Concern	North America	140	0.220	0.170
Furukawa, 2012[104]	Other Target Groups	CBT-Based	Other Formats	WL	Subclinical Depression	Low	East Asia	118	0.6750	0.190
Garcia-Pena, 2015[105]	Older Adults	CBT-Based	Group	CAU	Cut-Off Score	Some Concern	Other Region	82	0.6000	0.260
Gawrysiak, 2009[106]	Student Population	CBT-Based	Individual	CAU	Cut-Off Score	Some Concern	North America	29	1.7035	0.535
Gellis, 2008[107]	General Medical	CBT-Based	Individual	CAU	Subclinical Depression	Some Concern	North America	62	1.6984	0.315
Gellis, 2010[108]	General Medical	CBT-Based	Individual	CAU	Subclinical Depression	Some Concern	North America	36	0.8262	0.345
Geraedts, 2014[109]	Other Target Groups	CBT-Based	Guided Self-Help	CAU	Cut-Off Score	Low	Europe	232	0.1253	0.145
Ghorbani, 2021[110]	General Medical	CBT-Based	Group	WL	Cut-Off Score	Some Concern	Other Region	40	4.500	0.600
Gibbons, 2012[111]	Adults	Not-CBT-Based	Individual	CAU	Cut-Off Score	High & Some Concern	North America	31	0.1014	0.418
Gitlin, 2013[112]	Older Adults	Not-CBT-Based	Individual	WL	Cut-Off Score	Low	North America	188	0.4743	0.163
Goodman, 2015[113]	Perinatal Depression	Not-CBT-Based	Individual	CAU	Cut-Off Score	Some Concern	North America	23	-0.0139	0.660
Greenberg, 2018[114]	Adults	CBT-Based	Group	WL	Cut-Off Score	Some Concern	North America	28	1.0800	0.410
Grote, 2009[115]	Perinatal Depression	Not-CBT-Based	Individual	CAU	Cut-Off Score	Some Concern	North America	50	1.2672	0.395

Study	Target group	Intervention	Format	Control	Diagnosis	Risk of bias	Region	N	Hedges´ g	SE
Hagen, 2017[116]	Adults	CBT-Based	Individual	WL	Diagnosis	Some Concern	Europe	39	2.4700	0.430
Hallford, 2016[117]	Other Target Groups	Not-CBT-Based	Individual	CAU	Cut-Off Score	Some Concern	Australia	27	1.1349	0.480
Hallgren, 2015[118]	Adults	CBT-Based	Guided Self-Help	CAU	Cut-Off Score	Some Concern	Europe	491	0.3200	0.090
Hamamci, 2006[119]	Student Population	CBT-Based	Group	CAU	Cut-Off Score	Some Concern	Other Region	21	1.4050	0.490
Hamdan-Mansour, 2009[120]	Student Population	CBT-Based	Group	CAU	Cut-Off Score	Some Concern	Other Region	84	0.6300	0.220
Han, 2020[121]	Adults	CBT-Based & Not-CBT-Based	Group	Other	Diagnosis	Some Concern	East Asia	33	0.6895	0.357
Haringsma, 2006[122]	Older Adults	CBT-Based	Group	WL	Cut-Off Score	Some Concern	Europe	107	0.4925	0.225
Harley, 2008[123]	Adults	CBT-Based	Group	WL	Diagnosis	Some Concern	North America	19	0.8375	0.485
Harrer, 2021[124]	Student Population	Not-CBT-Based	Guided Self-Help	Other	Cut-Off Score	Low	Europe	200	0.360	0.140
Hassiotis, 2013[125]	Other Target Groups	CBT-Based	Individual	CAU	Cut-Off Score	Some Concern	Europe	30	0.0200	0.370
Hautzinger, 2004[126]	Older Adults	CBT-Based	Group	WL	Diagnosis	Some Concern	Europe	112	0.9845	0.260
Hayman, 1980[127]	Adults	Not-CBT-Based	Group	WL	Cut-Off Score	Some Concern	North America	NA	0.4700	0.390
He, 2019[128]	Adults	CBT-Based	Individual	Other	Diagnosis	Some Concern	East Asia	66	0.4300	0.245
Heckman, 2011[129]	General Medical	Not-CBT-Based	Group	CAU	Cut-Off Score	Some Concern	North America	200	0.3437	0.208
Heckman, 2013[130]	General Medical	Not-CBT-Based	Other Formats	CAU	Cut-Off Score	Some Concern	North America	241	0.2300	0.130
Heckman, 2017[131]	General Medical	Not-CBT-Based	Other Formats	CAU	Diagnosis	Some Concern	North America	140	1.1500	0.430
Hegerl, 2010[132]	Adults	CBT-Based	Group	Other	Diagnosis	Low	Europe	94	0.9200	0.330

Study	Target group	Intervention	Format	Control	Diagnosis	Risk of bias	Region	N	Hedges´ g	SE
Hemanny, 2019[133]	Adults	CBT-Based	Individual	CAU	Diagnosis	Some Concern	Other Region	51	1.2774	0.307
Hermanns, 2015[134]	General Medical	CBT-Based	Group	CAU	Subclinical Depression	Some Concern	Europe	181	0.2000	0.150
Herrmann-Lingen, 2016[135]	General Medical	Not-CBT-Based	Other Formats	CAU	Cut-Off Score	Some Concern	Europe	569	0.0068	0.087
Hoifodt, 2013[136]	Adults	CBT-Based	Guided Self-Help	WL	Cut-Off Score	Some Concern	Europe	105	0.3792	0.320
Holden, 1989[137]	Perinatal Depression	Not-CBT-Based	Individual	CAU	Diagnosis	Some Concern	Europe	52	0.7200	0.330
Honey, 2002[138]	Perinatal Depression	CBT-Based	Group	CAU	Cut-Off Score	Some Concern	Europe	46	0.2903	0.330
Horrell, 2014[139]	Adults	CBT-Based	Group	WL	Cut-Off Score	Low	Europe	382	0.5100	0.100
Hou, 2014[140]	Perinatal Depression	CBT-Based	Other Formats	CAU	Diagnosis	Some Concern	East Asia	213	0.7100	0.140
Hsiao, 2014[141]	Adults	Not-CBT-Based	Group	CAU	Subclinical Depression	Low	East Asia	74	-0.5500	0.240
Huang, 2016[142]	General Medical	CBT-Based	Group	CAU	Cut-Off Score	Some Concern	East Asia	61	1.9500	0.310
Hum, 2019[143]	General Medical	CBT-Based	Other Formats	Other & WL	Cut-Off Score	Some Concern	North America	39	0.0688	0.330
Hummel, 2017[144]	General Medical	CBT-Based	Group	WL	Cut-Off Score	Some Concern	Europe	129	1.3903	0.224
Hunter, 2012[145]	Other Target Groups	CBT-Based	Group	CAU	Cut-Off Score	Some Concern	North America	84	0.4200	0.300
Husain, 2017[146]	Perinatal Depression	CBT-Based	Group	CAU	Diagnosis	Some Concern	Other Region	219	0.8783	0.145
Husain, 2021a[147]	Perinatal Depression	CBT-Based	Group	CAU	Diagnosis	Some Concern	Other Region	769	1.455	0.080
Husain, 2021b[148]	Perinatal Depression	CBT-Based	Group	CAU	Diagnosis	Some Concern	Other Region	97	2.689	0.290

Study	Target group	Intervention	Format	Control	Diagnosis	Risk of bias	Region	N	Hedges´ g	SE
Jalali, 2019[149]	Other Target Groups	Not-CBT-Based	Group	WL	Cut-Off Score	Some Concern	Other Region	42	0.9700	0.330
Jamison, 1995[150]	Adults	CBT-Based	Guided Self-Help	WL	Diagnosis	Some Concern & High	North America	68	1.6160	0.412
Jarrett, 1999[151]	Adults	CBT-Based	Individual	Other	Diagnosis	Some Concern	North America	72	0.5958	0.268
Jelinek, 2016[152]	Adults	CBT-Based	Group	Other	Diagnosis	Some Concern	Europe	65	0.4832	0.283
Jesse, 2015[153]	Perinatal Depression	CBT-Based	Group	CAU	Cut-Off Score	Some Concern	North America	56	0.3481	0.297
Jiang, 2014[154]	Perinatal Depression	Not-CBT-Based	Other Formats	CAU	Cut-Off Score	Some Concern	East Asia	771	1.0500	0.080
Johansson, 2012a[155]	Adults	Not-CBT-Based	Guided Self-Help	Other	Diagnosis	Low	Europe	92	0.8838	0.248
Johansson, 2012b[156]	Adults	CBT-Based	Guided Self-Help	Other	Diagnosis	Some Concern	Europe	72	0.8233	0.289
Johansson, 2019[157]	Adults	CBT-Based	Guided Self-Help	WL	Diagnosis	Low	Europe	54	1.5650	0.310
Johnson, 2012[158]	Other Target Groups	Not-CBT-Based	Other Formats	Other	Diagnosis	Some Concern	North America	38	0.7521	0.410
Johnson, 2019[159]	Other Target Groups	Not-CBT-Based	Other Formats	CAU	Diagnosis	Low	North America	NA	0.1950	0.150
Joling, 2011[160]	Older Adults	CBT-Based	Guided Self-Help	CAU	Subclinical Depression	Low	Europe	171	-0.0794	0.207
Jordans, 2019[161]	Adults	CBT-Based	Individual	CAU	Diagnosis	Low	Other Region	120	0.4341	0.200
Kamga, 2017[162]	General Medical	CBT-Based	Guided Self-Help	WL	Cut-Off Score	Low	North America	70	0.4300	0.240
Kanter, 2015[163]	Other Target Groups	CBT-Based	Individual	CAU	Diagnosis	Some Concern	North America	40	0.2890	0.360
Keeley, 2016[164]	Adults	Not-CBT-Based	Individual	CAU	Diagnosis	Low	North America	172	0.4702	0.215
Kelly, 1993[165]	General Medical	CBT-Based & Not-CBT-Based	Group	Other	Cut-Off Score	Some Concern	North America	48	0.6960	0.315

Study	Target group	Intervention	Format	Control	Diagnosis	Risk of bias	Region	N	Hedges´ g	SE
Kenter, 2016[166]	Adults	CBT-Based	Guided Self-Help	WL	Diagnosis	Low	Europe	269	-0.0700	0.120
Kessler, 2009[167]	Adults	CBT-Based	Guided Self-Help	WL	Diagnosis	Low	Europe	218	0.5130	0.155
Kim, 2018[168]	General Medical	CBT-Based	Other Formats	CAU	Cut-Off Score	Some Concern	East Asia	60	0.8200	0.270
King, 2000[169]	Adults	CBT-Based & Not-CBT-Based	Individual	CAU	Cut-Off Score	Some Concern	Europe	132	0.4150	0.180
Kivi, 2014[170]	Adults	CBT-Based	Guided Self-Help	CAU	Diagnosis	Some Concern	Europe	86	-0.1600	0.260
Kleiboer, 2015[171]	Adults	CBT-Based & Not-CBT-Based	Other Formats & Guided Self-Help	WL	Cut-Off Score	Low	Europe	214	0.2313	0.140
Korrelboom, 2012[172]	Adults	Not-CBT-Based	Group	WL	Diagnosis	Low	Europe	62	0.7884	0.313
Korte, 2012[173]	Older Adults	Not-CBT-Based	Group	WL	Cut-Off Score	Low	Europe	169	0.5878	0.180
Kramer, 2021[174]	Adults	CBT-Based	Guided Self-Help	WL	Cut-Off Score	Some Concern	Europe	136	0.520	0.170
Laidlaw, 2008[175]	Older Adults	CBT-Based	Individual	CAU	Diagnosis	Some Concern	Europe	40	0.4692	0.333
Lamers, 2010[176]	General Medical	CBT-Based	Individual	CAU	Diagnosis	Low	Europe	229	0.2503	0.180
Lamers, 2015[177]	Adults	Not-CBT-Based	Guided Self-Help	WL	Cut-Off Score	Low	Europe	116	0.3400	0.190
Landreville, 1997[178]	Older Adults	CBT-Based	Guided Self-Help	WL	Cut-Off Score	Some Concern	North America	22	0.3243	0.513
Lappalainen, 2015[179]	Adults	CBT-Based	Guided Self-Help	WL	Diagnosis	Some Concern	Europe	37	0.6635	0.467
Larcombe, 1984[180]	General Medical	CBT-Based	Group	WL	Diagnosis	Some Concern	Australia	19	2.6939	0.650
Lee, 2021[181]	Adults	CBT-Based	Group	CAU	Diagnosis	Low	East Asia	50	0.632	0.380
Lemma, 2013[182]	Adults	Not-CBT-Based	Other Formats	Other	Cut-Off Score	Some Concern	Europe	16	-0.1950	0.500
Lenze, 2017[183]	Perinatal Depression	Not-CBT-Based	Individual	CAU	Diagnosis	Low	North America	40	-0.2904	0.340
Lenze, 2020[184]	Perinatal Depression	Not-CBT-Based	Individual	CAU	Diagnosis	Low	North America	30	-0.1400	0.370

Plessen CY, *et al. BMJ Ment Health* 2023; 26:e300626. doi: 10.1136/bmjment-2022-300626

Study	Target group	Intervention	Format	Control	Diagnosis	Risk of bias	Region	N	Hedges´ g	SE
Lerner, 2015[185]	Other Target Groups	CBT-Based	Other Formats	CAU	Diagnosis	Some Concern	North America	276	0.5253	0.160
Leung, 2013[186]	Perinatal Depression	CBT-Based	Group	CAU	Cut-Off Score	Some Concern	East Asia	97	0.3053	0.205
Leung, 2016[187]	Perinatal Depression	CBT-Based	Group	Other	Cut-Off Score	Some Concern	East Asia	164	0.1550	0.160
Lexis, 2011[188]	Other Target Groups	CBT-Based	Individual	CAU	Cut-Off Score	Some Concern	Europe	113	0.3161	0.213
Liang, 2021[189]	Student Population	Not-CBT-Based	Group	Other	Cut-Off Score	Some Concern	East Asia	52	0.550	0.285
Linde, 2011[190]	Other Target Groups	CBT-Based	Group	Other	Cut-Off Score	Low	North America	NA	0.1500	0.140
Liu, 2009[191]	Adults	CBT-Based	Guided Self-Help	WL	Cut-Off Score	Some Concern	East Asia	51	0.2440	0.305
Liu, 2021[192]	Perinatal Depression	CBT-Based	Individual	CAU	Subclinical Depression	Some Concern	East Asia	228	1.490	0.150
Lloyd-Williams, 2018[193]	General Medical	Not-CBT-Based	Individual	CAU	Cut-Off Score	Some Concern	Europe	37	0.2818	0.380
Lök, 2019[194]	General Medical	Not-CBT-Based	Group	CAU	Cut-Off Score	Some Concern	Other Region	60	1.7600	0.310
Losada, 2015[195]	Other Target Groups	CBT-Based	Individual	Other	Cut-Off Score	Some Concern	Europe	77	0.7715	0.258
Lovell, 2008[196]	Adults	CBT-Based	Individual	CAU	Cut-Off Score	Some Concern	Europe	41	0.1829	0.315
Lund, 2019[197]	Perinatal Depression	Not-CBT-Based	Individual	CAU	Cut-Off Score	Low	Other Region	277	0.1183	0.127
Lundgren, 2016[198]	General Medical	CBT-Based	Guided Self-Help	Other	Cut-Off Score	Low	Europe	50	0.2700	0.280
Lustman, 1998[199]	General Medical	CBT-Based	Individual	CAU	Diagnosis	Low	North America	44	1.0944	0.378
Lynch, 1997[200]	Adults	CBT-Based	Other Formats	CAU	Subclinical Depression	Some Concern	North America	24	0.9300	0.430

Study	Target group	Intervention	Format	Control	Diagnosis	Risk of bias	Region	N	Hedges´ g	SE
Lynch, 2004[201]	Adults	CBT-Based	Other Formats	CAU & Other	Cut-Off Score	Some Concern	North America	20	0.0413	0.453
Lynch, 2019[202]	Adults	Not-CBT-Based	Other Formats	CAU	Diagnosis	Low	Europe	254	0.5786	0.188
Maclean, 2020[203]	Adults	Not-CBT-Based	Guided Self-Help	Other	Cut-Off Score	Some Concern	North America	93	0.1700	0.210
Macpherson, 2013[204]	Adults	Not-CBT-Based	Individual	CAU	Cut-Off Score	Low	Europe	364	0.2300	0.110
Mahmoodi, 2021[205]	Adults	CBT-Based	Individual	WL	Cut-Off Score	Some Concern	Other Region	42	1.232	0.345
Maina, 2005[206]	Adults	Not-CBT-Based	Individual	WL	Diagnosis	Some Concern	Europe	20	1.1194	0.485
Malouff, 1988[207]	Other Target Groups	CBT-Based	Group	WL	Cut-Off Score	Some Concern	North America	28	1.2855	0.417
Martin, 2015[208]	General Medical	CBT-Based	Individual	WL	Diagnosis	Some Concern	Australia	40	1.2395	0.390
Matsuzaka, 2017[209]	Adults	Not-CBT-Based	Individual	CAU	Diagnosis	Low	Other Region	78	0.0758	0.264
Mcclay, 2015[210]	Adults	CBT-Based	Other Formats	WL	Cut-Off Score	Some Concern	Europe	NA	0.8500	0.450
Mccusker, 2021[211]	General Medical	CBT-Based	Guided Self-Help	CAU	Cut-Off Score	Some Concern	North America	244	0.688	0.170
Mcindoo, 2016[212]	Student Population	CBT-Based	Individual	WL	Cut-Off Score	Some Concern	North America	35	0.9383	0.431
Mckee, 2006[213]	Perinatal Depression	CBT-Based	Individual	CAU	Cut-Off Score	Some Concern	North America	41	0.0700	0.310
Mennen, 2021[214]	Perinatal Depression	Not-CBT-Based	Group	CAU	Cut-Off Score	Some Concern	North America	119	0.390	0.190
Michalak, 2015[215]	Adults	Not-CBT-Based & CBT-Based	Other Formats & Group	CAU	Diagnosis	Low	Europe	63	0.5742	0.332
Milgrom, 2005[216]	Perinatal Depression	CBT-Based & Not-CBT-Based	Group & Individual	CAU	Diagnosis	Low	Australia	69	0.7194	0.347
Milgrom, 2011[217]	Perinatal Depression	CBT-Based	Individual	CAU	Cut-Off Score	Low	Australia	46	0.3407	0.300

Study	Target group	Intervention	Format	Control	Diagnosis	Risk of bias	Region	N	Hedges´ g	SE
Milgrom, 2015[218]	Perinatal Depression	CBT-Based	Individual	CAU	Diagnosis	Low	Australia	44	0.5700	0.310
Milgrom, 2016[219]	Perinatal Depression	CBT-Based	Guided Self-Help	CAU	Diagnosis	Low	Australia	40	1.0795	0.375
Milgrom, 2020[220]	Perinatal Depression	CBT-Based	Guided Self-Help	CAU	Diagnosis	Low	Australia	42	1.0173	0.360
Milgrom, 2021[221]	Perinatal Depression	CBT-Based	Guided Self-Help & Individual	CAU	Diagnosis	Low	Australia	77	0.244	0.235
Miller, 2002[222]	Adults	Not-CBT-Based	Other Formats	CAU	Cut-Off Score	Some Concern	North America	30	0.4500	0.370
Miranda, 2003[223]	Other Target Groups	CBT-Based	Other Formats	CAU	Diagnosis	Low	North America	179	0.1600	0.150
Mohr, 2000[224]	General Medical	CBT-Based	Other Formats	CAU	Cut-Off Score	Some Concern	North America	32	0.5700	0.360
Mohr, 2011[225]	Other Target Groups	CBT-Based	Other Formats	CAU	Diagnosis	Some Concern	North America	80	0.2361	0.233
Mohr, 2013[226]	Adults	CBT-Based	Guided Self-Help & Other Formats	WL	Diagnosis	Low	North America	62	0.4968	0.272
Moldovan, 2013[227]	Student Population	CBT-Based	Guided Self-Help	CAU & Other & WL	Cut-Off Score	Low	Europe	42	0.5433	0.347
Montero-Marín, 2016[228]	Adults	CBT-Based	Other Formats & Guided Self-Help	CAU	Diagnosis	Low	Europe	128	0.0989	0.175
Moon, 2021[229]	General Medical	CBT-Based	Group	CAU	Cut-Off Score	Low	East Asia	39	1.881	0.390
Mossey, 1996[230]	Older Adults	Not-CBT-Based	Individual	CAU	Subclinical Depression	Some Concern	North America	62	0.2100	0.280
Mukhtar, 2011[231]	Adults	CBT-Based	Group	WL	Diagnosis	Some Concern	East Asia	113	4.8300	0.370
Mulcahy, 2010[232]	Perinatal Depression	Not-CBT-Based	Other Formats	CAU	Diagnosis	Some Concern	Australia	48	0.6209	0.373
Musa, 2021[233]	Other Target Groups	CBT-Based	Group	CAU	Diagnosis	Low	Other Region	100	0.490	0.200

Plessen CY, et al. BMJ Ment Health 2023; 26:e300626. doi: 10.1136/bmjment-2022-300626

Study	Target group	Intervention	Format	Control	Diagnosis	Risk of bias	Region	N	Hedges´ g	SE
Mynors-Wallis, 1995[234]	Adults	CBT-Based	Individual	Other	Diagnosis	Some Concern	Europe	58	0.6807	0.292
Naeem, 2013[235]	Adults	CBT-Based	Guided Self-Help	CAU	Diagnosis	Low	Other Region	186	1.1863	0.180
Naeem, 2015[236]	Adults	CBT-Based	Other Formats	CAU	Diagnosis	Low	Other Region	137	0.8600	0.180
Nakagawa, 2017[237]	Adults	CBT-Based	Individual	CAU	Diagnosis	Low	East Asia	80	0.5165	0.246
Nakimuli-Mpungu, 2015[238]	General Medical	Not-CBT-Based	Group	Other	Diagnosis	Low	Other Region	109	0.0500	0.190
Nakimuli-Mpungu, 2020[239]	General Medical	CBT-Based	Group	Other	Diagnosis	Low	Other Region	1156	2.6100	0.390
Nasrin, 2017[240]	Adults	CBT-Based	Individual	WL	Diagnosis	Some Concern	Europe	36	1.7400	0.390
Neugebauer, 2006[241]	Other Target Groups	Not-CBT-Based	Other Formats	CAU	Subclinical Depression	Some Concern	North America	19	0.1500	0.460
Newby, 2014[242]	Adults	CBT-Based	Other Formats & Individual	CAU	Cut-Off Score	Some Concern	Australia	40	0.2750	0.320
Newby, 2017[243]	General Medical	CBT-Based	Guided Self-Help	WL	Diagnosis	Low	Australia	72	0.9841	0.260
Nezu, 1986[244]	Adults	CBT-Based	Group	WL	Diagnosis	Some Concern	North America	17	1.3862	0.714
Nezu, 1989[245]	Adults	CBT-Based	Group	WL	Diagnosis	Some Concern	North America	26	1.8821	0.600
Ng, 2013[246]	Older Adults	Not-CBT-Based	Individual	CAU	Cut-Off Score	Some Concern	East Asia	26	1.4300	0.440
Ngai, 2015[247]	Perinatal Depression	CBT-Based	Other Formats	CAU	Cut-Off Score	Low	East Asia	397	0.4509	0.107
Niedermoser, 2020[248]	Other Target Groups	Not-CBT-Based	Group	CAU	Diagnosis	Low & Some Concern	Europe	27	0.5300	0.390
Nobis, 2015[249]	General Medical	Not-CBT-Based	Guided Self-Help	Other	Cut-Off Score	Some Concern	Europe	257	0.9708	0.155
Nollett, 2016[250]	General Medical	CBT-Based	Individual	CAU & WL	Cut-Off Score	Some Concern	Europe	44	-0.1233	0.325

Study	Target group	Intervention	Format	Control	Diagnosis	Risk of bias	Region	N	Hedges´ g	SE
Northwood, 2020[251]	Other Target Groups	Not-CBT-Based	Individual	CAU	Diagnosis	Low	North America	214	0.9200	0.140
Nyström, 2017[252]	Adults	CBT-Based	Guided Self-Help	WL	Diagnosis	Low	Europe	109	0.8100	0.200
O'Hara, 2000[253]	Perinatal Depression	Not-CBT-Based	Individual	WL	Diagnosis	Some Concern	North America	102	0.9657	0.257
O'Mahen, 2013[254]	Perinatal Depression	CBT-Based	Individual	CAU	Diagnosis	Low	Europe	58	2.2879	0.567
O'Neil, 2014[255]	General Medical	CBT-Based	Other Formats	CAU	Cut-Off Score	Low	Australia	121	0.1150	0.180
Oehler, 2020[256]	Adults	CBT-Based	Guided Self-Help	Other	Diagnosis	Some Concern	Europe	263	0.1800	0.120
Olukolade, 2017[257]	General Medical	CBT-Based & Not-CBT-Based	Individual	WL	Cut-Off Score	Some Concern	Other Region	20	0.6981	0.500
Omidi, 2013[258]	Adults	CBT-Based	Group	CAU	Diagnosis	Some Concern	Other Region	60	1.5783	0.295
Onuigbo, 2019[259]	General Medical	CBT-Based	Group	CAU	Cut-Off Score	Low	Other Region	65	1.7400	0.290
Onyechi, 2016[260]	General Medical	CBT-Based	Individual	Other	Cut-Off Score	Some Concern	Other Region	80	10.3177	1.005
Pace, 1993[261]	Student Population	CBT-Based	Individual	WL	Cut-Off Score	Some Concern	North America	68	0.8335	0.265
Pagoto, 2013[262]	Other Target Groups	CBT-Based	Other Formats	Other	Diagnosis	Low	North America	136	0.4300	0.190
Patel, 2017[263]	Adults	CBT-Based	Individual	CAU	Cut-Off Score	Low	Other Region	463	0.5400	0.100
Pecheur, 1984[264]	Student Population	CBT-Based	Individual	WL	Diagnosis	Some Concern	North America	14	1.9141	0.662
Peden, 2000[265]	Student Population	CBT-Based	Group	CAU	Cut-Off Score	Some Concern	North America	74	1.1300	0.320
Penckofer, 2012[266]	General Medical	CBT-Based	Group	CAU	Cut-Off Score	Some Concern	North America	70	0.6501	0.265
Perini, 2009[267]	Adults	CBT-Based	Guided Self-Help	WL	Diagnosis	Some Concern	Australia	47	0.6168	0.390

Study	Target group	Intervention	Format	Control	Diagnosis	Risk of bias	Region	N	Hedges´ g	SE
Petersen, 2014[268]	General Medical	Not-CBT-Based	Group	CAU	Diagnosis	Some Concern	Other Region	34	0.9200	0.360
Pibernik-Okanovic, 2009[269]	General Medical	CBT-Based	Group	CAU	Cut-Off Score	Some Concern	Europe	NA	-0.1400	0.080
Pibernik-Okanovic, 2015[270]	General Medical	CBT-Based	Group	CAU	Subclinical Depression	Low	Europe	121	0.0800	0.180
Pihlaja, 2020[271]	Adults	CBT-Based	Guided Self-Help	CAU	Diagnosis	Some Concern	Europe	100	0.4700	0.200
Poleshuck, 2014[272]	General Medical	Not-CBT-Based	Individual	CAU	Diagnosis	Some Concern	North America	50	0.1700	0.280
Pot, 2010[273]	Older Adults	Not-CBT-Based	Group	Other	Subclinical Depression	Some Concern	Europe	171	0.3900	0.150
Pots, 2014[274]	Adults	CBT-Based	Group	WL	Cut-Off Score	Low	Europe	146	0.5324	0.200
Pots, 2016[275]	Adults	CBT-Based	Guided Self-Help	WL	Cut-Off Score	Low	Europe	166	0.5953	0.170
Power-Michael, 2012[276]	Adults	CBT-Based & Not-CBT-Based	Individual	WL	Diagnosis	Some Concern	Europe	40	0.5181	0.370
Prendergast, 2001[277]	Perinatal Depression	CBT-Based	Individual	Other	Diagnosis	Some Concern	Australia	36	0.1208	0.373
Preschl, 2012[278]	Older Adults	Not-CBT-Based	Individual	WL	Cut-Off Score	Some Concern	Europe	36	0.7100	0.350
Propst, 1992[279]	Other Target Groups	CBT-Based	Individual	CAU & WL	Cut-Off Score	Some Concern	North America	24	0.5267	0.472
Psarraki, 2021[280]	Adults	Not-CBT-Based	Other Formats	CAU	Diagnosis	Some Concern	Europe	62	0.535	0.260
Puckering, 2010[281]	Perinatal Depression	Not-CBT-Based	Group	WL	Cut-Off Score	Some Concern	Europe	14	0.8200	0.610
Pugh, 2016[282]	Perinatal Depression	CBT-Based	Guided Self-Help	WL	Cut-Off Score	Some Concern	North America	41	0.8192	0.337
Qiu, 2013[283]	General Medical	CBT-Based	Group	WL	Diagnosis	Low	East Asia	62	2.1700	0.320
Raevuori, 2021[284]	Student Population	Not-CBT-Based	Guided Self-Help	CAU	Diagnosis	Low	Europe	124	-0.260	0.180

Study	Target group	Intervention	Format	Control	Diagnosis	Risk of bias	Region	N	Hedges´ g	SE
Rahman, 2008[285]	Perinatal Depression	CBT-Based	Group	Other	Diagnosis	Low	Other Region	811	0.7287	0.075
Ransom, 2008[286]	General Medical	Not-CBT-Based	Other Formats	CAU	Diagnosis	Some Concern	North America	71	0.3669	0.415
Raue, 2019[287]	Older Adults	CBT-Based	Individual	CAU	Cut-Off Score	High	North America	14	0.7900	0.560
Raya-Tena, 2021[288]	General Medical	Not-CBT-Based	Group	CAU	Cut-Off Score	Some Concern	Europe	394	0.142	0.115
Rehm, 1981[289]	Adults	Not-CBT-Based	Group	WL	Cut-Off Score	Some Concern	North America	23	0.4814	0.452
Reins, 2019[290]	Adults	Not-CBT-Based	Guided Self-Help	Other	Diagnosis	Low	Europe	130	0.2750	0.217
Richards, 2015[291]	Adults	CBT-Based	Guided Self-Help	WL	Cut-Off Score	Some Concern	Europe	191	0.7691	0.250
Richards, 2018[292]	General Medical	CBT-Based	Individual	CAU	Cut-Off Score	Low	Europe	22	-0.4185	0.520
Rief, 2018[293]	Adults	Not-CBT-Based & CBT-Based	Individual	WL	Diagnosis	Low	Europe	86	0.8734	0.403
Ritvo, 2021[294]	Adults	CBT-Based	Guided Self-Help	CAU	Diagnosis	Low	North America	45	0.561	0.305
Rizvi, 2015[295]	Adults	CBT-Based	Individual	WL	Diagnosis	Some Concern	North America	36	0.1419	0.370
Rohan, 2007[296]	Adults	CBT-Based	Group & Other Formats	WL	Diagnosis	Some Concern	North America	30	1.2087	0.469
Rohde, 2016[297]	Student Population	CBT-Based	Group	Other	Subclinical Depression	Some Concern	North America	59	0.7050	0.270
Rohricht, 2013[298]	Adults	Not-CBT-Based	Group	WL	Diagnosis	Low	Europe	23	0.9200	0.440
Ross, 1985[299]	Adults	CBT-Based	Other Formats	WL	Diagnosis	Some Concern	Europe	41	1.4750	0.350
Rosso, 2017[300]	Adults	CBT-Based	Guided Self-Help	Other	Diagnosis	Some Concern	North America	76	0.9269	0.268
Rude, 1986[301]	Adults	Not-CBT-Based	Group	WL	Cut-Off Score	Some Concern	North America	NA	1.0300	0.330

Study	Target group	Intervention	Format	Control	Diagnosis	Risk of bias	Region	N	Hedges´ g	SE
Russell, 2019[302]	Other Target Groups	CBT-Based	Guided Self-Help	CAU	Cut-Off Score	Some Concern	Europe	51	0.3171	0.290
Ruwaard, 2009[303]	Adults	CBT-Based	Guided Self-Help	WL	Cut-Off Score	Some Concern	Europe	61	0.7113	0.366
Ruzickova, 2021[304]	Adults	CBT-Based	Other Formats	CAU	Cut-Off Score	Some Concern	Europe	68	0.640	0.250
Safren, 2009[305]	General Medical	CBT-Based	Individual	WL	Diagnosis	Some Concern	North America	45	0.6425	0.305
Safren, 2014[306]	General Medical	CBT-Based	Individual	CAU	Diagnosis	Some Concern	North America	87	0.6500	0.220
Safren, 2016[307]	General Medical	CBT-Based & Not-CBT-Based	Individual	CAU	Diagnosis	Some Concern	North America	144	0.3850	0.180
Safren, 2021[308]	General Medical	CBT-Based	Individual	CAU	Diagnosis	Some Concern	Other Region	161	0.640	0.160
Salamanca-Sanabria, 2020[309]	Student Population	CBT-Based	Guided Self-Help	WL	Cut-Off Score	Some Concern	Other Region	58	0.9642	0.300
Saloheimo, 2016[310]	Adults	Not-CBT-Based & CBT-Based	Individual & Group	CAU	Diagnosis	Some Concern	Europe	88	0.2202	0.235
Sander, 2020[311]	General Medical	CBT-Based	Guided Self-Help	CAU	Subclinical Depression	Low	Europe	297	0.2819	0.193
Savard, 2006[312]	General Medical	CBT-Based	Individual	WL	Cut-Off Score	Some Concern	North America	37	0.6449	0.343
Savari, 2021[313]	Student Population	CBT-Based	Group	WL	Diagnosis	Some Concern	Other Region	30	1.650	0.430
Schlicker, 2020[314]	General Medical	CBT-Based	Guided Self-Help	WL	Diagnosis	Low	Europe	77	0.3424	0.243
Schmidt, 1983[315]	Adults	CBT-Based	Guided Self-Help & Individual & Group	WL	Cut-Off Score	Some Concern	North America	22	1.1474	0.475
Schramm, 2020[316]	Other Target Groups	Not-CBT-Based	Group	CAU	Diagnosis	Some Concern	Europe	27	0.2756	0.427
Schulberg, 1996[317]	Adults	Not-CBT-Based	Individual	CAU	Diagnosis	Some Concern	North America	184	0.5744	0.170
Schuster, 2017[318]	Adults	Not-CBT-Based	Group	WL	Cut-Off Score	Some Concern	Europe	45	0.8980	0.345

Study	Target group	Intervention	Format	Control	Diagnosis	Risk of bias	Region	N	Hedges´ g	SE
Scogin, 1987[319]	Older Adults	CBT-Based	Guided Self-Help	Other & WL	Cut-Off Score	Some Concern & High	North America	17	1.0559	0.525
Scogin, 1989[320]	Older Adults	CBT-Based	Guided Self-Help	WL	Cut-Off Score	Some Concern & High	North America	41	0.6585	0.330
Scogin, 2014[321]	Older Adults	CBT-Based	Individual	Other	Cut-Off Score	Low	North America	134	0.3767	0.170
Scott, 1990[322]	Adults	CBT-Based	Group & Individual	WL	Diagnosis	Some Concern	Europe	37	0.7876	0.388
Scott, 1992[323]	Adults	CBT-Based & Not-CBT-Based	Individual	CAU	Diagnosis	Some Concern	Europe	58	0.2955	0.282
Scott, 1997[324]	Adults	CBT-Based	Individual	CAU	Diagnosis	Some Concern	Europe	35	0.5661	0.380
Segre, 2015[325]	Other Target Groups	Not-CBT-Based	Individual	WL	Cut-Off Score	Some Concern	North America	69	0.4187	0.305
Selmi, 1990[326]	Adults	CBT-Based	Guided Self-Help & Individual	WL	Diagnosis	Some Concern	North America	24	1.0551	0.508
Serfaty, 2009[327]	Older Adults	CBT-Based	Individual	CAU & Other	Diagnosis	Low	Europe	120	0.1750	0.180
Serfaty, 2019[328]	General Medical	CBT-Based	Individual	CAU	Diagnosis	Low	Europe	148	0.0946	0.165
Serrano-Selva, 2012[329]	Older Adults	Not-CBT-Based	Individual	Other	Diagnosis	Some Concern	Europe	17	0.3850	0.490
Serrano, 2004[330]	Older Adults	Not-CBT-Based	Individual	CAU	Cut-Off Score	Some Concern	Europe	43	0.9500	0.320
Shaw, 1977[331]	Student Population	CBT-Based	Group	WL & Other	Cut-Off Score	Some Concern	North America	16	1.2453	0.533
Sheeber, 2012[332]	Perinatal Depression	CBT-Based	Guided Self-Help	WL	Cut-Off Score	Low	North America	62	0.8681	0.295
Sheeber, 2017[333]	Perinatal Depression	CBT-Based	Guided Self-Help	Other	Cut-Off Score	Some Concern	North America	266	0.3100	0.120
Shih, 2021[334]	Older Adults	CBT-Based	Group	Other	Cut-Off Score	Some Concern	East Asia	57	0.510	0.270
Sikander, 2019[335]	Perinatal Depression	CBT-Based	Other Formats	CAU	Cut-Off Score	Low	Other Region	433	0.2219	0.110

Study	Target group	Intervention	Format	Control	Diagnosis	Risk of bias	Region	N	Hedges´ g	SE
Simoni, 2013[336]	General Medical	CBT-Based	Individual	CAU	Cut-Off Score	Low	North America	40	-0.1600	0.320
Simpson, 2003[337]	Adults	Not-CBT-Based	Individual	CAU	Diagnosis	Low	Europe	130	0.0116	0.190
Simson, 2008[338]	General Medical	Not-CBT-Based	Individual	CAU	Cut-Off Score	Some Concern	Europe	30	0.2300	0.370
Sinniah, 2017[339]	Other Target Groups	CBT-Based	Individual	CAU	Diagnosis	Some Concern	East Asia	68	0.6936	0.285
Sirey, 2021[340]	Other Target Groups	Not-CBT-Based	Individual	CAU	Cut-Off Score	Some Concern	North America	50	0.790	0.480
Smit, 2006[341]	Adults	CBT-Based & Not-CBT-Based	Individual	CAU	Diagnosis	Low	Europe	109	-0.0806	0.253
Smith, 2017[342]	Adults	CBT-Based	Other Formats & Guided Self-Help	WL	Diagnosis	Low	Australia	101	0.6292	0.220
Songprakun, 2012[343]	Adults	CBT-Based	Guided Self-Help	CAU	Diagnosis	Low	East Asia	54	0.6000	0.270
Spek, 2007[344]	Older Adults	CBT-Based	Group & Other Formats	WL	Subclinical Depression	Low	Europe	144	0.3411	0.195
Spinelli, 2003[345]	Perinatal Depression	Not-CBT-Based	Individual	Other	Diagnosis	Some Concern	North America	42	0.7370	0.425
Spinelli, 2013[346]	Perinatal Depression	Not-CBT-Based	Other Formats	Other	Diagnosis	Some Concern	North America	86	-0.1500	0.250
Spruill, 2021[347]	General Medical	Not-CBT-Based	Other Formats	CAU	Cut-Off Score	Low	North America	67	0.120	0.240
Sreevani, 2013[348]	Adults	Not-CBT-Based	Group	CAU	Diagnosis	Some Concern	Other Region	30	0.9200	0.380
Stiles-Shields, 2018[349]	Adults	CBT-Based	Guided Self-Help	WL	Cut-Off Score	Some Concern	North America	18	1.1697	0.515
Strauss, 2012[350]	Adults	CBT-Based	Group	CAU	Diagnosis	Some Concern	Europe	28	1.8300	0.450
Strong, 2008[351]	General Medical	CBT-Based	Individual	CAU	Diagnosis	Low	Europe	194	0.4392	0.165
Sugg, 2018[352]	Adults	Not-CBT-Based	Individual	CAU	Diagnosis	Low	Europe	65	0.8805	0.307

Study	Target group	Intervention	Format	Control	Diagnosis	Risk of bias	Region	N	Hedges´ g	SE
Swartz, 2008[353]	Other Target Groups	Not-CBT-Based	Individual	CAU	Diagnosis	Some Concern & High	North America	41	0.8482	0.330
Szumska, 2020[354]	Adults	CBT-Based	Group	WL	Diagnosis	Some Concern	Europe	20	1.4200	0.510
Takagaki, 2016[355]	Student Population	CBT-Based	Group	CAU	Subclinical Depression	Low	East Asia	118	0.8900	0.190
Talbot, 2011[356]	Other Target Groups	Not-CBT-Based	Individual	CAU	Diagnosis	Some Concern	North America	53	0.3050	0.280
Taylor, 1977[357]	Student Population	CBT-Based	Individual	WL	Cut-Off Score	Some Concern	North America	14	1.4894	0.632
Taylor, 2009[358]	General Medical	CBT-Based	Individual	WL	Cut-Off Score	Some Concern	North America	42	1.5988	0.447
Teasdale, 1984[359]	Adults	CBT-Based	Individual	CAU	Diagnosis	Some Concern	Europe	34	1.4600	0.470
Teichman, 1995[360]	Adults	CBT-Based	Individual & Other Formats	WL	Diagnosis	Some Concern	Other Region	30	0.8175	0.532
Teri, 1997[361]	General Medical	CBT-Based	Other Formats	CAU & WL	Diagnosis	Some Concern	North America	42	0.9697	0.448
Thomas, 2019[362]	General Medical	CBT-Based	Individual	CAU	Cut-Off Score	Low	Europe	30	0.2104	0.390
Titov, 2010[363]	Adults	CBT-Based	Guided Self-Help	WL	Diagnosis	Some Concern	Australia	85	1.0602	0.314
Titov, 2015a[364]	Older Adults	CBT-Based	Guided Self-Help	WL	Cut-Off Score	Low	Australia	42	2.1707	0.500
Tobin, 2017[365]	General Medical	CBT-Based	Other Formats	Other	Cut-Off Score	Low	North America	315	0.0500	0.110
Tomasino, 2017[366]	Older Adults	CBT-Based	Guided Self-Help	WL	Cut-Off Score	Some Concern	North America	26	0.4689	0.410
Tong, 2019[367]	Adults	CBT-Based	Group	WL	Diagnosis	Some Concern	East Asia	86	0.2725	0.315
Tovote, 2014[368]	General Medical	CBT-Based	Individual	WL	Cut-Off Score	Some Concern	Europe	63	0.6609	0.373
Town, 2017[369]	Adults	Not-CBT-Based	Individual	CAU	Diagnosis	Low	North America	60	1.1820	0.482

Plessen CY, et al. BMJ Ment Health 2023; 26:e300626. doi: 10.1136/bmjment-2022-300626

Study	Target group	Intervention	Format	Control	Diagnosis	Risk of bias	Region	N	Hedges´ g	SE
Trevillion, 2020[370]	Perinatal Depression	CBT-Based	Other Formats	CAU	Diagnosis	Low	Europe	51	0.3220	0.300
Tulbure, 2018[371]	Adults	CBT-Based	Guided Self-Help	WL	Diagnosis	Some Concern	Europe	53	0.8830	0.330
Turner, 1979[372]	Adults	CBT-Based	Individual	Other	Cut-Off Score	Some Concern	North America	34	0.0419	0.520
Turner, 2013[373]	General Medical	CBT-Based	Group	Other	Cut-Off Score	Some Concern	Australia	44	-0.1217	0.335
Tyson, 1987[374]	Adults	Not-CBT-Based	Group	Other	Cut-Off Score	Some Concern	North America	22	-0.1367	0.430
Unlu-Ince, 2013[375]	Other Target Groups	CBT-Based	Guided Self-Help	WL	Cut-Off Score	Some Concern	Europe	98	0.6925	0.323
Van Bastelaar, 2011[376]	General Medical	CBT-Based	Guided Self-Help	WL	Cut-Off Score	Low	Europe	250	0.4581	0.155
Van Lieshout, 2021[377]	Perinatal Depression	CBT-Based	Other Formats	WL	Cut-Off Score	Low	North America	403	0.510	0.100
Van Schaik, 2006[378]	Older Adults	Not-CBT-Based	Individual	CAU	Diagnosis	Some Concern	Europe	131	0.1067	0.194
Vazquez-Gonzalez, 2013[379]	Other Target Groups	CBT-Based	Group	CAU	Cut-Off Score	Some Concern	Europe	176	1.6569	0.205
Vazquez, 2017[380]	Other Target Groups	CBT-Based	Other Formats	CAU	Subclinical Depression	Low	Europe	40	2.0836	0.395
Vazquez, 2020[381]	Other Target Groups	CBT-Based	Other Formats	CAU	Subclinical Depression	Low	Europe	142	1.0505	0.333
Verduyn, 2003[382]	Other Target Groups	CBT-Based & Not-CBT-Based	Group	CAU	Cut-Off Score	Some Concern	Europe	42	0.1656	0.340
Vernmark, 2010[383]	Adults	CBT-Based	Guided Self-Help	WL	Diagnosis	Low	Europe	54	0.8340	0.346
Vigod, 2021[384]	Perinatal Depression	Not-CBT-Based	Group	WL	Cut-Off Score	Some Concern	North America	77	0.150	0.230
Vitriol, 2009[385]	Other Target Groups	Not-CBT-Based	Individual	CAU	Diagnosis	Some Concern	Other Region	81	0.5599	0.407
Warmerdam, 2008[386]	Adults	CBT-Based	Guided Self-Help	WL	Cut-Off Score	Low	Europe	175	0.4850	0.150

Study	Target group	Intervention	Format	Control	Diagnosis	Risk of bias	Region	N	Hedges´ g	SE
Watkins, 2012[387]	Adults	Not-CBT-Based	Other Formats	CAU & Other	Diagnosis	Low	Europe	68	0.4818	0.248
Watt, 2000[388]	Older Adults	Not-CBT-Based	Group	Other	Cut-Off Score	Some Concern	North America	18	1.5514	0.570
Weissman, 1979[389]	Adults	Not-CBT-Based	Individual	Other	Diagnosis	Some Concern	North America	34	1.1622	0.520
Westerhof, 2019[390]	Adults	Not-CBT-Based	Guided Self-Help & Other Formats	WL	Cut-Off Score	Low	Europe	26	-0.0450	0.400
Wickberg, 1996[391]	Perinatal Depression	Not-CBT-Based	Individual	CAU	Diagnosis	Some Concern	Europe	30	1.3300	0.480
Wiersma, 2014[392]	Adults	Not-CBT-Based	Individual	CAU	Diagnosis	Low	Europe	126	0.4740	0.220
Wiklund, 2010[393]	Perinatal Depression	CBT-Based	Individual	CAU	Cut-Off Score	Some Concern	Europe	66	0.8200	0.300
Williams, 2000[394]	Older Adults	CBT-Based	Individual	Other	Diagnosis	Low	North America	151	0.0691	0.187
Williams, 2013a[395]	Adults	CBT-Based	Guided Self-Help	WL	Diagnosis	Low	Australia	42	0.8822	0.338
Williams, 2013b[396]	Adults	CBT-Based	Guided Self-Help	CAU	Cut-Off Score	Some Concern	Europe	242	0.3032	0.145
Williams, 2018[397]	Adults	CBT-Based	Group	WL	Cut-Off Score	Low	Europe	104	0.6600	0.200
Wilson, 1983[398]	Adults	CBT-Based	Individual	WL	Cut-Off Score	Some Concern & High	Australia	17	1.9171	0.608
Wollersheim, 1991[399]	Adults	CBT-Based & Not-CBT-Based	Group & Guided Self-Help	WL	Diagnosis	Some Concern	North America	16	0.2389	0.617
Wong, 2008a[400]	Adults	CBT-Based	Group	WL	Cut-Off Score	Some Concern	East Asia	322	0.7400	0.120
Wong, 2008b[401]	Adults	CBT-Based	Group	WL	Diagnosis	Some Concern	East Asia	88	0.7600	0.220
Wong, 2018[402]	Adults	CBT-Based	Group	CAU	Subclinical Depression	Low	East Asia	231	0.1900	0.130
Wright, 2005[403]	Adults	CBT-Based	Individual & Guided Self-Help	WL	Diagnosis	Some Concern	North America	NA	1.1017	0.395
Wuthrich, 2013[404]	Older Adults	CBT-Based	Group	WL	Diagnosis	Some Concern	Australia	53	0.8377	0.330

Study	Target group	Intervention	Format	Control	Diagnosis	Risk of bias	Region	N	Hedges´ g	SE
Wuthrich, 2016[405]	Older Adults	CBT-Based	Group	Other	Diagnosis	Low	Australia	144	0.5676	0.200
Xie, 2019[406]	Older Adults	CBT-Based	Group	CAU	Cut-Off Score	Some Concern	East Asia	73	0.6800	0.240
Yang, 2018[407]	Student Population	Not-CBT-Based	Group	CAU	Diagnosis	Some Concern	East Asia	67	0.8000	0.250
Yeung, 2017[408]	Adults	CBT-Based	Guided Self-Help	WL	Cut-Off Score	Some Concern	East Asia	NA	0.6100	0.260
Yuan, 2020[409]	Older Adults	CBT-Based	Individual	CAU	Cut-Off Score	Low	East Asia	49	0.1740	0.380
Zemastani, 2020[410]	General Medical	CBT-Based	Group	Other	Diagnosis	Some Concern	Other Region	52	2.6200	0.380
Zemestani, 2016[411]	Student Population	CBT-Based	Group	WL	Diagnosis	Some Concern	Other Region	30	0.6100	0.030
Zemestani, 2019[412]	Perinatal Depression	CBT-Based	Group	CAU	Diagnosis	Low	Other Region	38	1.1800	0.120
Zhao, 2019[413]	Perinatal Depression	Not-CBT-Based	Group	CAU	Subclinical Depression	Some Concern	East Asia	245	0.4327	0.203
Zhao, 2021[414]	Perinatal Depression	Not-CBT-Based	Individual	CAU	Cut-Off Score	Some Concern	East Asia	182	0.310	0.150
Zu, 2014[415]	Adults	CBT-Based	Individual	CAU	Diagnosis	Some Concern & High	East Asia	42	0.2501	0.357

Notes. Control: WL = Wait list; CAU = Care as Usual. Intervention: CBT = Cognitive Behavioral Therapy. Diagnosis: Diagnosis = Diagnosis by a mental health professional; Cut-Off Score = Diagnosis based on scoring above a critical threshold on a self-reported questionnaire.

Plessen CY, *et al. BMJ Ment Health* 2023; 26:e300626. doi: 10.1136/bmjment-2022-300626

References of included primary studies

1. Aagaard J, Foldager L, Makki A, Hansen V, M¸ller-Nielsen K. The efficacy of psychoeducation on recurrent depression: a randomized trial with a 2-year follow-up. *Nord J Psychiatry*. 2017;71(3):223-229. doi:10.1080/08039488.2016.1266385

2. Abas M, Nyamayaro P, Bere T, et al. Feasibility and acceptability of a task-shifted intervention to enhance adherence to HIV medication and improve depression in people living with HIV in Zimbabwe, a low income country in sub-Saharan Africa. *AIDS Behav*. 2018;22(1):86-101. doi:10.1007/s10461-016-1659-4

3. Ahmadpanah M, Paghale SJ, Bakhtyari A, et al. Effects of psychotherapy in combination with pharmacotherapy, when compared to pharmacotherapy only on blood pressure, depression, and anxiety in female patients with hypertension. *J Health Psychol*. 2016;21(7):1216-1227. doi:10.1177/1359105314550350

4. Alexopoulos G, Raue P, McCulloch C, et al. Clinical Case Management versus Case Management with Problem-Solving Therapy in Low-Income, Disabled Elders with Major Depression: A Randomized Clinical Trial. *Am J Geriatr Psychiatry Off J Am Assoc Geriatr Psychiatry*. 2016;24(1):50-59. doi:10.1016/j.jagp.2015.02.007

5. Afonso R, Bueno B. Efectos de un programa de reminiscencia sobre la sintomatología depresiva en una muestra de población mayor portuguesa. *Rev Esp Geriatría Gerontol*. 2009;44(6):317-322.

6. Alhusen JL, Hayat MJ, Borg L. A pilot study of a group-based perinatal depression intervention on reducing depressive symptoms and improving maternal-fetal attachment and maternal sensitivity. *Arch Womens Ment Health*. 2021;24(1):145-154. doi:10.1007/s00737-020-01032-0

7. Allart-Van Dam E, Hosman CMH, Hoogduin CAL, Schaap CPDR. The coping with depression course: Short-term outcomes and mediating effects of a randomized controlled trial in the treatment of subclinical depression. *Behav Ther*. 2003;34(3):381-396. doi:10.1016/S0005-7894(03)80007-2

8. Amani B, Merza D, Savoy C, et al. Peer-Delivered Cognitive-Behavioral Therapy for Postpartum Depression: A Randomized Controlled Trial. *J Clin Psychiatry*. 2021;83(1). doi:10.4088/JCP.21m13928

9. Ammerman RT, Putnam FW, Altaye M, Stevens J, Teeters AR, Van Ginkel JB. A clinical trial of in-home CBT for depressed mothers in home visitation. *Behav Ther*. 2013;44(3):359-372. doi:10.1016/j.beth.2013.01.002

10. Andersson G, Bergstrˆm J, Holl‰ndare F, Carlbring P, Kaldo V, Ekselius L. Internet-based self-help for depression: randomised controlled trial. *Br J Psychiatry*. 2005;187:456-461. doi:10.1192/bjp.187.5.456

11. Araya R, Menezes PR, Claro HG, et al. Effect of a Digital Intervention on Depressive Symptoms in Patients With Comorbid Hypertension or Diabetes in Brazil and Peru: Two Randomized Clinical Trials. *Jama*. 2021;325(18):1852-1862. doi:10.1001/jama.2021.4348

12. Arean PA, Perri MG, Nezu AM, Schein RL, Christopher F, Joseph TX. Comparative effectiveness of social problem-solving therapy and reminiscence therapy as treatments for depression in older adults. *J Consult Clin Psychol*. 1993;61(6):1003-1010. doi:10.1037//0022-006x.61.6.1003

13. Arjadi R, Nauta MH, Scholte WF, et al. Internet-based behavioural activation with lay counsellor support versus online minimal psychoeducation without support for treatment of depression: a randomised controlled trial in Indonesia. *Lancet Psychiatry*. 2018;5(9):707-716. doi:10.1016/S2215-0366(18)30223-2

14. Ayen I, Hautzinger M. Cognitive behavior therapy for depression in menopausal women. A controlled, randomized treatment study. *Z Klin Psychol Psychother*. 2004;33(4):290-299. doi:10.1026/1616-3443.33.4.290

15. Barber JP, Barrett MS, Gallop R, Rynn MA, Rickels K. Short-term dynamic psychotherapy versus pharmacotherapy for major depressive disorder: a randomized, placebo-controlled trial. *J Clin Psychiatry*. 2012;73(1):66-73. doi:10.4088/JCP.11m06831

16. Barnhofer T, Crane C, Hargus E, Amarasinghe M, Winder R, Williams JM. Mindfulness-based cognitive therapy as a treatment for chronic depression: A preliminary study. *Behav Res Ther*. 2009;47(5):366-373. doi:10.1016/j.brat.2009.01.019

17. Barrett JE, Williams Jr JW, Oxman TE, et al. Treatment of dysthymia and minor depression in primary care: A ramdomized trial in patients aged 18 to 59 years. *J Fam Pract*. 2001;50(5):405-412.

18. Baumeister H, Paganini S, Sander LB, et al. Effectiveness of a Guided Internet- and Mobile-Based Intervention for Patients with Chronic Back Pain and Depression (WARD-BP): A Multicenter, Pragmatic Randomized Controlled Trial. *Psychother Psychosom*. 2021;90(4):255-268. doi:10.1159/000511881

19. Baumgartner C, Schaub MP, Wenger A, et al. “Take Care of You” - Efficacy of integrated, minimal-guidance, internet-based self-help for reducing co-occurring alcohol misuse and depression symptoms in adults: Results of a three-arm randomized controlled trial. *Drug Alcohol Depend*. 2021;225:108806. doi:10.1016/j.drugalcdep.2021.108806

20. Beach SRH, O’Leary KD. Treating depression in the context of marital discord: Outcome and predictors of response of marital therapy versus cognitive therapy. *Behav Ther*. 1992;23(4):507-528. doi:10.1016/S0005-7894(05)80219-9

21. Bedard M, Felteau M, Marshall S, et al. Mindfulness-based cognitive therapy reduces symptoms of depression in people with a traumatic brain injury: Results from a randomized controlled trial. *J Head Trauma Rehabil*. 2014;29(4):E13-E22. doi:10.1097/HTR.0b013e3182a615a0

22. Beeber LS, Holditch-Davis D, Perreira K, et al. Short-term in-home intervention reduces depressive symptoms in Early Head Start Latina mothers of infants and toddlers. *Res Nurs Health*. 2010;33(1):60-76. doi:10.1002/nur.20363

23. Bendig E, Bauereiß N, Buntrock C, Habibović M, Ebert DD, Baumeister H. Lessons learned from an attempted randomized-controlled feasibility trial on “WIDeCAD” - An internet-based depression treatment for people living with coronary artery disease (CAD). *Internet Interv*. 2021;24. doi:10.1016/j.invent.2021.100375

24. Berger T, H‰mmerli K, Gubser N, Andersson G, Caspar F. Internet-based treatment of depression: a randomized controlled trial comparing guided with unguided self-help. *Cogn Behav Ther*. 2011;40(4):251-266. doi:10.1080/16506073.2011.616531

25. Beutel ME, Weissflog G, Leuteritz K, et al. Efficacy of short-term psychodynamic psychotherapy (STPP) with depressed breast cancer patients: Results of a randomized controlled multicenter trial. *Ann Oncol*. 2014;25(2):378-384. doi:10.1093/annonc/mdt526

26. Boele FW, Klein M, Verdonck-de Leeuw IM, et al. Internet-based guided self-help for glioma patients with depressive symptoms: a randomized controlled trial. *J Neurooncol*. 2018;137(1):191-203. doi:10.1007/s11060-017-2712-5

27. Boeschoten RE, Dekker J, Uitdehaag BMJ, et al. Internet-based treatment for depression in multiple sclerosis: A randomized controlled trial. *Mult Scler*. 2017;23(8):1112-1122. doi:10.1177/1352458516671820

28. Bohlmeijer ET, Fledderus M, Rokx TA, Pieterse ME. Efficacy of an early intervention based on acceptance and commitment therapy for adults with depressive symptomatology: Evaluation in a randomized controlled trial. *Behav Res Ther*. 2011;49(1):62-67. doi:10.1016/j.brat.2010.10.003

29. Bolton P, Bass J, Neugebauer R, et al. Group interpersonal psychotherapy for depression in rural Uganda: a randomized controlled trial. *Jama*. 2003;289(23):3117-3124. doi:10.1001/jama.289.23.3117

30. Bower JE, Partridge AH, Wolff AC, et al. Targeting Depressive Symptoms in Younger Breast Cancer Survivors: The Pathways to Wellness Randomized Controlled Trial of Mindfulness Meditation and Survivorship Education. *J Clin Oncol*. 2021;39(31):3473-3484. doi:10.1200/jco.21.00279

31. Bowman D, Scogin F, Lyrene B. The efficacy of self-examination therapy and cognitive bibliotherapy in the treatment of mild to moderate depression. *Psychother Res*. 1995;5(2):131-140. doi:10.1080/10503309512331331256

32. Braun L, Titzler I, Terhorst Y, et al. Effectiveness of guided internet-based interventions in the indicated prevention of depression in green professions (PROD-A): Results of a pragmatic randomized controlled trial. *J Affect Disord*. 2021;278:658-671. doi:10.1016/j.jad.2020.09.066

33. Brown RA, Lewinsohn PM. A psychoeducational approach to the treatment of depression: comparison of group, individual, and minimal contact procedures. *J Consult Clin Psychol*. 1984;52(5):774. doi:10.1037//0022-006x.52.5.774

34. Buhrman M, Syk M, Burvall O, Hartig T, Gordh T, Andersson G. Individualized guided internet-delivered cognitive-behavior therapy for chronic pain patients with comorbid depression and anxiety: A randomized controlled trial. *Clin J Pain*. 2015;31(6):504-516. doi:10.1097/AJP.0000000000000176

35. Buntrock C, Ebert D, Lehr D, et al. Effectiveness of a Web-Based Cognitive Behavioural Intervention for Subthreshold Depression: Pragmatic Randomised Controlled Trial. *Psychother Psychosom*. 2015;84(6):348-358. doi:10.1159/000438673

36. Burmaoğlu GE. Comparison of the effect of mindfulness based on stress reduction and dialectical behavior therapy on reducing depression in mothers with handicapped children in rehabilitation centers. *Pak J Med Health Sci*. 2021;15(3):885-890.

37. Burns A, Banerjee S, Morris J, et al. Treatment and prevention of depression after surgery for hip fracture in older people: randomized, controlled trials. *J Am Geriatr Soc*. 2007;55(1):75-80. doi:10.1111/j.1532-5415.2007.01016.x

38. Burns A, O'Mahen H, Baxter H, et al. A pilot randomised controlled trial of cognitive behavioural therapy for antenatal depression. *BMC Psychiatry*. 2013;13:33. doi:10.1186/1471-244x-13-33

39. Carlbring P, Hagglund M, Luthstrom A, et al. Internet-based behavioral activation and acceptance-based treatment for depression: a randomized controlled trial. *J Affect Disord*. 2013;148(2-3):331-337. doi:10.1016/j.jad.2012.12.020

40. Carr A, Finnegan L, Griffin E, Cotter P, Hyland A. A Randomized Controlled Trial of the Say Yes to Life (SYTL) Positive Psychology Group Psychotherapy Program for Depression: An Interim Report. *J Contemp Psychother*. 2017;47(3):153-161. doi:10.1007/s10879-016-9343-6

41. Carta MG, Petretto D, Adamo S, et al. Counseling in primary care improves depression and quality of life. *Clin Pract Epidemiol Ment Health*. 2012;8:152-157. doi:10.2174/1745017901208010152

42. Casanas R, Catalan R, del Val JL, Real J, Valero S, Casas M. Effectiveness of a psycho-educational group program for major depression in primary care: a randomized controlled trial. *BMC Psychiatry*. 2012;12:230. doi:10.1186/1471-244x-12-230

43. Castonguay LG, Schut AJ, Aikens DE, et al. Integrative Cognitive Therapy for Depression: A Preliminary Investigation. *J Psychother Integr*. 2004;14(1):4-20. doi:10.1037/1053-0479.14.1.4

44. Chan AS, Wong QY, Sze SL, Kwong PP, Han YM, Cheung MC. A Chinese Chan-based mind-body intervention for patients with depression. *J Affect Disord*. 2012;142(1-3):283-289. doi:10.1016/j.jad.2012.05.018

45. Chan MF, Ng SE, Tien A, Man Ho RC, Thayala J. A randomised controlled study to explore the effect of life story review on depression in older Chinese in Singapore. *Health Soc Care Community*. 2013;21(5):545-553. doi:10.1111/hsc.12043

46. Chen CH, Tseng YF, Chou FH, Wang SY. Effects of support group intervention in postnatally distressed women. A controlled study in Taiwan. *J Psychosom Res*. 2000;49(6):395-399. doi:10.1016/s0022-3999(00)00180-x

47. Chesney MA, Chambers DB, Taylor JM, Johnson LM, Folkman S. Coping effectiveness training for men living with HIV: results from a randomized clinical trial testing a group-based intervention. *Psychosom Med*. 2003;65(6):1038-1046. doi:10.1097/01.psy.0000097344.78697.ed

48. Chiang KJ, Chen TH, Hsieh HT, Tsai JC, Ou KL, Chou KR. One-Year Follow-Up of the Effectiveness of Cognitive Behavioral Group Therapy for Patients' Depression: A Randomized, Single-Blinded, Controlled Study. *Sci World J*. 2015;2015:373149. doi:10.1155/2015/373149

49. Cho HJ, Kwon JH, Lee JJ. Antenatal cognitive-behavioral therapy for prevention of postpartum depression: a pilot study. *Yonsei Med J*. 2008;49(4):553-562. doi:10.3349/ymj.2008.49.4.553

50. Choi I, Zou J, Titov N, et al. Culturally attuned Internet treatment for depression amongst Chinese Australians: a randomised controlled trial. *J Affect Disord*. 2012;136(3):459-468. doi:10.1016/j.jad.2011.11.003

51. Choi NG, Hegel MT, Marti CN, Marinucci ML, Sirrianni L, Bruce ML. Telehealth problem-solving therapy for depressed low-income homebound older adults. *Am J Geriatr Psychiatry*. 2014;22(3):263-271. doi:10.1016/j.jagp.2013.01.037

52. Choi NG, Marti CN, Wilson NL, et al. Effect of Telehealth Treatment by Lay Counselors vs by Clinicians on Depressive Symptoms Among Older Adults Who Are Homebound: A Randomized Clinical Trial. *JAMA Netw Open*. 2020;3(8):e2015648. doi:10.1001/jamanetworkopen.2020.15648

53. Chowdhary N, Anand A, Dimidjian S, et al. The healthy activity program lay counsellor delivered treatment for severe depression in India: Systematic development and randomised evaluation. *Br J Psychiatry*. 2016;208(4):381-388. doi:10.1192/bjp.bp.114.161075

54. Choy J, Lou V. Effectiveness of the Modified Instrumental Reminiscence Intervention on Psychological Well-Being Among Community-Dwelling Chinese Older Adults: A Randomized Controlled Trial. *Am J Geriatr Psychiatry Off J Am Assoc Geriatr Psychiatry*. 2016;24(1):60-69. doi:10.1016/j.jagp.2015.05.008

55. Clark R, Tluczek A, Wenzel A. Psychotherapy for Postpartum Depression: A Preliminary Report. *Am J Orthopsychiatry*. 2003;73(4):441-454. doi:10.1037/0002-9432.73.4.441

56. Clark R, Tluczek A, Brown R. A mother–infant therapy group model for postpartum depression. *Infant Ment Health J*. 2008;29(5):514-536. doi:10.1002/imhj.20189

57. Cohen S, O'Leary KD, Foran H. A randomized clinical trial of a brief, problem-focused couple therapy for depression. *Behav Ther*. 2010;41(4):433-446. doi:10.1016/j.beth.2009.11.004

58. Cooper PJ, Murray L, Wilson A, Romaniuk H. Controlled trial of the short- and long-term effect of psychological treatment of post-partum depression. I. Impact on maternal mood. *Br J Psychiatry*. 2003;182:412-419. doi:10.1192/bjp.182.5.412

59. Cramer H, Salisbury C, Conrad J, Eldred J, Araya R. Group cognitive behavioural therapy for women with depression: pilot and feasibility study for a randomised controlled trial using mixed methods. *BMC Psychiatry*. 2011;11:82. doi:10.1186/1471-244x-11-82

60. D'Elia A, Bawor M, Dennis BB, et al. Feasibility of behavioral activation group therapy in reducing depressive symptoms and improving quality of life in patients with depression: The BRAVE pilot trial. *Pilot Feasibility Stud*. 2020;6(1). doi:10.1186/s40814-020-00596-z

61. Davoudi M, Taheri AA, Foroughi AA, Ahmadi SM, Heshmati K. Effectiveness of acceptance and commitment therapy (ACT) on depression and sleep quality in painful diabetic neuropathy: a randomized clinical trial. *J Diabetes Metab Disord*. Published online 2020. doi:10.1007/s40200-020-00609-x

62. De Groot M, Shubrook JH, Hornsby WG, et al. Program ACTIVE II: Outcomes from a randomized, multistate community-based depression treatment for rural and urban adults with type 2 diabetes. *Diabetes Care*. 2019;42(7):1185-1193. doi:10.2337/dc18-2400

63. De Jong M, Peeters F, Gard T, et al. A randomized controlled pilot study on mindfulness-based cognitive therapy for unipolar depression in patients with chronic pain. *J Clin Psychiatry*. 2018;79(1):26-34. doi:10.4088/JCP.15m10160

64. Dekker RL, Moser DK, Peden AR, Lennie TA. Cognitive therapy improves three-month outcomes in hospitalized patients with heart failure. *J Card Fail*. 2012;18(1):10-20. doi:10.1016/j.cardfail.2011.09.008

65. Dennis CL, Grigoriadis S, Zupancic J, Kiss A, Ravitz P. Telephone-based nurse-delivered interpersonal psychotherapy for postpartum depression: nationwide randomised controlled trial. *Br J Psychiatry*. 2020;216(4):189-196. doi:10.1192/bjp.2019.275

66. DeRubeis RJ, Hollon SD, Amsterdam JD, et al. Cognitive therapy vs medications in the treatment of moderate to severe depression. *Arch Gen Psychiatry*. 2005;62(4):409-416. doi:10.1001/archpsyc.62.4.409

67. Desautels C, Savard J, Ivers H, Savard MH, Caplette-Gingras A. Treatment of Depressive Symptoms in Patients with Breast Cancer: A Randomized Controlled Trial Comparing Cognitive Therapy and Bright Light Therapy. *Health Psychol*. 2017;37(1):1-13. doi:10.1037/hea0000539

68. Dimidjian S, Hollon SD, Dobson KS, et al. Randomized trial of behavioral activation, cognitive therapy, and antidepressant medication in the acute treatment of adults with major depression. *J Consult Clin Psychol*. 2006;74(4):658-670. doi:10.1037/0022-006x.74.4.658

69. Dimidjian S, Goodman S, Sherwood N, et al. A Pragmatic Randomized Clinical Trial of Behavioral Activation for Depressed Pregnant Women. *J Consult Clin Psychol*. 2017;85(1):26-36. doi:10.1037/ccp0000151

70. Dindo L, Recober A, Marchman JN, Turvey C, O'Hara MW. One-day behavioral treatment for patients with comorbid depression and migraine: A pilot study. *Behav Res Ther*. 2012;50(9):537-543. doi:10.1016/j.brat.2012.05.007

71. Dindo LN, Recober A, Calarge CA, et al. One-Day Acceptance and Commitment Therapy Compared to Support for Depressed Migraine Patients: a Randomized Clinical Trial. *Neurotherapeutics*. 2019;17(2):743-753. doi:10.1007/s13311-019-00818-0

72. Dobkin RD, Menza M, Allen LA, et al. Cognitive-behavioral therapy for depression in Parkinson's disease: a randomized, controlled trial. *Am J Psychiatry*. 2011;168(10):1066-1074. doi:10.1176/appi.ajp.2011.10111669

73. Dobkin RD, Mann SL, Gara MA, Interian A, Rodriguez KM, Menza M. Telephone-based cognitive behavioral therapy for depression in Parkinson disease: A randomized controlled trial. *Neurology*. 2020;94(16):e1764-e1773. doi:10.1212/wnl.0000000000009292

74. Dobkin RD, Mann SL, Weintraub D, et al. Innovating Parkinson's Care: A Randomized Controlled Trial of Telemedicine Depression Treatment. *Mov Disord*. 2021;36(11):2549-2558. doi:10.1002/mds.28548

75. Doering LV, Chen B, Cross Bodan R, Magsarili MC, Nyamathi A, Irwin MR. Early cognitive behavioral therapy for depression after cardiac surgery. *J Cardiovasc Nurs*. 2013;28(4):370-379. doi:10.1097/JCN.0b013e31824d967d

76. Dong X, Sun G, Zhan J, et al. Telephone-based reminiscence therapy for colorectal cancer patients undergoing postoperative chemotherapy complicated with depression: a three-arm randomised controlled trial. *Support Care Cancer Off J Multinatl Assoc Support Care Cancer*. 2019;27(8):2761-2769. doi:10.1007/s00520-018-4566-6

77. Duarte PS, Miyazaki MC, Blay SL, Sesso R. Cognitive-behavioral group therapy is an effective treatment for major depression in hemodialysis patients. *Kidney Int*. 2009;76(4):414-421. doi:10.1038/ki.2009.156

78. Dwight-Johnson M, Aisenberg E, Golinelli D, Hong S, O'Brien M, Ludman E. Telephone-Based Cognitive-Behavioral Therapy for Latino Patients Living in Rural Areas: A Randomized Pilot Study. *Psychiatr Serv*. 2011;62(8):936-942. doi:10.1176/ps.62.8.pss6208_0936

79. Ebert DD, Lehr D, Boß L, et al. Efficacy of an internet-based problem-solving training for teachers: results of a randomized controlled trial. *Scand J Work Environ Health*. 2014;40(6):582-596. doi:10.5271/sjweh.3449

80. Ebert DD, Buntrock C, Lehr D, et al. Effectiveness of Web- and Mobile-Based Treatment of Subthreshold Depression With Adherence-Focused Guidance: A Single-Blind Randomized Controlled Trial. *Behav Ther*. 2018;49(1):71-83. doi:10.1016/j.beth.2017.05.004

81. Ede MO, Igbo JN, Eseadi C, et al. Effect of group cognitive behavioural therapy on depressive symptoms in a sample of college adolescents in Nigeria. *J Ration-Emotive Cogn-Behav Ther*. 2020;38(3):306-318. doi:10.1007/s10942-019-00327-1

82. Ekers D, Richards D, McMillan D, Bland JM, Gilbody S. Behavioural activation delivered by the non-specialist: phase II randomised controlled trial. *Br J Psychiatry*. 2011;198(1):66-72. doi:10.1192/bjp.bp.110.079111

83. Ekkers W, Korrelboom K, Huijbrechts I, Smits N, Cuijpers P, Gaag M. Competitive Memory Training for treating depression and rumination in depressed older adults: a randomized controlled trial. *Behav Res Ther*. 2011;49(10):588-596. doi:10.1016/j.brat.2011.05.010

84. Elkin I, Shea MT, Watkins JT, et al. National Institute of Mental Health Treatment of Depression Collaborative Research Program. General effectiveness of treatments. *Arch Gen Psychiatry*. 1989;46(11):971-982; discussion 983. doi:10.1001/archpsyc.1989.01810110013002

85. Embling S. The effectiveness of cognitive behavioural therapy in depression. *Nurs Stand*. 2002;17(14-15):33-41. doi:10.7748/ns2002.12.17.14.33.c3318

86. Eseadi C, Obidoa MA, Ogbuabor SE, Ikechukwu-Ilomuanya AB. Effects of Group-Focused Cognitive-Behavioral Coaching Program on Depressive Symptoms in a Sample of Inmates in a Nigerian Prison. *Int J Offender Ther Comp Criminol*. 2018;62(6):1589-1602. doi:10.1177/0306624X16687046

87. Euteneuer F, Dannehl K, Del Rey A, Engler H, Schedlowski M, Rief W. Immunological effects of behavioral activation with exercise in major depression: An exploratory randomized controlled trial. *Transl Psychiatry*. 2017;7(5):e1132. doi:10.1038/tp.2017.76

88. Evans RL, Connis RT. Comparison of brief group therapies for depressed cancer patients receiving radiation treatment. *Public Health Rep*. 1995;110(3):306-311.

89. Fann JR, Bombardier CH, Vannoy S, et al. Telephone and In-Person Cognitive Behavioral Therapy for Major Depression after Traumatic Brain Injury: A Randomized Controlled Trial. *J Neurotrauma*. 2015;32(1):45-57. doi:10.1089/neu.2014.3423

90. Faramarzi M, Alipor A, Esmaelzadeh S, Kheirkhah F, Poladi K, Pash H. Treatment of depression and anxiety in infertile women: cognitive behavioral therapy versus fluoxetine. *J Affect Disord*. 2008;108(1-2):159-164. doi:10.1016/j.jad.2007.09.002

91. Fledderus M, Bohlmeijer ET, Pieterse ME, Schreurs KM. Acceptance and commitment therapy as guided self-help for psychological distress and positive mental health: a randomized controlled trial. *Psychol Med*. 2012;42(3):485-495. doi:10.1017/s0033291711001206

92. Floyd M, Scogin F, McKendree-Smith NL, Floyd DL, Rokke PD. Cognitive therapy for depression: a comparison of individual psychotherapy and bibliotherapy for depressed older adults. *Behav Modif*. 2004;28(2):297-318. doi:10.1177/0145445503259284

93. Flygare AL, Engström I, Hasselgren M, et al. Internet-based CBT for patients with depressive disorders in primary and psychiatric care: Is it effective and does comorbidity affect outcome? *Internet Interv*. 2020;19. doi:10.1016/j.invent.2019.100303

94. Folke F, Parling T, Melin L. Acceptance and commitment therapy for depression: A preliminary randomized clinical trial for unemployed on long-term sick leave. *Cogn Behav Pract*. 2012;19(4):583-594. doi:10.1016/j.cbpra.2012.01.002

95. Fonagy P, Rost F, Carlyle JA, et al. Pragmatic randomized controlled trial of long-term psychoanalytic psychotherapy for treatment-resistant depression: The Tavistock Adult Depression Study (TADS). *World Psychiatry*. 2015;14(3):312-321. doi:10.1002/wps.20267

96. Forand NR, Barnett JG, Strunk DR, Hindiyeh MU, Feinberg JE, Keefe JR. Efficacy of Guided iCBT for Depression and Mediation of Change by Cognitive Skill Acquisition. *Behav Ther*. 2018;49(2):295-307. doi:10.1016/j.beth.2017.04.004

97. Forsell E, Bendix M, Holländare F, et al. Internet delivered cognitive behavior therapy for antenatal depression: A randomised controlled trial. *J Affect Disord*. 2017;221:56-64. doi:10.1016/j.jad.2017.06.013

98. Frangou E, Bertelli G, Love S, et al. OVPSYCH2: A randomized controlled trial of psychological support versus standard of care following chemotherapy for ovarian cancer. *Gynecol Oncol*. 2021;162(2):431-439. doi:10.1016/j.ygyno.2021.05.024

99. Freedland KE, Skala JA, Carney RM, et al. Treatment of depression after coronary artery bypass surgery: a randomized controlled trial. *Arch Gen Psychiatry*. 2009;66(4):387-396. doi:10.1001/archgenpsychiatry.2009.7

100. Freedland KE, Carney RM, Rich MW, Steinmeyer BC, Rubin EH. Cognitive behavior therapy for depression and self-care in heart failure patients a randomized clinical trial. *JAMA Intern Med*. 2015;175(11):1773-1782. doi:10.1001/jamainternmed.2015.5220

101. Fry P. Structured and unstructured reminiscence training and depression among the elderly. *Clin Gerontol*. 1983;1(3):15-37. doi:10.1300/J018v01n03_06

102. Fuhr DC, Weobong B, Lazarus A, et al. Delivering the Thinking Healthy Programme for perinatal depression through peers: An individually randomised controlled trial in India. *Lancet Psychiatry*. 2019;6(2):115-127. doi:10.1016/S2215-0366(18)30466-8

103. Funderburk JS, Pigeon WR, Shepardson RL, et al. Treating depressive symptoms among veterans in primary care: A multi-site RCT of brief behavioral activation. *J Affect Disord*. 2021;283:11-19. doi:10.1016/j.jad.2021.01.033

104. Furukawa TA, Horikoshi M, Kawakami N, et al. Telephone cognitive-behavioral therapy for subthreshold depression and presenteeism in workplace: a randomized controlled trial. *PLoS One*. 2012;7(4):e35330. doi:10.1371/journal.pone.0035330

105. García-Peña C, Vázquez-Estupiñan F, Avalos-Pérez F, Jiménez LVR, Sánchez-Garcia S, Juárez-Cedillo T. Clinical effectiveness of group cognitive-behavioural therapy for depressed older people in primary care: A randomised controlled trial. *Salud Ment*. 2015;38(1):33-39. doi:10.17711/SM.0185-3325.2015.004

106. Gawrysiak M, Nicholas C, Hopko DR. Behavioral activation for moderately depressed university students: Randomized controlled trial. *J Couns Psychol*. 2009;56(3):468-475. doi:10.1037/a0016383

107. Gellis ZD, McGinty J, Tierney L, Jordan C, Burton J, Misener E. Randomized controlled trial of problem-solving therapy for minor depression in home care. [References]. *Res Soc Work Pract*. 2008;18(6):596-606. doi:10.1177/1049731507309821

108. Gellis ZD, Bruce ML. Problem solving therapy for subthreshold depression in home healthcare patients with cardiovascular disease. *Am J Geriatr Psychiatry*. 2010;18(6):464-474. doi:10.1097/jgp.0b013e3181b21442

109. Geraedts AS, Kleiboer AM, Wiezer NM, van Mechelen W, Cuijpers P. Short-term effects of a web-based guided self-help intervention for employees with depressive symptoms: randomized controlled trial. *J Med Internet Res*. 2014;16(5):e121. doi:10.2196/jmir.3185

110. Ghorbani V, Zanjani Z, Omidi A, Sarvizadeh M. Efficacy of acceptance and commitment therapy (ACT) on depression, pain acceptance, and psychological flexibility in married women with breast cancer: a pre- and post-test clinical trial. *Trends Psychiatry Psychother*. 2021;43(2):126-133. doi:10.47626/2237-6089-2020-0022

111. Gibbons MB, Thompson SM, Scott K, et al. Supportive-expressive dynamic psychotherapy in the community mental health system: a pilot effectiveness trial for the treatment of depression. *Psychother Chic*. 2012;49(3):303-316. doi:10.1037/a0027694

112. Gitlin LN, Harris LF, McCoy MC, et al. A home-based intervention to reduce depressive symptoms and improve quality of life in older African Americans: a randomized trial. *Ann Intern Med*. 2013;159(4):243-252. doi:10.7326/0003-4819-159-4-201308200-00005

113. Goodman JH, Prager J, Goldstein R, Freeman M. Perinatal Dyadic Psychotherapy for postpartum depression: a randomized controlled pilot trial. *Arch Womens Ment Health*. 2015;18(3):493-506. doi:10.1007/s00737-014-0483-y

114. Greenberg J, Datta T, Shapero BG, Sevinc G, Mischoulon D, Lazar SW. Compassionate hearts protect against wandering minds: Self-compassion moderates the effect of mind-wandering on depression. *Spiritual Clin Pract*. 2018;5(3):155-169. doi:10.1037/scp0000168

115. Grote NK, Swartz HA, Geibel SL, Zuckoff A, Houck PR, Frank E. A randomized controlled trial of culturally relevant, brief interpersonal psychotherapy for perinatal depression. *Psychiatr Serv*. 2009;60(3):313-321. doi:10.1176/appi.ps.60.3.313

116. Hagen R, Hjemdal O, Solem S, et al. Metacognitive therapy for depression in adults: A waiting list randomized controlled trial with six months follow-up. *Front Psychol*. 2017;8:31. doi:10.3389/fpsyg.2017.00031

117. Hallford D, Mellor D. Autobiographical memory-based intervention for depressive symptoms in young adults: A randomized controlled trial of cognitive-reminiscence therapy. *Psychother Psychosom*. 2016;85(4):246-249. doi:10.1159/000444417

118. Hallgren M, Kraepelien M, Öjehagen A, et al. Physical exercise and internet-based cognitive-behavioural therapy in the treatment of depression: Randomised controlled trial. *Br J Psychiatry*. 2015;207(3):227-234. doi:10.1192/bjp.bp.114.160101

119. Hamamci Z. Integrating psychodrama and cognitive behavioral therapy to treat moderate depression. *Arts Psychother*. 2006;33(3):199-207. doi:10.1016/j.aip.2006.02.001

120. Hamdan-Mansour AM, Puskar K, Bandak AG. Effectiveness of cognitive-behavioral therapy on depressive symptomatology, stress and coping strategies among Jordanian university students. *Issues Ment Health Nurs*. 2009;30(3):188-196. doi:10.1080/01612840802694577

121. Han YMY, Sze SL, Wong QY, Chan AS. A mind-body lifestyle intervention enhances emotional control in patients with major depressive disorder: a randomized, controlled study. *Cogn Affect Behav Neurosci*. 2020;20(5):1056-1069. doi:10.3758/s13415-020-00819-z

122. Haringsma R, Engels G, Cuijpers P, Spinhoven P. Effectiveness of the Coping With Depression (CWD) course for older adults provided by the community-based mental health care system in the Netherlands: a randomized controlled field trial. *Int Psychogeriatr*. 2006;18(02):307-325. doi:10.1017/S104161020500253X

123. Harley R, Sprich S, Safren S, Jacobo M, Fava M. Adaptation of dialectical behavior therapy skills training group for treatment-resistant depression. *J Nerv Ment Dis*. 2008;196(2):136-143. doi:10.1097/NMD.0b013e318162aa3f

124. Harrer M, Apolinário-Hagen J, Fritsche L, et al. Effect of an internet- and app-based stress intervention compared to online psychoeducation in university students with depressive symptoms: Results of a randomized controlled trial. *Internet Interv*. 2021;24. doi:10.1016/j.invent.2021.100374

125. Hassiotis A, Serfaty M, Azam K, et al. Manualised Individual Cognitive Behavioural Therapy for mood disorders in people with mild to moderate intellectual disability: A feasibility randomised controlled trial. *J Affect Disord*. 2013;151(1):186-195. doi:10.1016/j.jad.2013.05.076

126. Hautzinger M, Welz S. Kognitive Verhaltenstherapie bei Depressionen im Alter: Ergebnisse einer kontrollierten Vergleichsstudie unter ambulanten Bedingungen an Depressionen mittleren Schweregrads. = Cognitive behavioral therapy for depressed older outpatients--A controlled, randomized trial. *Z Für Gerontol Geriatr*. 2004;37(6):427-435. doi:10.1007/s00391-004-0262-x

127. Hayman PM, Cope CS. Effects of assertion training on depression. *J Clin Psychol*. 1980;36(2):534-543. doi:10.1002/jclp.6120360226

128. He HL, Zhang M, Gu CZ, et al. Effect of Cognitive Behavioral Therapy on Improving the Cognitive Function in Major and Minor Depression. *J Nerv Ment Dis*. 2019;207(4):232-238. doi:10.1097/NMD.0000000000000954

129. Heckman TG, Sikkema KJ, Hansen N, et al. A randomized clinical trial of a coping improvement group intervention for HIV-infected older adults. *J Behav Med*. 2011;34(2):102-111. doi:10.1007/s10865-010-9292-6

130. Heckman TG, Heckman BD, Anderson T, et al. Supportive-expressive and coping group teletherapies for HIV-infected older adults: a randomized clinical trial. *AIDS Behav*. 2013;17(9):3034-3044. doi:10.1007/s10461-013-0441-0

131. Heckman TG, Heckman BD, Anderson T, et al. Tele-interpersonal psychotherapy acutely reduces depressive symptoms in depressed HIV-infected rural persons: A randomized clinical trial. *Behav Med*. 2017;43(4):285-295. doi:10.1080/08964289.2016.1160025

132. Hegerl U, Hautzinger M, Mergl R, et al. Effects of pharmacotherapy and psychotherapy in depressed primary-care patients: a randomized, controlled trial including a patients' choice arm. *Int J Neuropsychopharmacol*. 2010;13(1):31-44. doi:10.1017/S1461145709000224

133. Hemanny C, Carvalho C, Maia N, et al. Efficacy of trial-based cognitive therapy, behavioral activation and treatment as usual in the treatment of major depressive disorder: Preliminary findings from a randomized clinical trial. *CNS Spectr*. 2019;25(4):535-544. doi:10.1017/S1092852919001457

134. Hermanns N, Schmitt A, Gahr A, et al. The effect of a Diabetes-Specific Cognitive Behavioral Treatment Program (DIAMOS) for patients with diabetes and subclinical depression: results of a randomized controlled trial. *Diabetes Care*. 2015;38(4):551-560. doi:10.2337/dc14-1416

135. Herrmann-Lingen C, Beutel ME, Bosbach A, et al. A stepwise psychotherapy intervention for reducing risk in coronary artery disease (SPIRR-CAD): Results of an observer-blinded, multicenter, randomized trial in depressed patients with coronary artery disease. *Psychosom Med*. 2016;78(6):704-715. doi:10.1097/PSY.0000000000000332

136. Hoifodt RS, Lillevoll KR, Griffiths KM, et al. The clinical effectiveness of web-based cognitive behavioral therapy with face-to-face therapist support for depressed primary care patients: randomized controlled trial. *J Med Internet Res*. 2013;15(8):e153. doi:10.2196/jmir.2714

137. Holden JM, Sagovsky R, Cox JL. Counselling in a general practice setting: Controlled study of heath visitor intervention in treatment of postnatal depression. *Br Med J*. 1989;298(6668):223-226. doi:10.1136/bmj.298.6668.223

138. Honey KL, Bennett P, Morgan M. A brief psycho-educational group intervention for postnatal depression. *Br J Clin Psychol*. 2002;41(4):405-409. doi:10.1348/014466502760387515

139. Horrell L, Goldsmith KA, Tylee AT, et al. One-day cognitive–behavioural therapy self-confidence workshops for people with depression: Randomised controlled trial. *Br J Psychiatry*. 2014;204(3):222-233. doi:10.1192/bjp.bp.112.121855

140. Hou Y, Hu P, Zhang Y, et al. Cognitive behavioral therapy in combination with systemic family therapy improves mild to moderate postpartum depression. *Rev Bras Psiquiatr*. 2014;36(1):47-52. doi:10.1590/1516-4446-2013-1170

141. Hsiao FH, Lai YM, Chen YT, et al. Efficacy of psychotherapy on diurnal cortisol patterns and suicidal ideation in adjustment disorder with depressed mood. *Gen Hosp Psychiatry*. 2014;36(2):214-219. doi:10.1016/j.genhosppsych.2013.10.019

142. Huang CY, Lai HL, Chen CI, et al. Effects of motivational enhancement therapy plus cognitive behaviour therapy on depressive symptoms and health-related quality of life in adults with type II diabetes mellitus: A randomised controlled trial. *Qual Life Res Int J Qual Life Asp Treat Care Rehabil*. 2016;25(5):1275-1283. doi:10.1007/s11136-015-1165-6

143. Hum KM, Chan CJ, Gane J, Conway L, McAndrews MP, Smith ML. Do distance-delivery group interventions improve depression in people with epilepsy? *Epilepsy Behav*. 2019;98(Pt A):153-160. doi:10.1016/j.yebeh.2019.06.037

144. Hummel J, Weisbrod C, Boesch L, et al. AIDE–Acute Illness and Depression in Elderly Patients. Cognitive Behavioral Group Psychotherapy in Geriatric Patients With Comorbid Depression: A Randomized, Controlled Trial. *J Am Med Dir Assoc*. 2017;18(4):341-349. doi:10.1016/j.jamda.2016.10.009

145. Hunter SB, Watkins KE, Hepner KA, et al. Treating depression and substance use: a randomized controlled trial. *J Subst Abuse Treat*. 2012;43(2):137-151. doi:10.1016/j.jsat.2011.12.004

146. Husain N, Zulqernain F, Carter LA, et al. Treatment of maternal depression in urban slums of Karachi, Pakistan: a randomized controlled trial (RCT) of an integrated maternal psychological and early child development intervention. *Asian J Psychiatry*. 2017;29:63-70. doi:10.1016/j.ajp.2017.03.010

147. Husain N, Kiran T, Fatima B, et al. An integrated parenting intervention for maternal depression and child development in a low-resource setting: Cluster randomized controlled trial. *Depress Anxiety*. 2021;38(9):925-939. doi:10.1002/da.23169

148. Husain N, Kiran T, Shah S, et al. Efficacy of learning through play plus intervention to reduce maternal depression in women with malnourished children: A randomized controlled trial from Pakistan(☆). *J Affect Disord*. 2021;278:78-84. doi:10.1016/j.jad.2020.09.001

149. Jalali F, Hasani A, Hashemi SF, Kimiaei SA, Babaei A. Cognitive Group Therapy Based on Schema-Focused Approach for Reducing Depression in Prisoners Living With HIV. *Int J Offender Ther Comp Criminol*. 2019;63(2):276-288. doi:10.1177/0306624X18784185

150. Jamison C, Scogin F. The outcome of cognitive bibliotherapy with depressed adults. *J Consult Clin Psychol*. 1995;63(4):644-650. doi:10.1037//0022-006x.63.4.644

151. Jarrett RB, Schaffer M, McIntire D, Witt-Browder A, Kraft D, Risser RC. Treatment of atypical depression with cognitive therapy or phenelzine: a double-blind, placebo-controlled trial. *Arch Gen Psychiatry*. 1999;56(5):431-437. doi:10.1001/archpsyc.56.5.431

152. Jelinek L, Hauschildt M, Wittekind C, Schneider B, Kriston L, Moritz S. Efficacy of metacognitive training for depression: A randomized controlled trial. *Psychother Psychosom*. 2016;85(4):231-234. doi:10.1159/000443699

153. Jesse DE, Gaynes BN, Feldhousen EB, Newton ER, Bunch S, Hollon SD. Performance of a Culturally Tailored Cognitive-Behavioral Intervention Integrated in a Public Health Setting to Reduce Risk of Antepartum Depression: A Randomized Controlled Trial. *J Midwifery Womens Health*. 2015;60(5):578-592. doi:10.1111/jmwh.12308

154. Jiang L, Wang ZZ, Qiu LR, Wan GB, Lin Y, Wei Z. Psychological intervention for postpartum depression. *J Huazhong Univ Sci Technol Med Sci*. 2014;34(3):437-442. doi:10.1007/s11596-014-1297-x

155. Johansson R, Ekbladh S, Hebert A, et al. Psychodynamic guided self-help for adult depression through the internet: a randomised controlled trial. *PloS One*. 2012;7(5):e38021. doi:10.1371/journal.pone.0038021

156. Johansson R, Sjˆberg E, Sjˆgren M, et al. Tailored vs. standardized internet-based cognitive behavior therapy for depression and comorbid symptoms: a randomized controlled trial. *PloS One*. 2012;7(5):e36905. doi:10.1371/journal.pone.0036905

157. Johansson O, Bjärehed J, Andersson G, Carlbring P, Lundh LG. Effectiveness of guided internet-delivered cognitive behavior therapy for depression in routine psychiatry: A randomized controlled trial. *Internet Interv*. 2019;17:100247. doi:10.1016/j.invent.2019.100247

158. Johnson JE, Zlotnick C. Pilot study of treatment for major depression among women prisoners with substance use disorder. *J Psychiatr Res*. 2012;46(9):1174-1183. doi:10.1016/j.jpsychires.2012.05.007

159. Johnson JE, Stout RL, Miller TR, et al. Randomized cost-effectiveness trial of group interpersonal psychotherapy (IPT) for prisoners with major depression. *J Consult Clin Psychol*. 2019;87(4):392-406. doi:10.1037/ccp0000379

160. Joling KJ, Hout HP, van't Veer-Tazelaar PJ, et al. How effective is bibliotherapy for very old adults with subthreshold depression? A randomized controlled trial. *Am J Geriatr Psychiatry*. 2011;19(3):256-265. doi:10.1097/JGP.0b013e3181ec8859

161. Jordans MJD, Luitel NP, Garman E, et al. Effectiveness of psychological treatments for depression and alcohol use disorder delivered by community-based counsellors: Two pragmatic randomised controlled trials within primary healthcare in Nepal. *Br J Psychiatry*. 2019;215(2):485-493. doi:10.1192/bjp.2018.300

162. Kamga H, McCusker J, Yaffe M, et al. Self-care tools to treat depressive symptoms in patients with age-related eye disease: a randomized controlled clinical trial. *Clin Experiment Ophthalmol*. 2017;45(4):371-378. doi:10.1111/ceo.12890

163. Kanter JW, Santiago-Rivera AL, Santos MM, et al. A Randomized Hybrid Efficacy and Effectiveness Trial of Behavioral Activation for Latinos With Depression. *Behav Ther*. 2015;46(2):177-192. doi:10.1016/j.beth.2014.09.011

164. Keeley RD, Brody DS, Engel M, et al. Motivational interviewing improves depression outcome in primary care: A cluster randomized trial. *J Consult Clin Psychol*. 2016;84(11):993-1007. doi:10.1037/ccp0000124

165. Kelly JA, Murphy DA, Bahr GR, et al. Outcome of cognitive-behavioral and support group brief therapies for depressed, HIV-infected persons. *Am J Psychiatry*. 1993;150(11):1679-1686. doi:10.1176/ajp.150.11.1679

166. Kenter RMF, Cuijpers P, Beekman A, van Straten A. Effectiveness of a Web-based guided self-help intervention for outpatients with a depressive disorder: Short-term results from a randomized controlled trial. *J Med Internet Res*. 2016;18(3):e80. doi:10.2196/jmir.4861

167. Kessler D, Lewis G, Kaur S, et al. Therapist-delivered Internet psychotherapy for depression in primary care: a randomised controlled trial. *Lancet*. 2009;374(9690):628-634. doi:10.1016/s0140-6736(09)61257-5

168. Kim YH, Choi KS, Han K, Kim HW. A psychological intervention programme for patients with breast cancer under chemotherapy and at a high risk of depression: a randomised clinical trial. *J Clin Nurs*. 2018;27(3-4):572-581. doi:10.1111/jocn.13910

169. King M, Sibbald B, Ward E, et al. Randomised controlled trial of non-directive counselling, cognitive-behaviour therapy and usual general practitioner care in the management of depression as well as mixed anxiety and depression in primary care. *Health Technol Assess*. 2000;4(19):1-83. doi:10.3310/hta4190

170. Kivi M, Eriksson MCM, Hange D, et al. Internet-based therapy for mild to moderate depression in Swedish primary care: Short term results from the PRIM-NET randomized controlled trial. *Cogn Behav Ther*. 2014;43(4):289-298. doi:10.1080/16506073.2014.921834

171. Kleiboer A, Donker T, Seekles W, van Straten A, Riper H, Cuijpers P. A randomized controlled trial on the role of support in Internet-based problem solving therapy for depression and anxiety. *Behav Res Ther*. 2015;72:63-71. doi:10.1016/j.brat.2015.06.013

172. Korrelboom K, Maarsingh M, Huijbrechts I. Competitive memory training (COMET) for treating low self-esteem in patients with depressive disorders: a randomized clinical trial. *Depress Anxiety*. 2012;29(2):102-110. doi:10.1002/da.20921

173. Korte J, Bohlmeijer ET, Cappeliez P, Smit F, Westerhof GJ. Life review therapy for older adults with moderate depressive symptomatology: a pragmatic randomized controlled trial. *Psychol Med*. 2012;42(6):1163-1173. doi:10.1017/S0033291711002042

174. Krämer LV, Grünzig SD, Baumeister H, Ebert DD, Bengel J. Effectiveness of a Guided Web-Based Intervention to Reduce Depressive Symptoms before Outpatient Psychotherapy: A Pragmatic Randomized Controlled Trial. *Psychother Psychosom*. 2021;90(4):233-242. doi:10.1159/000515625

175. Laidlaw K, Davidson K, Toner H, et al. A randomised controlled trial of cognitive behaviour therapy vs treatment as usual in the treatment of mild to moderate late life depression. *Int J Geriatr Psychiatry*. 2008;23(8):843-850. doi:10.1002/gps.1993

176. Lamers F, Jonkers CC, Bosma H, et al. A minimal psychological intervention in chronically ill elderly patients with depression: a randomized trial. *Psychother Psychosom*. 2010;79(4):217-226. doi:10.1159/000313690

177. Lamers SMA, Bohlmeijer ET, Korte J, Westerhof GJ. The efficacy of life-review as online-guided self-help for adults: A randomized trial. *J Gerontol B Psychol Sci Soc Sci*. 2015;70B(1):24-34. doi:10.1093/geronb/gbu030

178. Landreville P, Bissonnette L. Effects of cognitive bibliotherapy for depressed older adults with a disability. *Clin Gerontol*. 1997;17(4):35-55. doi:10.1300/J018v17n04_05

179. Lappalainen P, Langrial S, Oinas-Kukkonen H, Tolvanen A, Lappalainen R. Web-based acceptance and commitment therapy for depressive symptoms with minimal support: A randomized controlled trial. *Behav Modif*. 2015;39(6):805-834. doi:10.1177/0145445515598142

180. Larcombe NA, Wilson PH. An evaluation of cognitive-behaviour therapy for depression in patients with multiple sclerosis. *Br J Psychiatry*. 1984;145:366-371. doi:10.1192/bjp.145.4.366

181. Lee E, Han Y, Cha YJ, et al. Community-Based Multi-Site Randomized Controlled Trial of Behavioral Activation for Patients with Depressive Disorders. *Community Ment Health J*. Published online 2021. doi:10.1007/s10597-021-00828-3

182. Lemma A, Fonagy P. Feasibility study of a psychodynamic online group intervention for depression. *Psychoanal Psychol*. 2013;30(3):367-380. doi:10.1037/a0033239

183. Lenze S, Potts M. Brief Interpersonal Psychotherapy for depression during pregnancy in a low-income population: a randomized controlled trial. *J Affect Disord*. 2017;210:151-157. doi:10.1016/j.jad.2016.12.029

184. Lenze SN, Potts MA, Rodgers J, Luby J. Lessons learned from a pilot randomized controlled trial of dyadic interpersonal psychotherapy for perinatal depression in a low-income population. *J Affect Disord*. 2020;271:286-292. doi:10.1016/j.jad.2020.03.084

185. Lerner D, Adler DA, Rogers WH, et al. A randomized clinical trial of a telephone depression intervention to reduce employee presenteeism and absenteeism. *Psychiatr Serv*. 2015;66(6):570-577. doi:10.1176/appi.ps.201400350

186. Leung SS, Lee AM, Chiang VC, Lam SK, Kuen YW, Wong DF. Culturally sensitive, preventive antenatal group cognitive-behavioural therapy for Chinese women with depression. *Int J Nurs Pr*. 2013;19 Suppl 1:28-37. doi:10.1111/ijn.12021

187. Leung SS, Lee AM, Wong DF, et al. A brief group intervention using a cognitive-behavioural approach to reduce postnatal depressive symptoms: a randomised controlled trial. *Hong Kong Med J*. 2016;22 Suppl 2:S4-8.

188. Lexis MA, Jansen NW, Huibers MJ, et al. Prevention of long-term sickness absence and major depression in high-risk employees: a randomised controlled trial. *Occup Environ Med*. 2011;68(6):400-407. doi:10.1136/oem.2010.057877

189. Liang L, Feng L, Zheng X, Wu Y, Zhang C, Li J. Effect of dialectical behavior group therapy on the anxiety and depression of medical students under the normalization of epidemic prevention and control for the COVID-19 epidemic: a randomized study. *Ann Palliat Med*. 2021;10(10):10591-10599. doi:10.21037/apm-21-2466

190. Linde JA, Simon GE, Ludman EJ, et al. A randomized controlled trial of behavioral weight loss treatment versus combined weight loss/depression treatment among women with comorbid obesity and depression. *Ann Behav Med*. 2011;41(1):119-130. doi:10.1007/s12160-010-9232-2

191. Liu ETH, Chen WL, Li YH, Wang CH, Mok TJ, Huang HS. Exploring the efficacy of cognitive bibliotherapy and a potential mechanism of change in the treatment of depressive symptoms among the Chinese: A randomized controlled trial. *Cogn Ther Res*. 2009;33(5):449-461. doi:10.1007/s10608-008-9228-4

192. Liu H, Yang Y. Effects of a psychological nursing intervention on prevention of anxiety and depression in the postpartum period: a randomized controlled trial. *Ann Gen Psychiatry*. 2021;20(1). doi:10.1186/s12991-020-00320-4

193. Lloyd-Williams M, Shiels C, Ellis J, et al. Pilot randomised controlled trial of focused narrative intervention for moderate to severe depression in palliative care patients: DISCERN trial. *Palliat Med*. 2018;32(1):206-215. doi:10.1177/0269216317711322

194. Lök N, Bademli K, Selçuk-Tosun A. The effect of reminiscence therapy on cognitive functions, depression, and quality of life in Alzheimer patients: randomized controlled trial. *Int J Geriatr Psychiatry*. 2019;34(1):47-53. doi:10.1002/gps.4980

195. Losada A, Márquez-González M, Romero-Moreno R, et al. Cognitive–behavioral therapy (CBT) versus acceptance and commitment therapy (ACT) for dementia family caregivers with significant depressive symptoms: Results of a randomized clinical trial. *J Consult Clin Psychol*. 2015;83(4):760-772. doi:10.1037/ccp0000028

196. Lovell K, Bower P, Richards D, et al. Developing guided self-help for depression using the medical research council complex interventions framework: A description of the modelling phase and results of an exploratory randomised controlled trial. *BMC Psychiatry*. 2008;8:91. doi:10.1186/1471-244X-8-91

197. Lund C, Schneider M, Garman EC, et al. Task-sharing of psychological treatment for antenatal depression in Khayelitsha, South Africa: Effects on antenatal and postnatal outcomes in an individual randomised controlled trial. *Behav Res*. 2019;130:103466. doi:10.1016/j.brat.2019.103466

198. Lundgren J, Dahlstrom Ö, Andersson G, Jaarsma T, Karner Kohlner A, Johansson P. The effect of guided web-based cognitive behavioral therapy on patients with depressive symptoms and heart failure-a randomized controlled trial. *Eur J Heart Fail*. 2016;18(8):401. doi:10.2196/jmir.5556

199. Lustman PJ, Griffith LS, Freedland KE, Kissel SS, Clouse RE. Cognitive behavior therapy for depression in type 2 diabetes mellitus. A randomized, controlled trial. *Ann Intern Med*. 1998;129(8):613-621. doi:10.7326/0003-4819-129-8-199810150-00005

200. Lynch DJ, Tamburrino MB, Nagel R. Telephone counseling for patients with minor depression: preliminary findings in a family practice setting. *J Fam Pr*. 1997;44(3):293-298.

201. Lynch D, Tamburrino M, Nagel R, Smith MK. Telephone-based treatment for family practice patients with mild depression. *Psychol Rep*. 2004;94(3 Pt 1):785-792. doi:10.2466/pr0.94.3.785-792

202. Lynch TR, Hempel RJ, Whalley B, et al. Refractory depression - mechanisms and efficacy of radically open dialectical behaviour therapy (RefraMED): findings of a randomised trial on benefits and harms. *Br J Psychiatry J Ment Sci*. 2019;216(4):1-9. doi:10.1192/bjp.2019.53

203. MacLean S, Corsi DJ, Litchfield S, et al. Coach-facilitated web-based therapy compared with information about web-based resources in patients referred to secondary mental health care for depression: Randomized controlled trial. *J Med Internet Res*. 2020;22(6). doi:10.2196/15001

204. MacPherson H, Richmond S, Bland M, et al. Acupuncture and counselling for depression in primary care: a randomised controlled trial. *PLoS Med*. 2013;10(9):e1001518. doi:10.1371/journal.pmed.1001518

205. Mahmoodi M, Bakhtiyari M, Masjedi Arani A, Mohammadi A, Saberi Isfeedvajani M. The comparison between CBT focused on perfectionism and CBT focused on emotion regulation for individuals with depression and anxiety disorders and dysfunctional perfectionism: A randomized controlled trial. *Behav Cogn Psychother*. 2021;49(4):454-471. doi:10.1017/S1352465820000909

206. Maina G, Forner F, Bogetto F. Randomized controlled trial comparing brief dynamic and supportive therapy with waiting list condition in minor depressive disorders. *Psychother Psychosom*. 2005;74(1):43-50. doi:10.1159/000082026

207. Malouff JM, Lanyon RI, Schutte NS. Effectiveness of a brief group RET treatment for divorce-related dysphoria. *J Ration-Emotive Cogn-Behav Ther*. 1988;6(3):162-171. doi:10.1007/BF01064077

208. Martin PR, Aiello R, Gilson K, Meadows G, Milgrom J, Reece J. Cognitive behavior therapy for comorbid migraine and/or tension-type headache and major depressive disorder: An exploratory randomized controlled trial. *Behav Res Ther*. 2015;73:8-18. doi:10.1016/j.brat.2015.07.005

209. Matsuzaka C, Wainberg M, Norcini PA, et al. Task shifting interpersonal counseling for depression: a pragmatic randomized controlled trial in primary care. *BMC Psychiatry*. 2017;17(1) (no pagination). doi:10.1186/s12888-017-1379-y

210. McClay CA, Collins K, Matthews L, et al. A community-based pilot randomised controlled study of life skills classes for individuals with low mood and depression. *BMC Psychiatry*. 2015;15(1):17. doi:10.1186/s12888-015-0384-2

211. McCusker J, Jones JM, Li M, et al. CanDirect: Effectiveness of a Telephone-Supported Depression Self-Care Intervention for Cancer Survivors. *J Clin Oncol*. 2021;39(10):1150-1161. doi:10.1200/jco.20.01802

212. McIndoo CC, File AA, Preddy T, Clark CG, Hopko DR. Mindfulness-based therapy and behavioral activation: A randomized controlled trial with depressed college students. *Behav Res Ther*. 2016;77:118-128. doi:10.1016/j.brat.2015.12.012

213. McKee MD, Zayas LH, Fletcher J, Boyd RC, Nam SH. Results of an Intervention to Reduce Perinatal Depression Among Low-Income Minority Women in Community Primary Care. *J Soc Serv Res*. 2006;32(4):63-81. doi:10.1300/J079v32n04_04

214. Mennen FE, Palmer Molina A, Monro WL, Duan L, Stuart S, Sosna T. Effectiveness of an Interpersonal Psychotherapy (IPT) Group Depression Treatment for Head Start Mothers: A Cluster-Randomized Controlled Trial. *J Affect Disord*. 2021;280(Pt B):39-48. doi:10.1016/j.jad.2020.11.074

215. Michalak J, Schultze M, Heidenreich T, Schramm E. A randomized controlled trial on the efficacy of mindfulness-based cognitive therapy and a group version of cognitive behavioral analysis system of psychotherapy for chronically depressed patients. *J Consult Clin Psychol*. 2015;83(5):951-963. doi:10.1037/ccp0000042

216. Milgrom J, Negri LM, Gemmill AW, McNeil M, Martin PR. A randomized controlled trial of psychological interventions for postnatal depression. *Br J Clin Psychol*. 2005;44(Pt 4):529-542. doi:10.1348/014466505x34200

217. Milgrom J, Holt CJ, Gemmill AW, et al. Treating postnatal depressive symptoms in primary care: a randomised controlled trial of GP management, with and without adjunctive counselling. *BMC Psychiatry*. 2011;11:95. doi:10.1186/1471-244x-11-95

218. Milgrom J, Holt C, Holt CJ, Ross J, Ericksen J, Gemmill AW. Feasibility study and pilot randomised trial of an antenatal depression treatment with infant follow-up. *Arch Womens Ment Health*. 2015;18(5):717-730. doi:10.1007/s00737-015-0512-5

219. Milgrom J, Danaher BG, Gemmill AW, et al. Internet Cognitive Behavioral Therapy for Women With Postnatal Depression: A Randomized Controlled Trial of MumMoodBooster. *J Med Internet Res*. 2016;18(3):e54. doi:10.2196/jmir.4993

220. Milgrom J, Danaher BG, Gemmill AW, et al. Internet cognitive behavioural therapy for women with postnatal depression: A randomized controlled trial of MumMoodBooster. *Arch Womens Ment Health*. 2020;23(2):293-294. doi:10.1007/s00737-019-00953-9

221. Milgrom J, Danaher BG, Seeley JR, et al. Internet and Face-to-face Cognitive Behavioral Therapy for Postnatal Depression Compared With Treatment as Usual: Randomized Controlled Trial of MumMoodBooster. *J Med Internet Res*. 2021;23(12):e17185. doi:10.2196/17185

222. Miller L, Weissman M. Interpersonal psychotherapy delivered over the telephone to recurrent depressives. A pilot study. *Depress Anxiety*. 2002;16(3):114-117. doi:10.1002/da.10047

223. Miranda J, Chung JY, Green BL, et al. Treating depression in predominantly low-income young minority women: a randomized controlled trial. *Jama*. 2003;290(1):57-65. doi:10.1001/jama.290.1.57

224. Mohr DC, Likosky W, Bertagnolli A, et al. Telephone-administered cognitive-behavioral therapy for the treatment of depressive symptoms in multiple sclerosis. *J Consult Clin Psychol*. 2000;68(2):356-361. doi:10.1037//0022-006x.68.2.356

225. Mohr DC, Carmody T, Erickson L, Jin L, Leader J. Telephone-administered cognitive behavioral therapy for veterans served by community-based outpatient clinics. *J Consult Clin Psychol*. 2011;79(2):261-265. doi:10.1037/a0022395

226. Mohr DC, Duffecy J, Ho J, et al. A randomized controlled trial evaluating a manualized TeleCoaching protocol for improving adherence to a web-based intervention for the treatment of depression. *PLoS One*. 2013;8(8):e70086. doi:10.1371/journal.pone.0070086

227. Moldovan R, Cobeanu O, David D. Cognitive bibliotherapy for mild depressive symptomatology: randomized clinical trial of efficacy and mechanisms of change. *Clin Psychol Psychother*. 2013;20(6):482-493. doi:10.1002/cpp.1814

228. Montero-Marín J, Araya R, Pérez-Yus MC, et al. An internet-based intervention for depression in primary Care in Spain: a randomized controlled trial. *J Med Internet Res*. 2016;18(8):e231. doi:10.2196/jmir.5695

229. Moon JR, Huh J, Song J, et al. The effects of rational emotive behavior therapy for depressive symptoms in adults with congenital heart disease. *Heart Lung*. 2021;50(6):906-913. doi:10.1016/j.hrtlng.2021.07.011

230. Mossey JM, Knott KA, Higgins M, Talerico K. Effectiveness of a psychosocial intervention, interpersonal counseling, for subdysthymic depression in medically ill elderly. *J Gerontol Biol Sci Med Sci*. 1996;51(4):M172-8. doi:10.1093/gerona/51a.4.m172

231. Mukhtar F, Oei TP, Yaacob M. Effectiveness of group cognitive behaviour therapy augmentation in reducing negative cognitions in the treatment of depression in Malaysia. *ASEAN J Psychiatry*. 2011;12(1):50-65.

232. Mulcahy R, Reay RE, Wilkinson RB, Owen C. A randomised control trial for the effectiveness of group Interpersonal Psychotherapy for postnatal depression. *Arch Womens Ment Health*. 2010;13(2):125-139. doi:10.1007/s00737-009-0101-6

233. Musa ZA, Soh KL, Mukhtar F, Soh KY, Oladele TO, Soh KG. Effectiveness of mindfulness-based cognitive therapy among depressed individuals with disabilities in Nigeria: A randomized controlled trial. *Psychiatry Res*. 2021;296:113680. doi:10.1016/j.psychres.2020.113680

234. Mynors-Wallis L, Gath D, Lloyd-Thomas A, Tomlinson D. Randomised controlled trial comparing problem solving treatment with amitriptyline and placebo for major depression in primary care. *Bmj*. 1995;310(6977):441-445. doi:10.1136/bmj.310.6977.441

235. Naeem F, Sarhandi I, Gul M, Khalid M, Aslam M, Anbrin A. A multicentre randomised controlled trial of a carer supervised culturally adapted cbt (cacbt) based self-help for depression in pakistan. *J Affect Disord*. 2013;156:224-227. doi:10.1016/j.jad.2013.10.051

236. Naeem F, Gul M, Irfan M, et al. Brief Culturally adapted CBT (CaCBT) for depression: A randomized controlled trial from Pakistan. *J Affect Disord*. 2015;177:101-107. doi:10.1016/j.jad.2015.02.012

237. Nakagawa A, Mitsuda D, Sado M, et al. Effectiveness of supplementary cognitive-behavioral therapy for pharmacotherapy-resistant depression: A randomized controlled trial. *J Clin Psychiatry*. 2017;78(8):1126-1135. doi:10.4088/JCP.15m10511

238. Nakimuli-Mpungu E, Wamala K, Okello J, et al. Group support psychotherapy for depression treatment in people with HIV/AIDS in northern Uganda: a single-centre randomised controlled trial. *Lancet HIV*. 2015;2(5):e190-9. doi:10.1016/s2352-3018(15)00041-7

239. Nakimuli-Mpungu E, Musisi S, Wamala K, et al. Effectiveness and cost-effectiveness of group support psychotherapy delivered by trained lay health workers for depression treatment among people with HIV in Uganda: a cluster-randomised trial. *Lancet Glob Health*. 2020;8(3):e387-e398. doi:10.1016/s2214-109x(19)30548-0

240. Nasrin F, Rimes K, Reinecke A, Rinck M, Barnhofer T. Effects of Brief Behavioural Activation on Approach and Avoidance Tendencies in Acute Depression: preliminary Findings. *Behav Cogn Psychother*. 2017;45(1):58-72. doi:10.1017/S1352465816000394

241. Neugebauer R, Kline J, Markowitz JC, et al. Pilot randomized controlled trial of interpersonal counseling for subsyndromal depression following miscarriage. *J Clin Psychiatry*. 2006;67(8):1299-1304. doi:10.4088/jcp.v67n0819

242. Newby JM, Lang T, Werner-Seidler A, Holmes E, Moulds ML. Alleviating distressing intrusive memories in depression: A comparison between computerised cognitive bias modification and cognitive behavioural education. *Behav Res Ther*. 2014;56:60-67. doi:10.1016/j.brat.2014.03.001

243. Newby J, Robins L, Wilhelm K, et al. Web-Based Cognitive Behavior Therapy for Depression in People With Diabetes Mellitus: a Randomized Controlled Trial. *J Med Internet Res*. 2017;19(5):e157. doi:10.2196/jmir.7274

244. Nezu AM. Efficacy of a social problem-solving therapy approach for unipolar depression. *J Consult Clin Psychol*. 1986;54(2):196-202. doi:10.1037//0022-006x.54.2.196

245. Nezu AM, Perri MG. Social problem-solving therapy for unipolar depression: an initial dismantling investigation. *J Consult Clin Psychol*. 1989;57(3):408-413. doi:10.1037/0022-006X.57.3.408

246. Ng SE, Tien A, Thayala JN, Ho RC, Chan MF. The effect of life story review on depression of older community-dwelling Chinese adults in Singapore: a preliminary result. *Int J Geriatr Psychiatry*. 2013;28(3):328-330. doi:10.1002/gps.3851

247. Ngai FW, Wong PWC, Leung KY, Chau PH, Chung KF. The Effect of Telephone-Based Cognitive-Behavioral Therapy on Postnatal Depression: A Randomized Controlled Trial. *Psychother Psychosom*. 2015;84(5):294-303. doi:10.1159/000430449

248. Niedermoser DW, Kalak N, Kiyhankhadiv A, et al. Workplace-Related Interpersonal Group Psychotherapy to Improve Life at Work in Individuals With Major Depressive Disorders: a Randomized Interventional Pilot Study. *Front Psychiatry*. 2020;11. doi:10.3389/fpsyt.2020.00168

249. Nobis S, Lehr D, Ebert DD, et al. Efficacy of a Web-Based Intervention With Mobile Phone Support in Treating Depressive Symptoms in Adults With Type 1 and Type 2 Diabetes: A Randomized Controlled Trial. *Diabetes Care*. 2015;38(5):776-783. doi:10.2337/dc14-1728

250. Nollett CL, Bray N, Bunce C, et al. Depression in visual impairment trial (Depvit): A randomized clinical trial of depression treatments in people with low vision. *Invest Ophthalmol Vis Sci*. 2016;57(10):4247-4254. doi:10.1167/iovs.16-19345

251. Northwood AK, Vukovich MM, Beckman A, et al. Intensive psychotherapy and case management for Karen refugees with major depression in primary care: a pragmatic randomized control trial. *BMC Fam Pr*. 2020;21(1):17. doi:10.1186/s12875-020-1090-9

252. Nyström M, Stenling A, Sjöström E, et al. Behavioral activation versus physical activity via the internet: a randomized controlled trial. *J Affect Disord*. 2017;215:85-93. doi:10.1016/j.jad.2017.03.018

253. O'Hara MW, Stuart S, Gorman LL, Wenzel A. Efficacy of interpersonal psychotherapy for postpartum depression. *Arch Gen Psychiatry*. 2000;57(11):1039-1045. doi:10.1001/archpsyc.57.11.1039

254. O'Mahen H, Himle JA, Fedock G, Henshaw E, Flynn H. A pilot randomized controlled trial of cognitive behavioral therapy for perinatal depression adapted for women with low incomes. *Depress Anxiety*. 2013;30(7):679-687. doi:10.1002/da.22050

255. O'Neil A, Taylor B Fau - Sanderson K, Sanderson K Fau - Cyril S, et al. Efficacy and feasibility of a tele-health intervention for acute coronary syndrome patients with depression: results of the "MoodCare" randomized controlled trial. *Ann Behav Med*. 2014;48(1532-4796 (Electronic)):163-174. doi:10.1007/s12160-014-9592-0

256. Oehler C, Görges F, Rogalla M, Rummel-Kluge C, Hegerl U. Efficacy of a Guided Web-Based Self-Management Intervention for Depression or Dysthymia: Randomized Controlled Trial

With a 12-Month Follow-Up Using an Active Control Condition. *J Med Internet Res*. 2020;22(7):e15361. doi:10.2196/15361

257. Olukolade O, Osinowo H. Efficacy of Cognitive Rehabilitation Therapy on Poststroke Depression among Survivors of First Stroke Attack in Ibadan, Nigeria. *Behav Neurol*. 2017;2017(no pagination). doi:10.1155/2017/4058124

258. Omidi A, Mohammadkhani P, Mohammadi A, Zargar F. Comparing mindfulness based cognitive therapy and traditional cognitive behavior therapy with treatments as usual on reduction of major depressive disorder symptoms. *Iran Red Crescent Med J*. 2013;15(2):142-146. doi:10.5812/ircmj.8018

259. Onuigbo LN, Eseadi C, Ebifa S, Ugwu UC, Onyishi CN, Oyeoku EK. Effect of rational emotive behavior therapy program on depressive symptoms among university students with blindness in Nigeria. *J Ration-Emotive Cogn-Behav Ther*. 2019;37(1):17-38. doi:10.1007/s10942-018-0297-3

260. Onyechi KCN, Eseadi C, Okere AU, et al. Effects of cognitive behavioral coaching on depressive symptoms in a sample of type 2 diabetic inpatients in Nigeria. *Med U S*. 2016;95(31):e4444. doi:10.1097/MD.0000000000004444

261. Pace TM, Dixon DN. Changes in depressive self-schemata and depressive symptoms following cognitive therapy. *J Couns Psychol*. 1993;40(3):288. doi:10.1037/0022-0167.40.3.288

262. Pagoto S, Schneider KL, Whited MC, et al. Randomized controlled trial of behavioral treatment for comorbid obesity and depression in women: the Be Active Trial. *Int J Obes*. 2013;37(11):1427-1434. doi:10.1038/ijo.2013.25

263. Patel V, Weobong B, Weiss H, et al. The Healthy Activity Program (HAP), a lay counsellor-delivered brief psychological treatment for severe depression, in primary care in India: a randomised controlled trial. *Lancet Lond Engl*. 2017;389(10065):176-185. doi:10.1016/S0140-6736(16)31589-6

264. Pecheur DR, Edwards KJ. A comparison of secular and religious versions of cognitive therapy with depressed Christian college students. *J Psychol Theol*. Published online 1984. doi:10.1177/009164718401200106

265. Peden AR, Hall LA, Rayens MK, Beebe LL. Reducing negative thinking and depressive symptoms in college women. *J Nurs Sch*. 2000;32(2):145-151. doi:10.1111/j.1547-5069.2000.00145.x

266. Penckofer SM, Ferrans C, Mumby P, et al. A psychoeducational intervention (SWEEP) for depressed women with diabetes. *Ann Behav Med*. 2012;44(2):192-206. doi:10.1007/s12160-012-9377-2

267. Perini S, Titov N, Andrews G. Clinician-assisted Internet-based treatment is effective for depression: randomized controlled trial. *Aust N Z J Psychiatry*. 2009;43(6):571-578. doi:10.1080/00048670902873722

268. Petersen I, Hanass Hancock J, Bhana A, Govender K. A group-based counselling intervention for depression comorbid with HIV/AIDS using a task shifting approach in South Africa: a randomized controlled pilot study. *J Affect Disord*. 2014;158:78-84. doi:10.1016/j.jad.2014.02.013

269. Pibernik-Okanovic M, Begic D, Ajdukovic D, Andrijasevic N, Metelko Z. Psychoeducation versus treatment as usual in diabetic patients with subthreshold depression: preliminary results of a randomized controlled trial. *Trials*. 2009;10:78. doi:10.1186/1745-6215-10-78

270. Pibernik-Okanovic M, Hermanns N, Ajdukovic D, et al. Does treatment of subsyndromal depression improve depression-related and diabetes-related outcomes? A randomised controlled comparison of psychoeducation, physical exercise and enhanced treatment as usual. *Trials*. 2015;16:305. doi:10.1186/s13063-015-0833-8

271. Pihlaja S, Lahti J, Lipsanen JO, et al. Scheduled Telephone Support for Internet Cognitive Behavioral Therapy for Depression in Patients at Risk for Dropout: Pragmatic Randomized Controlled Trial. *J Med Internet Res*. 2020;22(7):e15732. doi:10.2196/15732

272. Poleshuck EL, Gamble SA, Bellenger K, et al. Randomized controlled trial of interpersonal psychotherapy versus enhanced treatment as usual for women with co-occurring depression and pelvic pain. *J Psychosom Res*. 2014;77(4):264-272. doi:10.1016/j.jpsychores.2014.07.016

273. Pot AM, Bohlmeijer ET, Onrust S, Melenhorst AS, Veerbeek M, De Vries W. The impact of life review on depression in older adults: A randomized controlled trial. *Int Psychogeriatr*. 2010;22(4):572-581. doi:10.1017/S104161020999175X

274. Pots WTM, Meulenbeek PAM, Veehof MM, Klungers J, Bohlmeijer ET. The efficacy of mindfulness-based cognitive therapy as a public mental health intervention for adults with mild to moderate depressive symptomatology: A randomized controlled trial. *PLoS ONE*. 2014;9(10). doi:10.1371/journal.pone.0109789

275. Pots W, Fledderus M, Meulenbeek P, Klooster P, Schreurs K, Bohlmeijer E. Acceptance and commitment therapy as a web-based intervention for depressive symptoms: randomised controlled trial. *Br J Psychiatry J Ment Sci*. 2016;208(1):69-77. doi:10.1192/bjp.bp.114.146068

276. Power Michael J, Freeman C. A randomized controlled trial of IPT versus CBT in primary care: With some cautionary notes about handling missing values in clinical trials. [References]. *Clin Psychol Psychother*. 2012;19(2):159-169. doi:10.1002/cpp.1781

277. Prendergast J, Austin MP. Early childhood nurse-delivered cognitive behavioural counselling for post-natal depression. *Australas Psychiatry*. 2001;9(3):255-259. doi:10.1046/j.1440-1665.2001.00330.x

278. Preschl B, Maercker A, Wagner B, et al. Life-review therapy with computer supplements for depression in the elderly: a randomized controlled trial. *Aging Ment Health*. 2012;16(8):964-974. doi:10.1080/13607863.2012.702726

279. Propst LR, Ostrom R, Watkins P, Dean T, Mashburn D. Comparative efficacy of religious and nonreligious cognitive-behavioral therapy for the treatment of clinical depression in religious individuals. *J Consult Clin Psychol*. 1992;60(1):94-103. doi:10.1037//0022-006x.60.1.94

280. Psarraki EE, Bacopoulou F, Panagoulias E, et al. The effects of Pythagorean Self-Awareness Intervention on patients with major depressive disorder: A pilot randomized controlled trial. *J Psychiatr Res*. 2021;138:326-334. doi:10.1016/j.jpsychires.2021.03.067

281. Puckering C, McIntosh E, Hickey A, Longford J. Mellow Babies: A group intervention for infants and mothers experiencing postnatal depression. *Couns Psychol Rev*. 2010;25(1):28-38.

282. Pugh NE, Hadjistavropoulos HD, Dirkse D. A Randomised Controlled Trial of Therapist-Assisted, Internet-Delivered Cognitive Behavior Therapy for Women with Maternal Depression. *PLoS One*. 2016;11(3):e0149186. doi:10.1371/journal.pone.0149186

283. Qiu J, Chen W, Gao X, et al. A randomized controlled trial of group cognitive behavioral therapy for Chinese breast cancer patients with major depression. *J Psychosom Obstet Gynaecol*. 2013;34(2):60-67. doi:10.3109/0167482x.2013.766791

284. Raevuori A, Vahlberg T, Korhonen T, Hilgert O, Aittakumpu-Hyden R, Forman-Hoffman V. A therapist-guided smartphone app for major depression in young adults: A randomized clinical trial. *J Affect Disord*. 2021;286:228-238. doi:10.1016/j.jad.2021.02.007

285. Rahman A, Malik A, Sikander S, Roberts C, Creed F. Cognitive behaviour therapy-based intervention by community health workers for mothers with depression and their infants in rural Pakistan: a cluster-randomised controlled trial. *Lancet*. 2008;372(9642):902-909. doi:10.1016/s0140-6736(08)61400-2

286. Ransom D, Heckman TG, Anderson T, Garske J, Holroyd K, Basta T. Telephone-delivered, interpersonal psychotherapy for HIV-infected rural persons with depression: a pilot trial. *Psychiatr Serv*. 2008;59(8):871-877. doi:10.1176/appi.ps.59.8.871

287. Raue PJ, Sirey JA, Dawson A, Berman J, Bruce ML. Lay-delivered behavioral activation for depressed senior center clients: Pilot RCT. *Int J Geriatr Psychiatry*. 2019;34(11):1715-1723. doi:10.1002/gps.5186

288. Raya-Tena A, Fernández-San-Martin MI, Martin-Royo J, et al. Effectiveness of a Psychoeducational Group Intervention Carried Out by Nurses for Patients with Depression and Physical Comorbidity in Primary Care: Randomized Clinical Trial. *Int J Env Res Public Health*. 2021;18(6). doi:10.3390/ijerph18062948

289. Rehm LP, Kornblith SJ, O'Hara MW, Lamparski DM, Romano JM, Volkin JI. An evaluation of major components in a self-control therapy program for depression. *Behav Modif*. 1981;5(4):459-489. doi:10.1177/014544558154002

290. Reins JA, Boß L, Lehr D, Berking M, Ebert DD. The more I got, the less I need? Efficacy of Internet-based guided self-help compared to online psychoeducation for major depressive disorder. *J Of*. 2019;246:695-705. doi:10.1016/j.jad.2018.12.065

291. Richards D, Timulak L, O'Brien E, et al. A randomized controlled trial of an internet-delivered treatment: Its potential as a low-intensity community intervention for adults with symptoms of depression. *Behav Res Ther*. 2015;75:20-31. doi:10.1016/j.brat.2015.10.005

292. Richards SH, Dickens C, Anderson R, et al. Assessing the effectiveness of Enhanced Psychological Care for patients with depressive symptoms attending cardiac rehabilitation compared with treatment as usual (CADENCE): a pilot cluster randomised controlled trial. *Trials*. 2018;19(1):211. doi:10.1186/s13063-018-2576-9

293. Rief W, Bleichhardt G, Dannehl K, Euteneuer F, Wambach K. Comparing the Efficacy of CBASP with Two Versions of CBT for Depression in a Routine Care Center: a Randomized Clinical Trial. *Psychother Psychosom*. 2018;(no pagination)(3):164-178. doi:10.1159/000487893

294. Ritvo P, Knyahnytska Y, Pirbaglou M, et al. Online Mindfulness-Based Cognitive Behavioral Therapy Intervention for Youth With Major Depressive Disorders: Randomized Controlled Trial. *J Med Internet Res*. 2021;23(3):e24380. doi:10.2196/24380

295. Rizvi SJ, Zaretsky A, Schaffer A, Levitt A. Is immediate adjunctive CBT more beneficial than delayed CBT in treating depression?: A Pilot Study. *J Psychiatr Pr*. 2015;21(2):107-113. doi:10.1097/01.pra.0000462603.71983.15

296. Rohan KJ, Roecklein KA, Lindsey KT, et al. A Randomized Controlled Trial of Cognitive-Behavioral Therapy, Light Therapy, and Their Combination for Seasonal Affective Disorder. *J Consult Clin Psychol*. 2007;75(3):489-500. doi:10.1037/0022-006X.75.3.489

297. Rohde P, Stice E, Shaw H, Gau JM. Pilot trial of a dissonance-based cognitive-behavioral group depression prevention with college students. *Behav Res Ther*. 2016;82:21-27. doi:10.1016/j.brat.2016.05.001

298. Rohricht F, Papadopoulos N, Priebe S. An exploratory randomized controlled trial of body psychotherapy for patients with chronic depression. *J Affect Disord*. 2013;151(1):85-91. doi:10.1016/j.jad.2013.05.056

299. Ross M, Scott M. An evaluation of the effectiveness of individual and group cognitive therapy in the treatment of depressed patients in an inner city health centre. *J R Coll Gen Pr*. 1985;35(274):239-242.

300. Rosso I, Killgore W, Olson E, et al. Internet-based cognitive behavior therapy for major depressive disorder: a randomized controlled trial. *Depress Anxiety*. 2017;34(3):236-245. doi:10.1002/da.22590

301. Rude SS. Relative benefits of assertion or cognitive self-control treatment for depression as a function of proficiency in each domain. *J Consult Clin Psychol*. 1986;54(3):390-394. doi:10.1037//0022-006x.54.3.390

302. Russell A, Gaunt DM, Cooper K, et al. The feasibility of low-intensity psychological therapy for depression co-occurring with autism in adults: the Autism Depression Trial (ADEPT) â€“ a pilot randomised controlled trial. *Autism*. 2019;24(6):1360-1372. doi:10.1177/1362361319889272

303. Ruwaard J, Schrieken B, Schrijver M, et al. Standardized web-based cognitive behavioural therapy of mild to moderate depression: a randomized controlled trial with a long-term follow-up. *Cogn Behav Ther*. 2009;38(4):206-221. doi:10.1080/16506070802408086

304. Ruzickova T, Carson J, Murphy S, Harmer C. P.230 Effects of online behavioural activation on depression during covid-19. *Eur Neuropsychopharmacol*. 2021;44:S36-S37. doi:10.1016/j.euroneuro.2021.01.059

305. Safren SA, O'Cleirigh C, Tan JY, et al. A randomized controlled trial of cognitive behavioral therapy for adherence and depression (CBT-AD) in HIV-infected individuals. *Health Psychol*. 2009;28(1):1-10. doi:10.1037/a0012715

306. Safren SA, Gonzalez JS, Wexler DJ, et al. A randomized controlled trial of cognitive behavioral therapy for adherence and depression (CBT-AD) in patients with uncontrolled type 2 diabetes. *Diabetes Care*. 2014;37(3):625-633. doi:10.2337/dc13-0816

307. Safren SA, Bedoya CA, O'Cleirigh C, et al. Cognitive behavioural therapy for adherence and depression in patients with HIV: a three-arm randomised controlled trial. *Lancet HIV*. 2016;3(11):e529-e538. doi:10.1016/S2352-3018(16)30053-4

308. Safren SA, O'Cleirigh C, Andersen LS, et al. Treating depression and improving adherence in HIV care with task-shared cognitive behavioural therapy in Khayelitsha, South Africa: a randomized controlled trial. *J Int AIDS Soc*. 2021;24(10):e25823. doi:10.1002/jia2.25823

309. Salamanca-Sanabria A, Richards D, Timulak L, et al. A culturally adapted cognitive behavioral internet-delivered intervention for depressive symptoms: Randomized controlled trial. *JMIR Ment Health*. 2020;7(1). doi:10.2196/13392

310. Saloheimo HP, Markowitz J, Saloheimo TH, et al. Psychotherapy effectiveness for major depression: a randomized trial in a Finnish community. *BMC Psychiatry*. 2016;16:131. doi:10.1186/s12888-016-0838-1

311. Sander LB, Paganini S, Terhorst Y, et al. Effectiveness of a Guided Web-Based Self-help Intervention to Prevent Depression in Patients with Persistent Back Pain: The PROD-BP Randomized Clinical Trial. *JAMA Psychiatry*. 2020;77(10):1001-1011. doi:10.1001/jamapsychiatry.2020.1021

312. Savard J, Simard S, Giguere I, et al. Randomized clinical trial on cognitive therapy for depression in women with metastatic breast cancer: psychological and immunological effects. *Palliat Support Care*. 2006;4(3):219-237. doi:10.1017/s1478951506060305

313. Savari Y, Mohagheghi H, Petrocchi N. A preliminary investigation on the effectiveness of compassionate mind training for students with major depressive disorder: A randomized controlled trial. *Mindfulness*. 2021;12(5):1159-1172. doi:10.1007/s12671-020-01584-3

314. Schlicker S, Baumeister H, Buntrock C, et al. A web- And mobile-based intervention for comorbid, recurrent depression in patients with chronic back pain on sick leave (get.back): Pilot randomized controlled trial on feasibility, user satisfaction, and effectiveness. *JMIR Ment Health*. 2020;7(4). doi:10.2196/16398

315. Schmidt MM, Miller WR. Amount of therapist contact and outcome in a multidimensional depression treatment program. *Acta Psychiatr Scand*. 1983;67(5):319-332. doi:10.1111/j.1600-0447.1983.tb00349.x

316. Schramm E, Mack S, Thiel N, Jenkner C, Elsaesser M, Fangmeier T. Interpersonal psychotherapy vs treatment as usual for major depression related to work stress: A pilot randomized controlled study. *Front Psychiatry*. 2020;11. doi:10.3389/fpsyt.2020.00193

317. Schulberg HC, Block MR, Madonia MJ, et al. Treating major depression in primary care practice. Eight-month clinical outcomes. *Arch Gen Psychiatry*. 1996;53(10):913-919. doi:10.1001/archpsyc.1996.01830100061008

318. Schuster R, Leitner I, Carlbring P, Laireiter AR. Exploring blended group interventions for depression: randomised controlled feasibility study of a blended computer- and multimedia-supported psychoeducational group intervention for adults with depressive symptoms. *Internet Interv*. 2017;8:63-71. doi:10.1016/j.invent.2017.04.001

319. Scogin F, Hamblin D, Beutler L. Bibliotherapy for depressed older adults: A self-help alternative. *The Gerontologist*. 1987;27(3):383-387. doi:10.1093/geront/27.3.383

320. Scogin F, Jamison C, Gochneaur K. Comparative efficacy of cognitive and behavioral bibliotherapy for mildly and moderately depressed older adults. *J Consult Clin Psychol*. 1989;57(3):403-407. doi:10.1037//0022-006x.57.3.403

321. Scogin FR, Moss K, Harris GM, Presnell AH. Treatment of depressive symptoms in diverse, rural, and vulnerable older adults. *Int J Geriatr Psychiatry*. 2014;29(3):310-316. doi:10.1002/gps.4009

322. Scott MJ, Stradling SG. Group cognitive therapy for depression produces clinically significant reliable change in community-based settings. *Behav Psychother*. 1990;18(01):1-19. doi:10.1017/S014134730001795X

323. Scott AI, Freeman CP. Edinburgh primary care depression study: treatment outcome, patient satisfaction, and cost after 16 weeks. *Bmj*. 1992;304(6831):883-887. doi:10.1136/bmj.304.6831.883

324. Scott C, Tacchi MJ, Jones R, Scott J. Acute and one-year outcome of a randomised controlled trial of brief cognitive therapy for major depressive disorder in primary care. *Br J Psychiatry*. 1997;171:131-134. doi:10.1192/bjp.171.2.131

325. Segre LS, Brock RL, O'Hara MW. Depression treatment for impoverished mothers by point-of-care providers: A randomized controlled trial. *J Consult Clin Psychol*. 2015;83(2):314-324. doi:10.1037/a0038495

326. Selmi PM, Klein MH, Greist JH, Sorrell SP, Erdman HP. Computer-administered cognitive-behavioral therapy for depression. *Am J Psychiatry*. 1990;147(1):51-56. doi:10.1176/ajp.147.1.51

327. Serfaty MA, Haworth D, Blanchard M, Buszewicz M, Murad S, King M. Clinical effectiveness of individual cognitive behavioral therapy for depressed older people in primary care: a randomized controlled trial. *Arch Gen Psychiatry*. 2009;66(12):1332-1340. doi:10.1001/archgenpsychiatry.2009.165

328. Serfaty M, King M, Nazareth I, et al. Effectiveness of cognitive-behavioural therapy for depression in advanced cancer: CanTalk randomised controlled trial. *Br J Psychiatry J Ment Sci*. 2019;216(4):1-9. doi:10.1192/bjp.2019.207

329. Serrano Selva JP, Latorre Postigo JM, Ros Segura L, et al. Life review therapy using autobiographical retrieval practice for older adults with clinical depression. *Psicothema*. 2012;24(2):224-229.

330. Serrano JP, Latorre JM, Gatz M, Montanes J. Life review therapy using autobiographical retrieval practice for older adults with depressive symptomatology. *Psychol Aging*. 2004;19(2):270-277. doi:10.1037/0882-7974.19.2.270

331. Shaw BF. Comparison of cognitive therapy and behavior therapy in the treatment of depression. *J Consult Clin Psychol*. 1977;45(4):543. doi:10.1037//0022-006x.45.4.543

332. Sheeber LB, Seeley JR, Feil EG, et al. Development and pilot evaluation of an Internet-facilitated cognitive-behavioral intervention for maternal depression. *J Consult Clin Psychol*. 2012;80(5):739-749. doi:10.1037/a0028820

333. Sheeber L, Feil E, Seeley J, et al. Mom-net: evaluation of an internet-facilitated cognitive behavioral intervention for low-income depressed mothers. *J Consult Clin Psychol*. 2017;85(4):355-366. doi:10.1037/ccp0000175

334. Shih VWY, Chan WC, Tai OK, Wong HL, Cheng CPW, Wong CSM. Mindfulness-Based Cognitive Therapy for Late-Life Depression: a Randomised Controlled Trial. *East Asian Arch Psychiatry*. 2021;31(2):27-35. doi:10.12809/eaap2075

335. Sikander S, Ahmad I, Atif N, et al. Delivering the Thinking Healthy Programme for perinatal depression through volunteer peers: a cluster randomised controlled trial in Pakistan. *Lancet Psychiatry*. 2019;6(2):128-139. doi:10.1016/S2215-0366(18)30467-X

336. Simoni JM, Wiebe JS, Sauceda JA, et al. A preliminary RCT of CBT-AD for adherence and depression among HIV-positive Latinos on the U.S.-Mexico border: the Nuevo Dia study. *AIDS Behav*. 2013;17(8):2816-2829. doi:10.1007/s10461-013-0538-5

337. Simpson S, Corney R, Beecham J. A randomized controlled trial to evaluate the effectiveness and cost-effectiveness of psychodynamic counselling for general practice patients with chronic depression. *Psychol Med*. 2003;33(2):229-239. doi:10.1017/s0033291702006517

338. Simson U, Nawarotzky U, Friese G, et al. Psychotherapy intervention to reduce depressive symptoms in patients with diabetic foot syndrome. *Diabet Med*. 2008;25(2):206-212. doi:10.1111/j.1464-5491.2007.02370.x

339. Sinniah A, Oei T, Maniam T, Subramaniam P. Positive effects of Individual Cognitive Behavior Therapy for patients with unipolar mood disorders with suicidal ideation in Malaysia: a randomised controlled trial. *Psychiatry Res*. 2017;254:179-189. doi:10.1016/j.psychres.2017.04.026

340. Sirey JA, Solomonov N, Guillod A, et al. PROTECT: a novel psychotherapy for late-life depression in elder abuse victims. *Int Psychogeriatr*. 2021;33(5):521-525. doi:10.1017/s1041610221000430

341. Smit A, Kluiter H, Conradi HJ, et al. Short-term effects of enhanced treatment for depression in primary care: results from a randomized controlled trial. *Psychol Med*. 2006;36(1):15-26. doi:10.1017/S0033291705006318

342. Smith J, Newby JM, Burston N, et al. Help from home for depression: A randomised controlled trial comparing internet-delivered cognitive behaviour therapy with bibliotherapy for depression. *Internet Interv*. 2017;9:25-37. doi:10.1016/j.invent.2017.05.001

343. Songprakun W, McCann TV. Evaluation of a cognitive behavioural self-help manual for reducing depression: a randomized controlled trial. *J Psychiatr Ment Health Nurs*. 2012;19(7):647-653. doi:10.1111/j.1365-2850.2011.01861.x

344. Spek V, Nyklicek I, Smits N, et al. Internet-based cognitive behavioural therapy for subthreshold depression in people over 50 years old: a randomized controlled clinical trial. *Psychol Med*. 2007;37(12):1797-1806. doi:10.1017/s0033291707000542

345. Spinelli MG, Endicott J. Controlled clinical trial of interpersonal psychotherapy versus parenting education program for depressed pregnant women. *Am J Psychiatry*. 2003;160(3):555-562. doi:10.1176/appi.ajp.160.3.555

346. Spinelli MG, Endicott J, Leon AC, et al. A controlled clinical treatment trial of interpersonal psychotherapy for depressed pregnant women at 3 New York City sites. *J Clin Psychiatry*. 2013;74(4):393-399. doi:10.4088/JCP.12m07909

347. Spruill TM, Friedman D, Diaz L, et al. Telephone-based depression self-management in Hispanic adults with epilepsy: a pilot randomized controlled trial. *Transl Behav Med*. 2021;11(7):1451-1460. doi:10.1093/tbm/ibab045

348. Sreevani R, Reddemma K, Chan CL, Leung PP, Wong V, Chan CH. Effectiveness of integrated body-mind-spirit group intervention on the well-being of Indian patients with depression: a pilot study. *J Nurs Res*. 2013;21(3):179-186. doi:10.1097/jnr.0b013e3182a0b041

349. Stiles-Shields C, Montague E, Kwasny MJ, Mohr DC. Behavioral and cognitive intervention strategies delivered via coached apps for depression: Pilot trial. *Psychol Serv*. 2018;16(2):233-238. doi:10.1037/ser0000261

350. Strauss C, Hayward M, Chadwick P. Group person-based cognitive therapy for chronic depression: a pilot randomized controlled trial. *Br J Clin Psychol*. 2012;51(3):345-350. doi:10.1111/j.2044-8260.2012.02036.x

351. Strong V, Waters R, Hibberd C, et al. Management of depression for people with cancer (SMaRT oncology 1): a randomised trial. *Lancet*. 2008;372(9632):40-48. doi:10.1016/s0140-6736(08)60991-5

352. Sugg HVR, Richards DA, Frost J. Morita Therapy for depression (Morita Trial): a pilot randomised controlled trial. *BMJ Open*. 2018;8(8):e021605. doi:10.1136/bmjopen-2018-021605

353. Swartz HA, Frank E, Zuckoff A, et al. Brief interpersonal psychotherapy for depressed mothers whose children are receiving psychiatric treatment. *Am J Psychiatry*. 2008;165(9):1155-1162. doi:10.1176/appi.ajp.2008.07081339

354. Szumska I, Gola M, Rusanowska M, et al. Mindfulness-based cognitive therapy reduces clinical symptoms, but do not change frontal alpha asymmetry in people with major depression disorder. *Int J Neurosci*. Published online 2020. doi:10.1080/00207454.2020.1748621

355. Takagaki K, Okamoto Y, Jinnin R, et al. Behavioral activation for late adolescents with subthreshold depression: a randomized controlled trial. *Eur Child Adolesc Psychiatry*. 2016;25(11):1171-1182. doi:10.1007/s00787-016-0842-5

356. Talbot NL, Chaudron LH, Ward EA, et al. A randomized effectiveness trial of interpersonal psychotherapy for depressed women with sexual abuse histories. *Psychiatr Serv*. 2011;62(4):374-380. doi:10.1176/appi.ps.62.4.374

357. Taylor FG, Marshall WL. Experimental analysis of a cognitive-behavioral therapy for depression. *Cogn Ther Res*. 1977;1(1):59-72. doi:10.1007/BF01173505

358. Taylor CB, Conrad A, Wilhelm FH, et al. Does improving mood in depressed patients alter factors that may affect cardiovascular disease risk? *J Psychiatr Res*. 2009;43(16):1246-1252. doi:10.1016/j.jpsychires.2009.05.006

359. Teasdale JD, Fennell MJ, Hibbert GA, Amies PL. Cognitive therapy for major depressive disorder in primary care. *Br J Psychiatry*. 1984;144:400-406. doi:10.1192/bjp.144.4.400

360. Teichman Y, Bar-el Z, Shor H, Sirota P, Elizur A. A comparison of two modalities of cognitive therapy (individual and marital) in treating depression. *Psychiatry*. 1995;58(2):136-148. doi:10.1080/00332747.1995.11024721

361. Teri L, Logsdon RG, Uomoto J, McCurry SM. Behavioral treatment of depression in dementia patients: a controlled clinical trial. *J Gerontol B Psychol Sci Soc Sci*. 1997;52(4):P159-66. doi:10.1093/geronb/52b.4.p159

362. Thomas SA, Drummond AE, Lincoln NB, et al. Behavioural activation therapy for post-stroke depression: the BEADS feasibility RCT. *Health Technol Assess Winch Engl*. 2019;23(47):1-176. doi:20190916111043841

363. Titov N, Andrews G, Davies M, McIntyre K, Robinson E, Solley K. Internet treatment for depression: a randomized controlled trial comparing clinician vs. technician assistance. *PLoS One*. 2010;5(6):e10939. doi:10.1371/journal.pone.0010939

364. Titov N, Dear BF, Ali S, et al. Clinical and cost-effectiveness of therapist-guided internet-delivered cognitive behavior therapy for older adults with symptoms of depression: a randomized controlled trial. *Behav Ther*. 2015;46(2):193-205. doi:10.1016/j.beth.2014.09.008

365. Tobin K, Davey-Rothwell MA, Nonyane BAS, Knowlton A, Wissow L, Latkin CA. RCT of an integrated CBT-HIV intervention on depressive symptoms and HIV risk. *PLoS One*. 2017;12(12):e0187180. doi:10.1371/journal.pone.0187180

BMJ Publishing Group Limited (BMJ) disclaims all liability and responsibility arising from any reliance placed on this supplemental material which has been supplied by the author(s)

366. Tomasino K, Lattie E, Ho J, Palac H, Kaiser S, Mohr D. Harnessing Peer Support in an Online Intervention for Older Adults with Depression. *Am J Geriatr Psychiatry*. 2017;25(10):1109-1119. doi:10.1016/j.jagp.2017.04.015

367. Tong P, Bu P, Yang Y, Dong L, Sun T, Shi Y. Group cognitive behavioural therapy can reduce stigma and improve treatment compliance in major depressive disorder patients. *Early Interv Psychiatry*. 2019;14(2):172-178. doi:10.1111/eip.12841

368. Tovote KA, Fleer J, Snippe E, et al. Individual mindfulness-based cognitive therapy and cognitive behavior therapy for treating depressive symptoms in patients with diabetes: Results of a randomized controlled trial. *Diabetes Care*. 2014;37(9):2427-2434. doi:10.2337/dc13-2918

369. Town J, Abbass A, Stride C, Bernier D. A randomised controlled trial of Intensive Short-Term Dynamic Psychotherapy for treatment resistant depression: the Halifax Depression Study. *J Affect Disord*. 2017;214:15-25. doi:10.1016/j.jad.2017.02.035

370. Trevillion K, Ryan EG, Pickles A, et al. An exploratory parallel-group randomised controlled trial of antenatal Guided Self-Help (plus usual care) versus usual care alone for pregnant women with depression: DAWN trial. *J Affect Disord*. 2020;261:187-197. doi:10.1016/j.jad.2019.10.013

371. Tulbure BT, Andersson G, Salagean N, Pearce M, Koenig HG. Religious versus Conventional Internet-based Cognitive Behavioral Therapy for Depression. *J Relig Health*. 2018;57(5):1634-1648. doi:10.1007/s10943-017-0503-0

372. Turner RW, Ward MF, Turner DJ. Behavioral treatment for depression: An evaluation of therapeutic components. *J Clin Psychol*. 1979;35(1):166-175. doi:10.1002/1097-4679(197901)35:1<166::aid-jclp2270350127>3.0.co;2-1

373. Turner A, Hambridge J, Baker A, Bowman J, McElduff P. Randomised controlled trial of group cognitive behaviour therapy versus brief intervention for depression in cardiac patients. *Aust N Z J Psychiatry*. 2013;47(3):235-243. doi:10.1177/0004867412460592

374. Tyson GM, Range LM. Gestalt dialogues as a treatment for mild depression: time works just as well. *J Clin Psychol*. 1987;43(2):227-231. doi:10.1002/1097-4679(198703)43:2<227::aid-jclp2270430210>3.0.co;2-m

375. Unlu Ince B, Cuijpers P, t Hof E, Ballegooijen W, Christensen H, Riper H. Internet-based, culturally sensitive, problem-solving therapy for Turkish migrants with depression: randomized controlled trial. *J Med Internet Res*. 2013;15(10):e227. doi:10.2196/jmir.2853

376. van Bastelaar KM, Pouwer F, Cuijpers P, Riper H, Snoek FJ. Web-based depression treatment for type 1 and type 2 diabetic patients: a randomized, controlled trial. *Diabetes Care*. 2011;34(2):320-325. doi:10.2337/dc10-1248

377. Van Lieshout RJ, Layton H, Savoy CD, et al. Effect of Online 1-Day Cognitive Behavioral Therapy-Based Workshops Plus Usual Care vs Usual Care Alone for Postpartum Depression: A Randomized Clinical Trial. *JAMA Psychiatry*. 2021;78(11):1200-1207. doi:10.1001/jamapsychiatry.2021.2488

378. van Schaik A, van Marwijk H, Adèr H, et al. Interpersonal Psychotherapy for Elderly Patients in Primary Care. *Am J Geriatr Psychiatry*. 2006;14(9):777-786. doi:10.1097/01.JGP.0000199341.25431.4b

379. Vazquez Gonzalez FL, Otero Otero P, Torres Iglesias A, Hermida GarcÌa E, Blanco Seoane V, DÌaz Fern·ndez O. A brief problem-solving indicated-prevention intervention for prevention

of depression in nonprofessional caregivers. *Psicothema*. 2013;25(1):87-92. doi:10.7334/psicothema2012.89

380. Vázquez FL, Torres Á, Otero P, Blanco V, Díaz O, Estévez LE. Analysis of the components of a cognitive-behavioral intervention administered via conference call for preventing depression among non-professional caregivers: A pilot study. *Aging Ment Health*. 2017;21(9):938-946. doi:10.1080/13607863.2016.1181714

381. Vázquez FL, López L, Torres Á J, et al. Analysis of the Components of a Cognitive-Behavioral Intervention for the prevention of Depression Administered via Conference Call to Nonprofessional Caregivers: A Randomized Controlled Trial. *Int J Env Res Public Health*. 2020;17(6). doi:10.3390/ijerph17062067

382. Verduyn C, Barrowclough C, Roberts J, Tarrier N, Harrington R. Maternal depression and child behaviour problems Randomised placebo-controlled trial of a cognitive-behavioural group intervention. *Br J Psychiatry*. 2003;183(OCT.):342-348. doi:10.1192/bjp.183.4.342

383. Vernmark K, Lenndin J, Bj‰rehed J, et al. Internet administered guided self-help versus individualized e-mail therapy: A randomized trial of two versions of CBT for major depression. *Behav Res Ther*. 2010;48(5):368-376. doi:10.1016/j.brat.2010.01.005

384. Vigod SN, Slyfield Cook G, Macdonald K, et al. Mother matters: Pilot randomized wait-list controlled trial of an online therapist-facilitated discussion board and support group for postpartum depression symptoms. *Depress Anxiety*. 2021;38(8):816-825. doi:10.1002/da.23163

385. Vitriol VG, Ballesteros ST, Florenzano RU, Weil KP, Benadof DF. Evaluation of an outpatient intervention for women with severe depression and a history of childhood trauma. *Psychiatr Serv*. 2009;60(7):936-942. doi:10.1176/appi.ps.60.7.936

386. Warmerdam L, Straten A, Twisk J, Riper H, Cuijpers P. Internet-based treatment for adults with depressive symptoms: randomized controlled trial. *J Med Internet Res*. 2008;10(4):e44. doi:10.2196/jmir.1094

387. Watkins ER, Taylor RS, Byng R, et al. Guided self-help concreteness training as an intervention for major depression in primary care: a Phase II randomized controlled trial. *Psychol Med*. 2012;42(7):1359-1371. doi:10.1017/s0033291711002480

388. Watt LM, Cappeliez P. Integrative and instrumental reminiscence therapies for depression in older adults: Intervention strategies and treatment effectiveness. *Aging Ment Health*. 2000;4(2):166-177. doi:10.1080/13607860050008691

389. Weissman MM, Prusoff BA, Dimascio A, Neu C, Goklaney M, Klerman GL. The efficacy of drugs and psychotherapy in the treatment of acute depressive episodes. *Am J Psychiatry*. 1979;136(4b):555-558. doi:10.1176/ajp.1979.136.4b.555

390. Westerhof GJ, Lamers SMA, Postel MG, Bohlmeijer ET. Online Therapy for Depressive Symptoms: An Evaluation of Counselor-Led and Peer-Supported Life Review Therapy. *The Gerontologist*. 2019;59(1):135-146. doi:10.1093/geront/gnx140

391. Wickberg B, Hwang CP. Counselling of postnatal depression: a controlled study on a population based Swedish sample. *J Affect Disord*. 1996;39(3):209-216. doi:10.1016/0165-0327(96)00034-1

392. Wiersma JE, Schaik DJF, Hoogendorn AW, et al. The effectiveness of the cognitive behavioral analysis system of psychotherapy for chronic depression: A randomized controlled trial. *Psychother Psychosom*. 2014;83(5):263-269. doi:10.1159/000360795

393. Wiklund I, Mohlkert P, Edman G. Evaluation of a brief cognitive intervention in patients with signs of postnatal depression: a randomized controlled trial. *Acta Obstet Gynecol Scand*. 2010;89(8):1100-1104. doi:10.3109/00016349.2010.500369

394. Williams JW, Barrett J, Oxman T, et al. Treatment of dysthymia and minor depression in primary care: A randomized controlled trial in older adults. *Jama*. 2000;284(12):1519-1526. doi:10.1001/jama.284.12.1519

395. Williams AD, Blackwell SE, Mackenzie A, Holmes EA, Andrews G. Combining imagination and reason in the treatment of depression: A randomized controlled trial of internet-based cognitive-bias modification and internet-CBT for depression. *J Consult Clin Psychol*. 2013;81(5):793-799. doi:10.1037/a0033247

396. Williams C, Wilson P, Morrison J, et al. Guided Self-Help Cognitive Behavioural Therapy for Depression in Primary Care: A Randomised Controlled Trial. *PLoS ONE*. 2013;8(1):e52735. doi:10.1371/journal.pone.0052735

397. Williams C, McClay CA, Matthews L, et al. Community-based group guided self-help intervention for low mood and stress: Randomised controlled trial. *Br J Psychiatry*. 2018;212(2):88-95. doi:10.1192/bjp.2017.18

398. Wilson PH, Goldin JC, Charbonneau-Powis M. Comparative efficacy of behavioral and cognitive treatments of depression. *Cogn Ther Res*. 1983;7(2):111-124. doi:10.1007/BF01190064

399. Wollersheim JP, Wilson GL. Group treatment of unipolar depression: A comparison of coping, supportive, bibliotherapy, and delayed treatment groups. *Prof Psychol Res Pract*. 1991;22(6):496. doi:10.1037/0735-7028.22.6.496

400. Wong DF. Cognitive and health-related outcomes of group cognitive behavioural treatment for people with depressive symptoms in Hong Kong: randomized wait-list control study. *Aust N Z J Psychiatry*. 2008;42(8):702-711. doi:10.1080/00048670802203418

401. Wong DF. Cognitive behavioral treatment groups for people with chronic depression in Hong Kong: a randomized wait-list control design. *Depress Anxiety*. 2008;25(2):142-148. doi:10.1002/da.20286

402. Wong SYS, Sun YY, Chan ATY, et al. Treating Subthreshold Depression in Primary Care: a Randomized Controlled Trial of Behavioral Activation With Mindfulness. *Ann Fam Med*. 2018;16(2):111-119. doi:10.1370/afm.2206

403. Wright JH, Wright AS, Albano AM, et al. Computer-assisted cognitive therapy for depression: maintaining efficacy while reducing therapist time. *Am J Psychiatry*. 2005;162(6):1158-1164. doi:10.1176/appi.ajp.162.6.1158

404. Wuthrich VM, Rapee RM. Randomised controlled trial of group cognitive behavioural therapy for comorbid anxiety and depression in older adults. *Behav Res Ther*. 2013;51(12):779-786. doi:10.1016/j.brat.2013.09.002

405. Wuthrich VM, Rapee RM, Kangas M, Perini S. Randomized controlled trial of group cognitive behavioral therapy compared to a discussion group for co-morbid anxiety and depression in older adults. *Psychol Med*. 2016;46(4):785-795. doi:10.1017/s0033291715002251

406. Xie J, He G, Ding S, et al. A randomized study on the effect of modified behavioral activation treatment for depressive symptoms in rural left-behind elderly. *Psychother Res J Soc Psychother Res*. 2019;29(3):372-382. doi:10.1080/10503307.2017.1364444

407. Yang X, Zhao J, Chen Y, Zu S, Zhao J. Comprehensive self-control training benefits depressed college students: a six-month randomized controlled intervention trial. *J Affect Disord*. 2018;226:251-260. doi:10.1016/j.jad.2017.10.014

408. Yeung A, Wang F, Feng F, et al. Outcomes of an online computerized cognitive behavioral treatment program for treating chinese patients with depression: A pilot study. *Asian J Psychiatry*. 2017;38:102-107. doi:10.1016/j.ajp.2017.11.007

409. Yuan J, Yin Y, Tang X, et al. Culturally adapted and lay-delivered cognitive behaviour therapy for older adults with depressive symptoms in rural China: a pilot trial. *Behav Cogn Psychother*. Published online 2020:1-5. doi:10.1017/S1352465820000818

410. Zemestani M, Mozaffari S. Acceptance and commitment therapy for the treatment of depression in persons with physical disability: a randomized controlled trial. *Clin Rehabil*. 2020;34(7):938-947. doi:10.1177/0269215520923135

411. Zemestani M, Davoodi I, Honarmand MM, Zargar Y, Ottaviani C. Comparative effects of group metacognitive therapy versus behavioural activation in moderately depressed students. *J Ment Health*. 2016;25(6):479-485. doi:10.3109/09638237.2015.1057326

412. Zemestani M, Fazeli Nikoo Z. Effectiveness of mindfulness-based cognitive therapy for comorbid depression and anxiety in pregnancy: a randomized controlled trial. *Arch Womens Ment Health*. 2019;23(2):207-214. doi:10.1007/s00737-019-00962-8

413. Zhao Y, Munro-Kramer ML, Shi S, Wang J, Zhao Q. Effects of antenatal depression screening and intervention among Chinese high-risk pregnant women with medically defined complications: A randomized controlled trial. *Early Interv Psychiatry*. 2019;13(5):1090-1098. doi:10.1111/eip.12731

414. Zhao Y, Lin Q, Wang J. An evaluation of a prenatal individualised mixed management intervention addressing breastfeeding outcomes and postpartum depression: A ramdomised controlled trial. *J Clin Nurs*. 2021;30(9-10):1347-1359. doi:10.1111/jocn.15684

415. Zu S, Xiang YT, Liu J, et al. A comparison of cognitive-behavioral therapy, antidepressants, their combination and standard treatment for Chinese patients with moderate–severe major depressive disorders. *J Affect Disord*. 2014;152-154:262-267. doi:10.1016/j.jad.2013.09.022

416. Higgins JPT, Altman DG, Gotzsche PC, et al. The Cochrane Collaboration’s tool for assessing risk of bias in randomised trials. *BMJ*. 2011;343(oct18 2):d5928-d5928. doi:10.1136/bmj.d5928

eTable 2 Summary characteristics of included primary studies

Summary characteristics of included effect sizes from primary studies.

**Characteristic**	***N* = 1,206**[1]
Target Group	
Adults	521 (43%)
General Medical	230 (19%)
Older Adults	114 (9.5%)
Other Target Groups	156 (13%)
Perinatal Depression	132 (11%)
Student Population	53 (4.4%)
Region	
Australia	82 (6.8%)
East Asia	79 (6.6%)
Europe	419 (35%)
North America	534 (44%)
Other Region	92 (7.6%)
Intervention	
CBT-Based	879 (73%)
Not-CBT-Based	327 (27%)
Format	
Group	326 (27%)
Guided Self-Help	282 (23%)
Individual	451 (37%)
Other Formats	147 (12%)
Control	
CAU	484 (40%)
Other Ctr	189 (16%)
WL	533 (44%)
diagnosis	
Cut-Off Score	497 (41%)
Diagnosis	652 (54%)
Subclinical Depression	57 (4.7%)
Risk of Bias	
High	21 (1.7%)
Low	403 (33%)
Some Concern	782 (65%)

[1]n (%)

eTable 3 Distribution of summary effect size estimates for Which factor: Control condition

**Summary effect sizes by control**				
**Control**	**Mean *g***	**Mean CI Lower**	**Mean CI Upper**	***k***
Wait List	0.66	0.348	0.96	836
All control conditions	0.55	0.294	0.81	1,872
Care as usual	0.52	0.224	0.82	1,194
Other control groups	0.53	0.086	0.98	379

Note: Mean Hedges´ g effect size information, including 95% Confidence Interval lower and upper bounds and number of included meta-analyses in the multiverse of meta-analyses.

eTable 4 Distribution of summary effect size estimates for Which factor: Diagnosis

Summary effect sizes by Which factor: Diagnosis

**Diagnosis**	**Mean *g***	**Mean CI Lower**	**Mean CI Upper**	***k***
Subclinical depression	0.57	0.049	1.10	84
Cut-off score	0.57	0.226	0.90	1,045
Diagnosis	0.56	0.276	0.85	1,224
All diagnoses	0.56	0.292	0.83	1,928

Note: Mean Hedges´ g effect size information, including 95% Confidence Interval lower and upper bounds and number of included meta-analyses in the multiverse of meta-analyses.

Plessen CY, *et al. BMJ Ment Health* 2023; 26:e300626. doi: 10.1136/bmjment-2022-300626

eTable 5 Distribution of summary effect size estimates for Which factor: Format

Summary effect sizes by Which factor: therapy format

Format	Mean *g*	Mean CI Lower	Mean CI Upper	*k*
Group	0.76	0.32	1.19	536
Other formats	0.56	0.22	0.90	340
All formats	0.56	0.30	0.81	1,823
Individual	0.51	0.19	0.82	996
Guided self-help	0.50	0.27	0.72	586

Note: Mean Hedges´ g effect size information, including 95% Confidence Interval lower and upper bounds and number of included meta-analyses in the multiverse of meta-analyses.

eTable 6 Distribution of summary effect size estimates for Which factor: Method

Summary effect sizes by meta-analytical method

**Method**	**Mean *g***	**Mean CI Lower**	**Mean CI Upper**	***k***
3-LEVEL	0.66	0.36	0.96	963
RVE	0.66	0.27	1.05	963
REML	0.64	0.42	0.86	591
P-UNIFORM	0.60	0.29	0.89	582
FE	0.52	0.44	0.60	591
PET-PEESE	0.18	-0.24	0.59	591

Note: Mean Hedges´ g effect size information, including 95% Confidence Interval lower and upper bounds and number of included meta-analyses in the multiverse of meta-analyses.

Plessen CY, *et al. BMJ Ment Health* 2023; 26:e300626. doi: 10.1136/bmjment-2022-300626

eTable 7 Distribution of summary effect size estimates for Which factor: Type

**Summary effect sizes by type**				
**Type**	**Mean *g***	**Mean CI Lower**	**Mean CI Upper**	**k**
CBT-based	0.60	0.29	0.91	1,620
All types	0.56	0.27	0.84	1,928
Not-CBT-based	0.48	0.19	0.78	733

Note: Mean Hedges´ g effect size information, including 95% Confidence Interval lower and upper bounds and number of included meta-analyses in the multiverse of meta-analyses.

Plessen CY, *et al. BMJ Ment Health* 2023; 26:e300626. doi: 10.1136/bmjment-2022-300626

eTable 8 Distribution of summary effect size estimates for Which factor: Risk of bias

**Summary effect sizes by risk of bias**				
**Risk of Bias**	**Mean *g***	**Mean CI Lower**	**Mean CI Upper**	***k***
Exclude high risk of bias studies	0.61	0.27	0.95	2,413
Include all studies	0.56	0.35	0.77	834
Include only low risk of bias studies	0.45	0.19	0.72	1,034

Note: Mean Hedges´ g effect size information, including 95% Confidence Interval lower and upper bounds and number of included meta-analyses in the multiverse of meta-analyses.

Plessen CY, *et al. BMJ Ment Health* 2023; 26:e300626. doi: 10.1136/bmjment-2022-300626

eTable 9 Distribution of summary effect size estimates for Which factor: Target Group

**Summary effect sizes by target group**				
**Target group**	**Mean *g***	**Mean CI Lower**	**Mean CI Upper**	***k***
Student population	0.82	0.51	1.12	54
Other target groups	0.69	0.19	1.18	325
Older adults	0.64	0.23	1.05	295
Perinatal depression	0.60	0.27	0.92	286
General medical	0.58	0.18	0.97	475
All groups	0.54	0.31	0.78	1,770
Adults	0.51	0.26	0.75	1,076

Note: Mean Hedges´ *g* effect size information, including 95% Confidence Interval lower and upper bounds and number of included meta-analyses in the multiverse of meta-analyses.

eTable 10 Sensitivity analyses for multiverse size

Mean summary effect sizes for different numbers of included primary studies.

**Number of Specifications**	***k***	**Mean *g***	**95% CI LB**	**95% CI UB**	**Minimum**	**Maximum**	**25% Quantile**	**75% Quantile**
k > 10	4281	0.56	0.27	0.86	-0.66	2.5	0.42	0.71
k > 25	2138	0.59	0.39	0.79	-0.44	1.4	0.46	0.71
k > 50	1110	0.62	0.46	0.78	-0.22	1.4	0.50	0.72

Notes. *k* = number of included meta-analyses; Mean g = mean Hedges' g of all included meta-analyses; 95% CI LB = Lower bound of 95% confidence interval; 95% CI UB = Upper bound of 95% confidence interval; Minimum = smallest summary effect size estimate; Maximum = largest summary effect size estimate; 25% Quantile = 25% of studies have a Hedges g equal or lower than this value; 75% Quantile = 75% of studies have a Hedges *g* equal or lower than this value, 50% of all summary effect size estimates are between these two quantiles

Plessen CY, *et al. BMJ Ment Health* 2023; 26:e300626. doi: 10.1136/bmjment-2022-300626

eFigure 1 Average effect size estimates for Which factor: Control condition

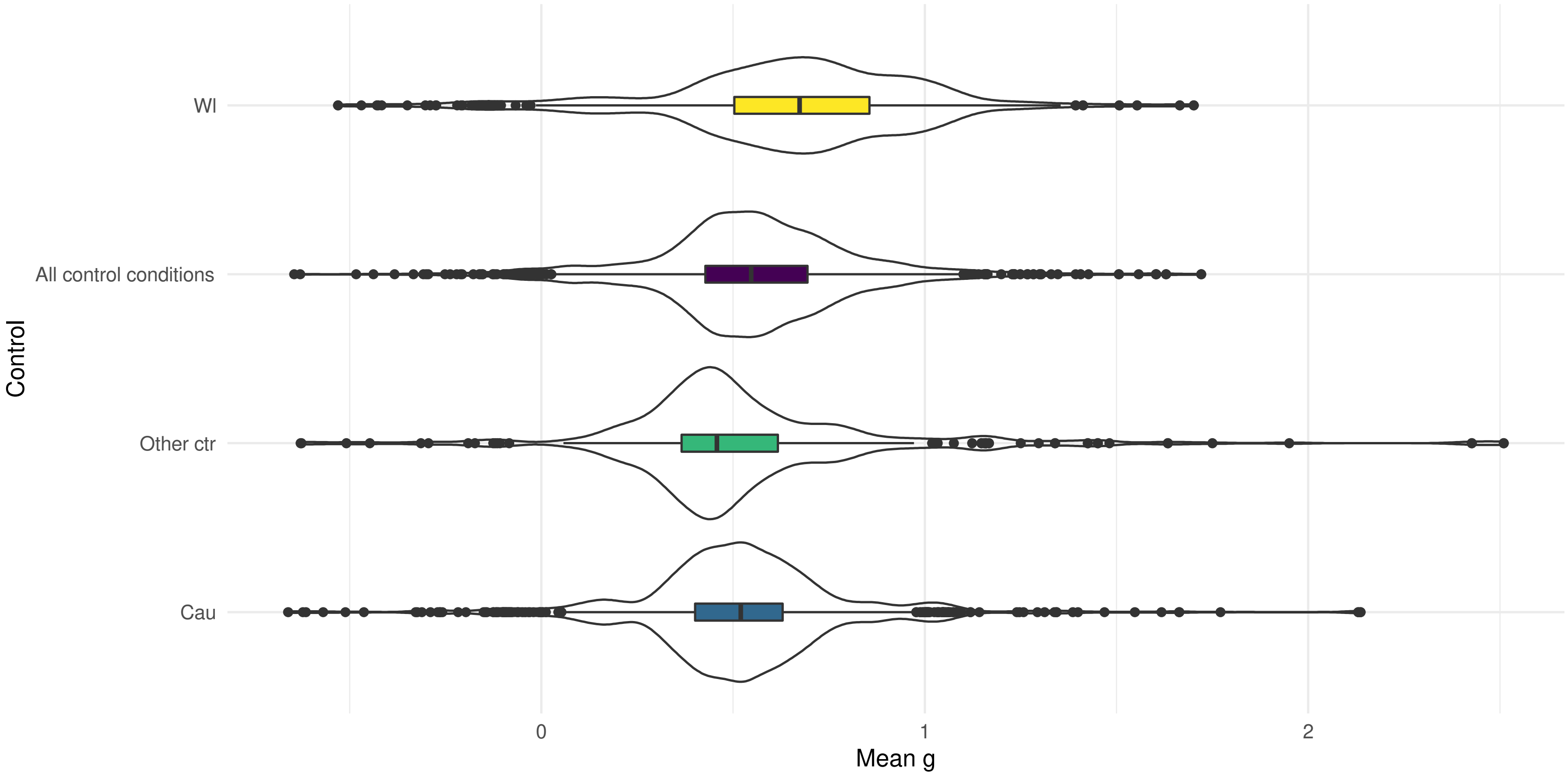

eFigure 2 Average effect size estimates for Which factor: Diagnosis

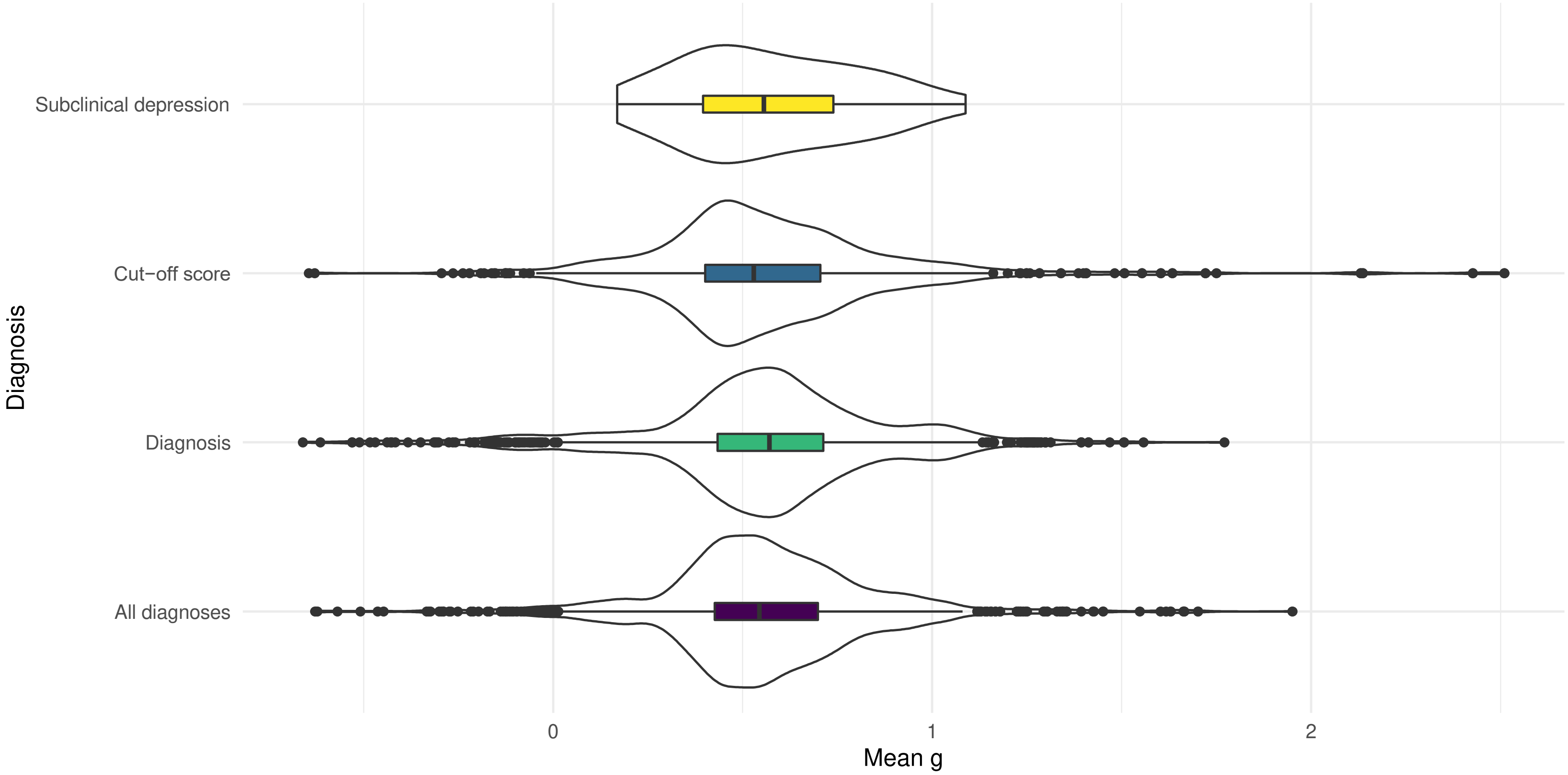

eFigure 3 Average effect size estimates for Which factor: Format

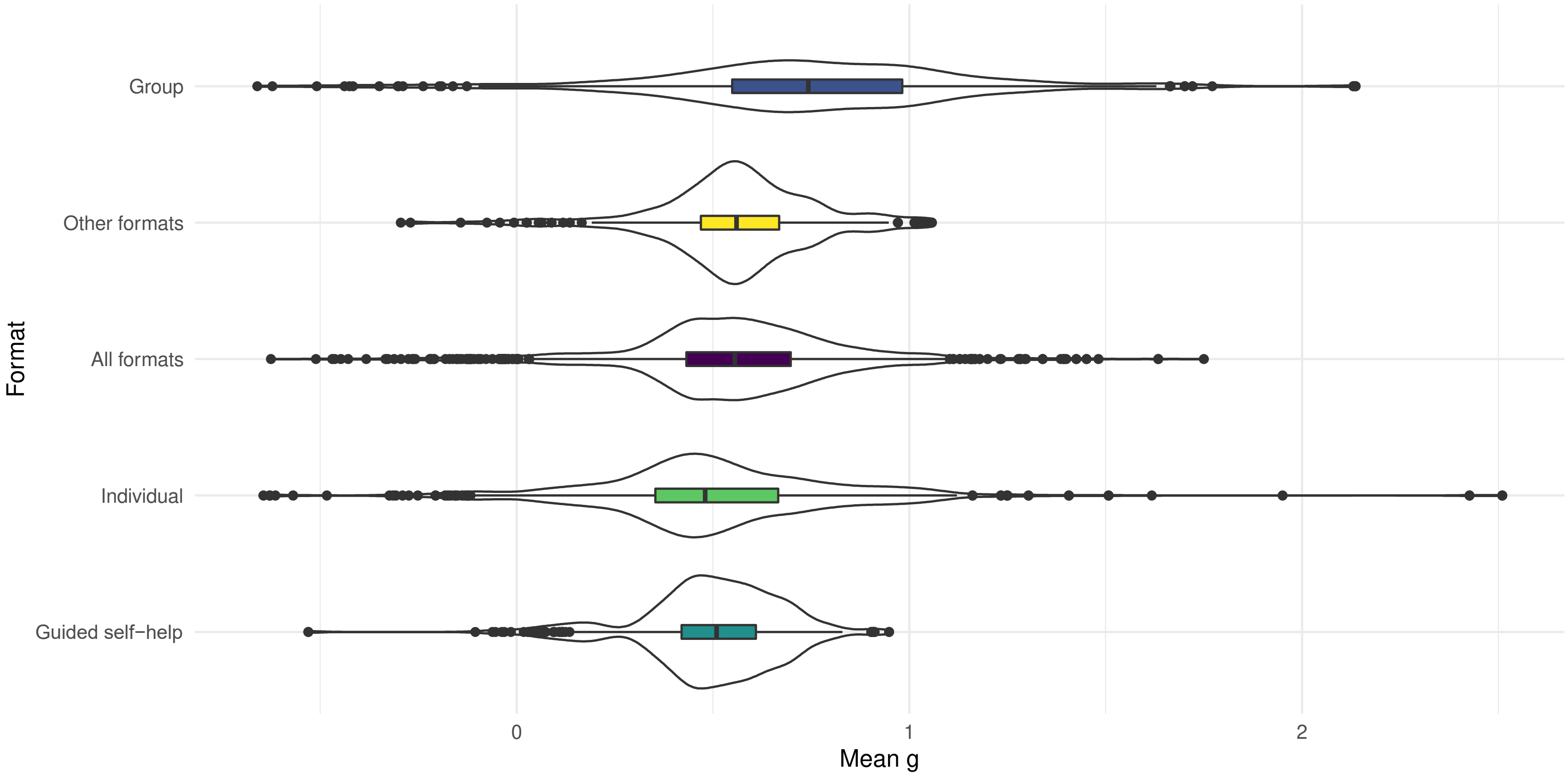

eFigure 4 Average effect size estimates for Which factor: Method

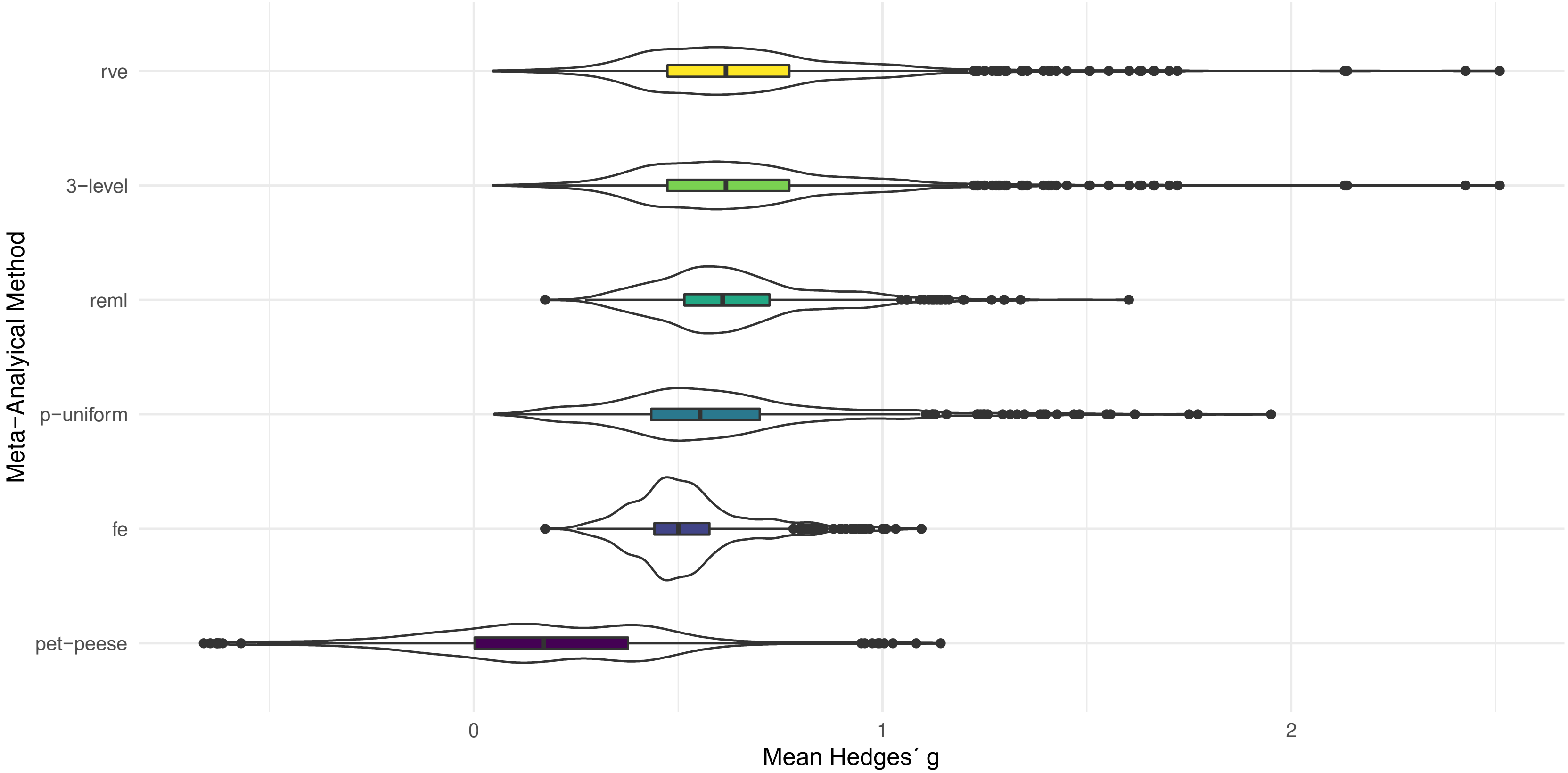

Plessen CY, *et al. BMJ Ment Health* 2023; 26:e300626. doi: 10.1136/bmjment-2022-300626

eFigure 5 Average effect size estimates for Which factor: Type

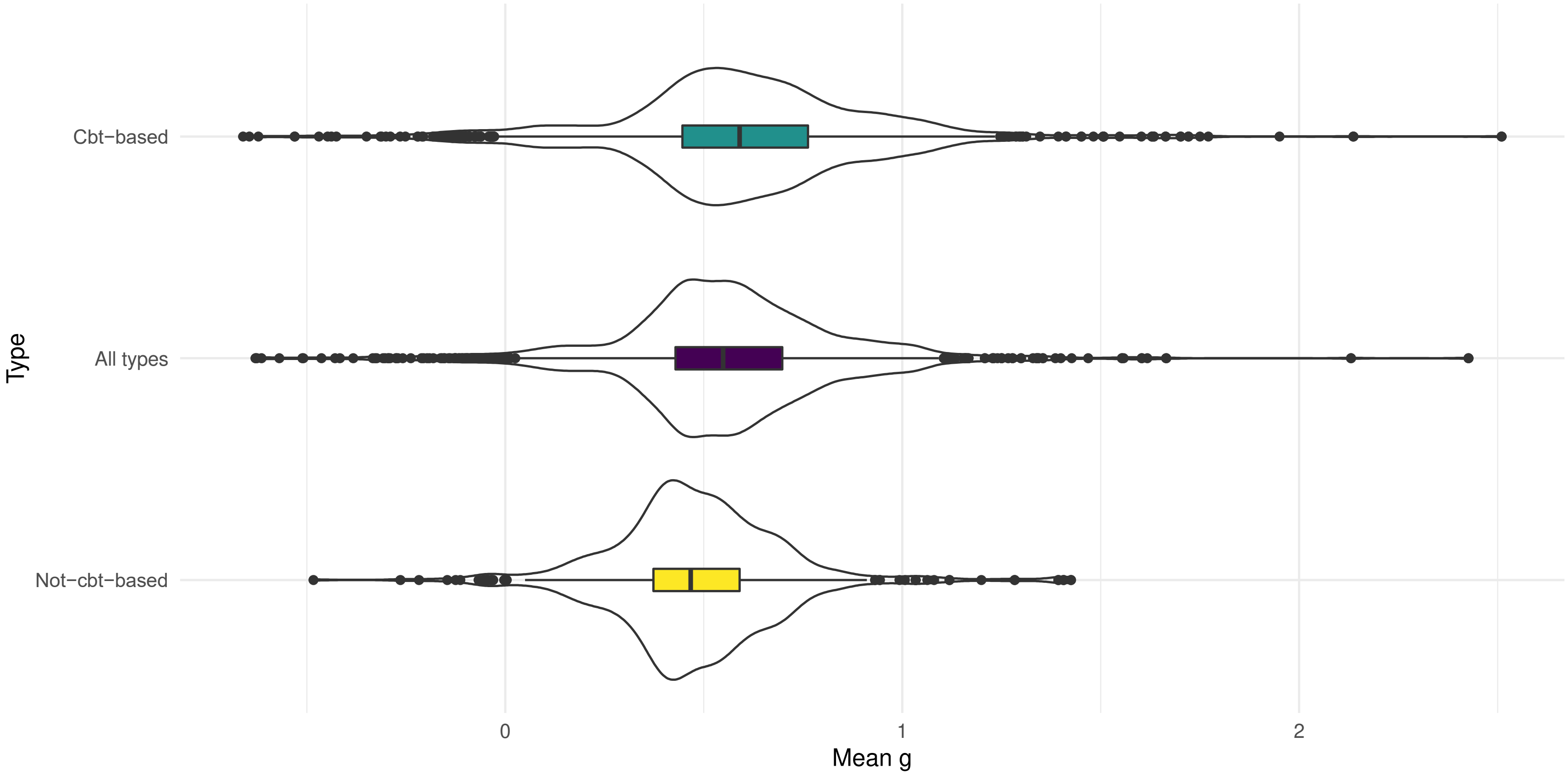

eFigure 6 Average effect size estimates for Which factor: Risk of bias

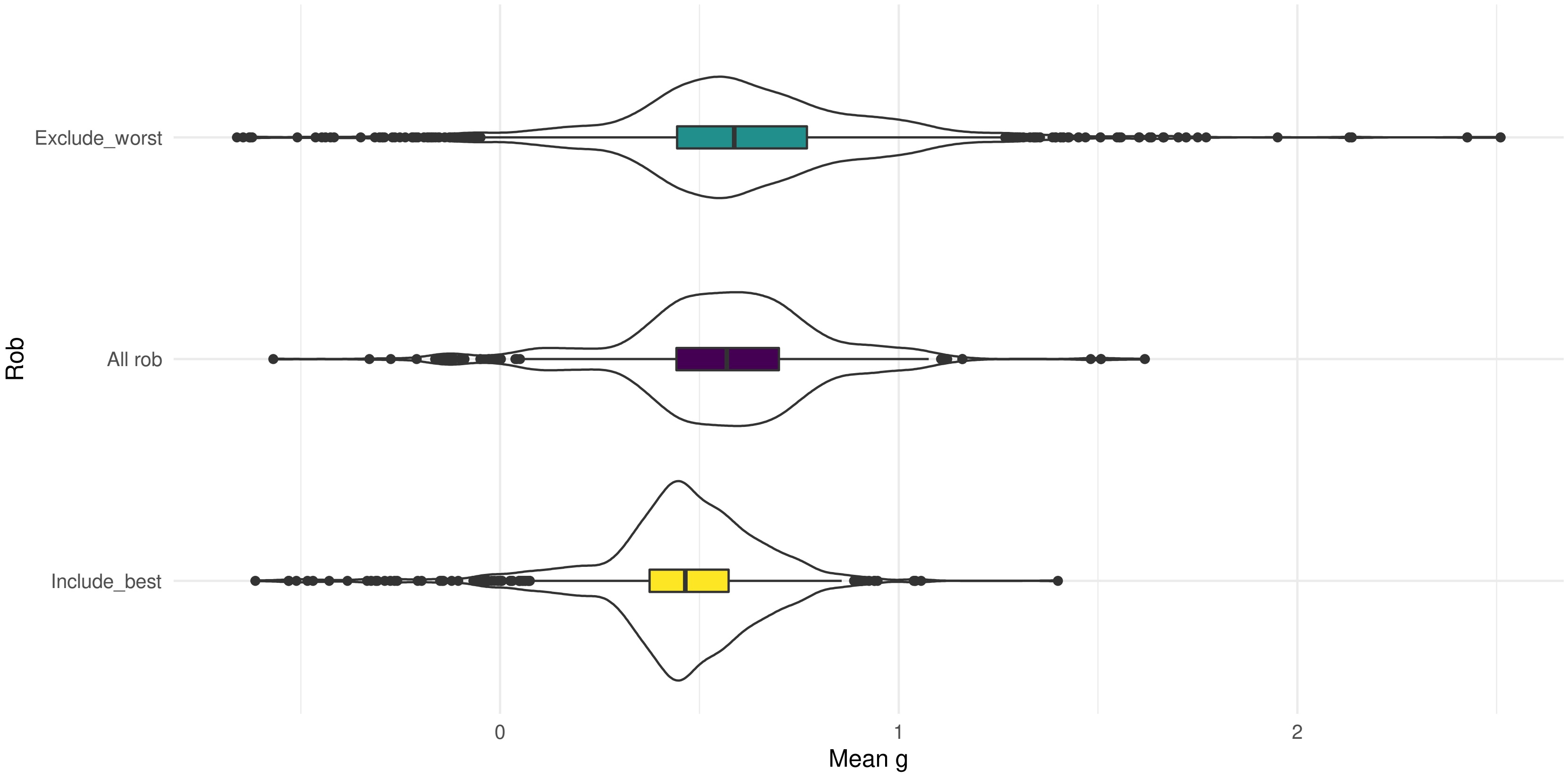

eFigure 7 Average effect size estimates for Which factor: Target Group

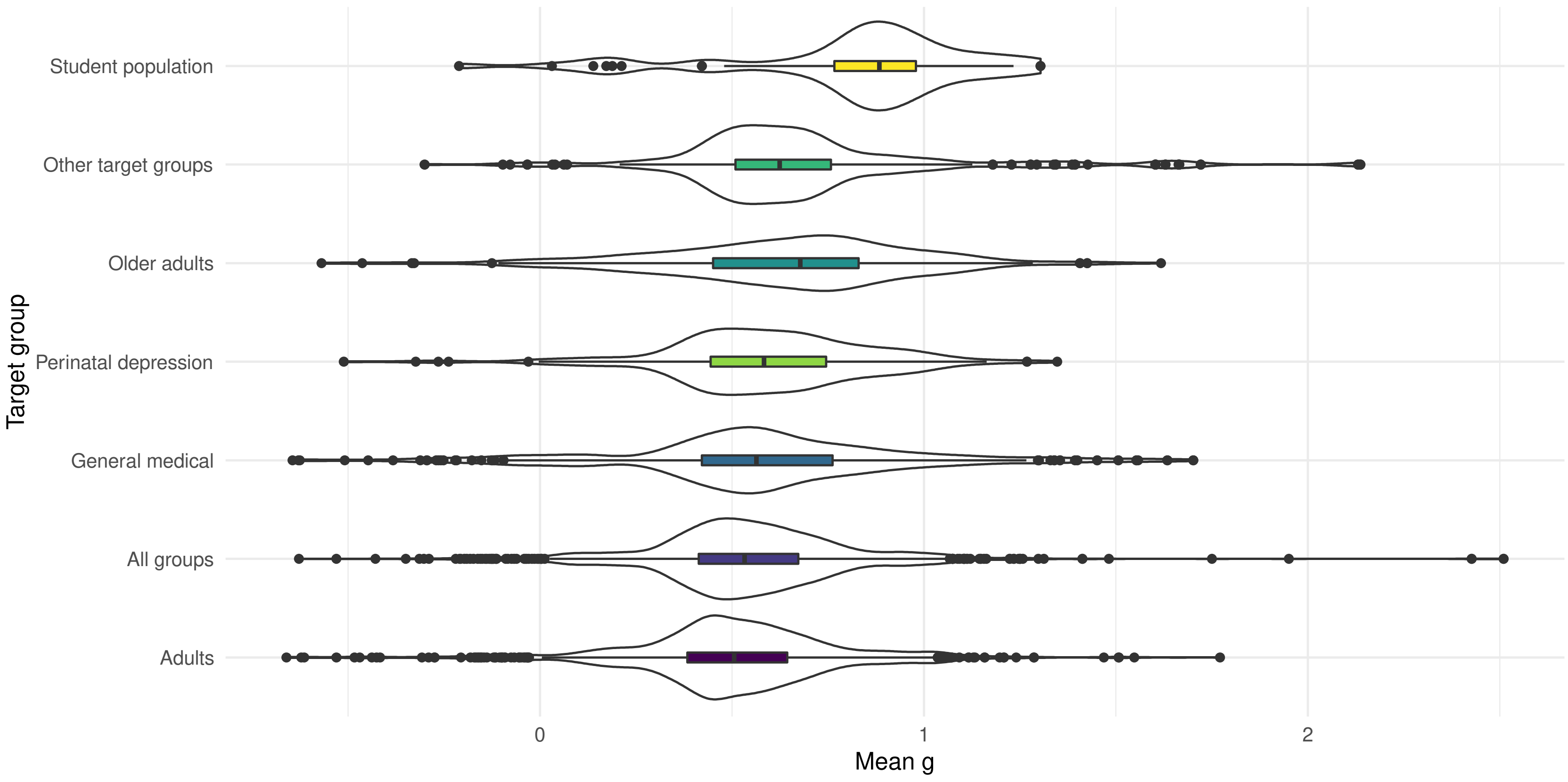

BMJ Publishing Group Limited (BMJ) disclaims all liability and responsibility arising from any reliance placed on this supplemental material which has been supplied by the author(s)

eFigure 8 Inferential specification curve plot against a simulated clinically relevant effect size

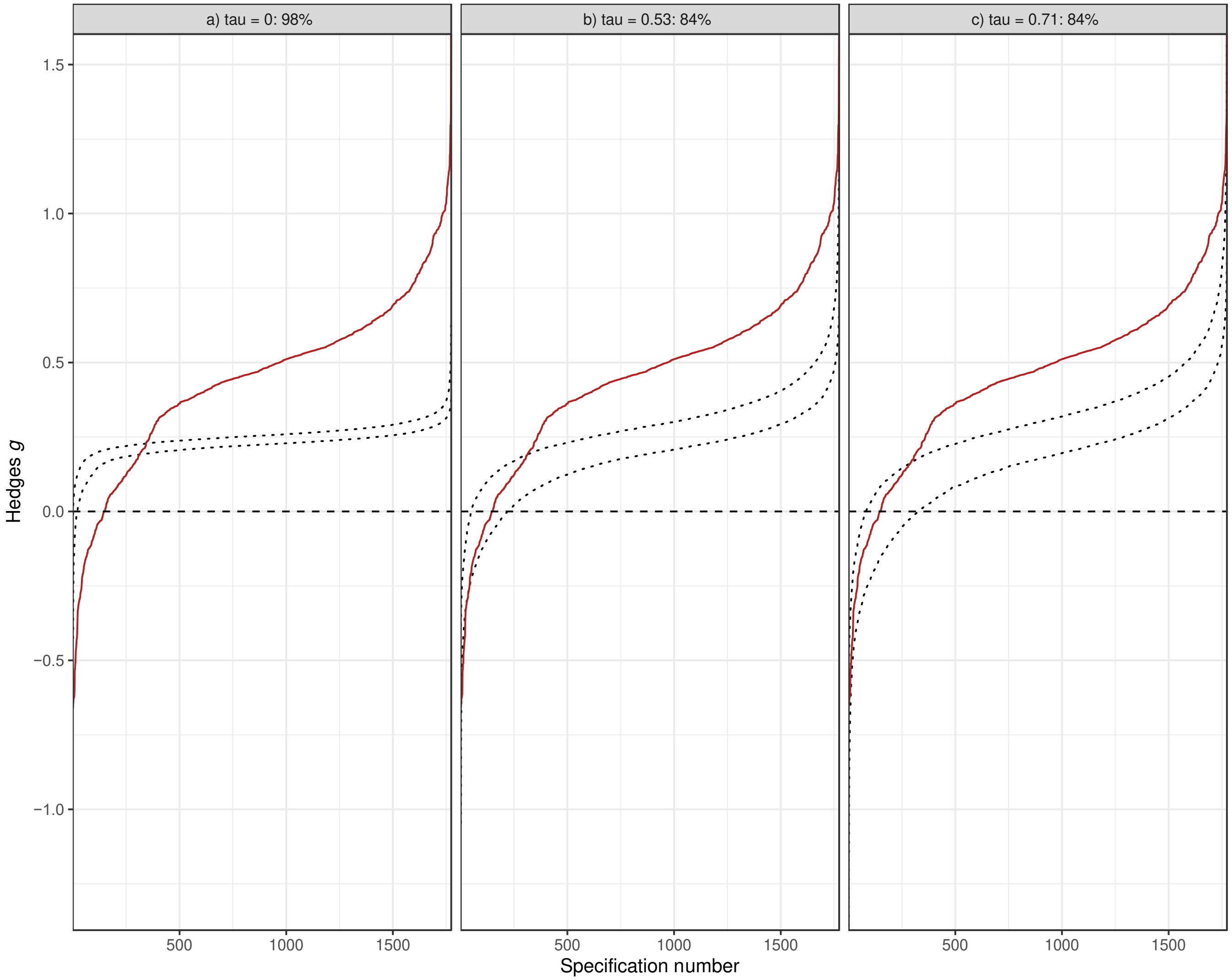

Plessen CY, *et al. BMJ Ment Health* 2023; 26:e300626. doi: 10.1136/bmjment-2022-300626

BMJ Publishing Group Limited (BMJ) disclaims all liability and responsibility arising from any reliance
placed on this supplemental material which has been supplied by the author(s)

eFigure 9 Sensitivity analyses for multiverse size

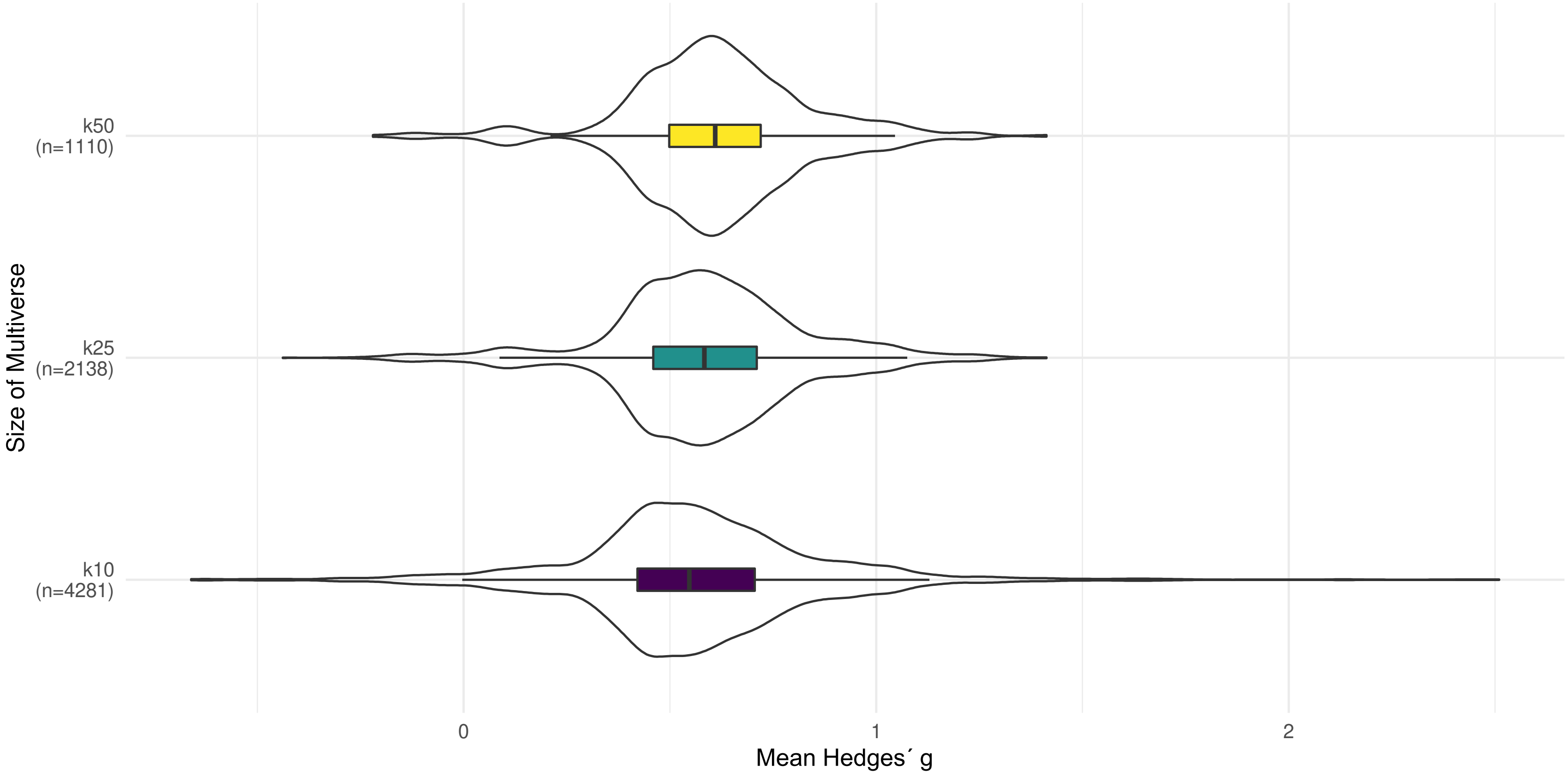

Plessen CY, *et al. BMJ Ment Health* 2023; 26:e300626. doi: 10.1136/bmjment-2022-300626

eMethods

eMethods 1. Search strings for PubMed.

Search strings for four bibliographical databases.

PubMed

Psychotherapy [MH] OR psychotherap*[All Fields] OR cbt[All Fields] OR "behavior therapies"[All Fields] OR
"behavior therapy"[All Fields] OR "behavior therapeutic"[All Fields] OR "behavior therapeutical"[All Fields] OR
"behavior therapeutics"[All Fields] OR "behavior therapeutist"[all Fields] OR "behavior therapeutists"[All Fields] OR
"behavior treatment"[All Fields] OR "behavior treatments"[All Fields] OR "behaviors therapies"[All Fields] OR
"behaviors therapy"[All Fields] OR "behaviors therapeutics"[All Fields] OR "behaviors therapeutic"[All Fields] OR
"behaviors therapeutical"[All Fields] OR "behaviors therapeutist"[All Fields] OR "behaviors therapeutists"[All Fields]
OR "behaviors treatment"[All Fields] OR "behaviors treatments"[All Fields] OR "behavioral therapies"[All Fields] OR
"behavioral therapy"[All Fields] OR "behavioral therapeutics"[All Fields] OR "behavioral therapeutic"[All Fields] OR
"behavioral therapeutical"[All Fields] OR "behavioral therapeutist"[All Fields] OR "behavioral therapeutists"[All
Fields] OR "behavioral treatment"[All Fields] OR "behavioral treatments"[All Fields] OR "behaviour therapies"[All
Fields] OR "behaviour therapy"[All Fields] OR "behaviour therapeutic"[All Fields] OR "behaviour therapeutical"[All
Fields] OR "behaviour therapeutics"[All Fields] OR "behaviour therapeutist"[all Fields] OR "behaviour therapeutists"[All Fields] OR "behaviour treatment"[All Fields] OR "behaviour treatments"[All Fields] OR "behaviours
therapies"[All Fields] OR "behaviours therapy"[All Fields] OR "behaviours therapeutics"[All Fields] OR "behaviours
therapeutic"[All Fields] OR "behaviours therapeutical"[All Fields] OR "behaviours therapeutist"[All Fields] OR
"behaviours therapeutists"[All Fields] OR "behaviours treatment"[All Fields] OR "behaviours treatments"[All Fields]
OR "behavioural therapies"[All Fields] OR "behavioural therapy"[All Fields] OR "behavioural therapeutics"[All Fields]
OR "behavioural therapeutic"[All Fields] OR "behavioural therapeutical"[All Fields] OR "behavioural therapeutist"[All
Fields] OR "behavioural therapeutists"[All Fields] OR "behavioural treatment"[All Fields] OR "behavioural
treatments"[All Fields] OR "cognition therapies"[All Fields] OR "cognition therapie"[All Fields] OR "cognition
therapy"[All Fields] OR "cognition therapeutical"[All Fields] OR "cognition therapeutic"[All Fields] OR "cognition
therapeutics"[All Fields] OR "cognition therapeutist"[All Fields] OR "cognition therapeutists"[All Fields] OR
"cognition treatment"[All Fields] OR "cognition treatments"[All Fields] OR psychodynamic[All Fields] OR
Psychoanalysis[MH] OR psychoanalysis[All Fields] OR psychoanalytic*[All Fields] OR counselling[All Fields] OR
counseling[All Fields] OR Counseling[MH] OR "problem-solving"[All Fields] OR mindfulness[All Fields] OR

Plessen CY, *et al. BMJ Ment Health* 2023; 26:e300626. doi: 10.1136/bmjment-2022-300626

(acceptance[All Fields] AND commitment[All Fields]) OR "assertiveness training"[All Fields] OR "behavior
activation"[All Fields] OR "behaviors activation"[All Fields] OR "behavioral activation"[All Fields] OR "cognitive
therapies"[All Fields] OR "cognitive therapy"[All Fields] OR "cognitive therapeutic"[All Fields] OR "cognitive
therapeutics"[All Fields] OR "cognitive therapeutical"[All Fields] OR "cognitive therapeutist"[All Fields] OR
"cognitive therapeutists"[All Fields] OR "cognitive treatment"[All Fields] OR "cognitive treatments"[All Fields] OR
"cognitive restructuring"[All Fields] OR (("compassion-focused"[All Fields] OR "compassion-focussed"[All Fields])
AND (therapy[SH] OR therapies[All Fields] OR therapy[All Fields] OR therape*[All Fields] OR therapis*[All
Fields]OR Therapeutics [OR treatment*[All Fields])) OR ((therapy[SH] OR therapies[All Fields]
OR therapy [All Fields] OR therape*[All Fields] OR therapis*[All Fields] OR Therapeutics[MH] OR treatment*[All
Fields]) AND constructivist*[All Fields]) OR "metacognitive therapies"[All Fields] OR "metacognitive therapy"[All
Fields] OR "metacognitive therapeutic"[All Fields] OR "metacognitive therapeutics"[All Fields] OR "metacognitive
therapeutical"[All Fields] OR "metacognitive therapeutist"[All Fields] OR "metacognitive therapeutists"[All Fields] OR
"metacognitive treatment"[All Fields] OR "metacognitive treatments"[All Fields] OR "meta-cognitive therapies"[All
Fields] OR "meta-cognitive therapy"[All Fields] OR "meta-cognitive therapeutic"[All Fields] OR "meta-cognitive
therapeutics"[All Fields] OR "meta-cognitive therapeutical"[All Fields] OR "meta-cognitive therapeutist"[All Fields]
OR "meta-cognitive therapeutists"[All Fields] OR "meta-cognitive treatment"[All Fields] OR "meta-cognitive
treatments"[All Fields] OR "solution-focused therapies"[All Fields] OR "solution-focused therapy"[All Fields] OR
"solution-focused therapeutic"[All Fields] OR "solution-focused therapeutics"[All Fields] OR "solution-focused
therapeutical"[All Fields] OR "solution focused therapies"[All Fields] OR "solution focused therapy"[All Fields] OR
"solution focused therapeutic"[All Fields] OR "solution focused therapeutics"[All Fields] OR "solution focused
therapeutical"[All Fields]OR "solution-focussed therapies"[All Fields] OR "solution-focussed therapy"[All Fields] OR
"solution-focussed therapeutic"[All Fields] OR "solution-focussed therapeutics"[All Fields] OR "solution-focussed
therapeutical"[All Fields]OR "solution focussed therapies"[All Fields] OR "solution focussed therapy"[All Fields] OR
"solution focussed therapeutic"[All Fields] OR "solution focussed therapeutics"[All Fields] OR "solution focussed
therapeutical"[All Fields] OR "self-control therapies"[All Fields] OR "self-control therapy"[All Fields] OR "self-control
therapeutics"[All Fields] OR "self-control therapeutical"[All Fields] OR "self-control therapeutic"[All Fields] OR "self-
control training"[All Fields] OR "self-control trainings"[All Fields] OR "self control therapies"[All Fields] OR "self
control therapy"[All Fields] OR "self control therapeutics"[All Fields] OR "self control therapeutical"[All Fields] OR
"self control therapeutic"[All Fields] OR "self control training"[All Fields] OR "self control trainings"[All Fields]
AND
(Depressive Disorder[MH] OR Depression[MH]OR dysthymi*[All Fields] OR "affective disorder"[All Fields]OR

"affective disorders"[All Fields] OR "mood disorder"[All Fields] OR "mood disorders"[All Fields] OR depression*[All
Fields] OR depressive*[All Fields] OR "dysthymic disorder"[MeSH Terms])
Limits: RCTs

Embase
#1
'psychotherapy'/exp OR 'psychotherapy' OR 'psychotherapies' OR 'psychotherapeutics' OR 'psychotherapeutical' OR
'cognitive therapy'/exp OR 'cognitive behavior therapy'/exp OR 'behavior therapy'/exp OR ́cbt ́ OR ́cognitive
behavioural therapy ́ OR ́cognitive behavioural therapies ́ OR cognitive behavioral therapy ́ OR 'cognitive behavioral
therapies' OR 'behavior therapy' OR 'behavior therapies' OR 'behaviour therapy' OR 'behaviour therapies' OR 'cognition
therapy' OR 'cognitive therapies' OR 'cognitive therapy' OR 'cognitive therapeutic' OR 'cognitive therapeutics' OR
'cognitive therapeutical' OR 'cognitive therapeutist' OR 'cognitive therapeutists' OR 'cognitive treatment' OR 'cognitive
treatments' OR 'cognitive restructuring' OR 'cognition therapies' OR 'cognition therapie' OR 'cognition therapeutical' OR
'cognition therapeutic' OR 'cognition therapeutics' OR 'cognition therapeutist' OR 'cognition therapeutists' OR 'cognition
treatment' OR 'cognition treatments' OR 'behavior therapeutic' OR 'behavior therapeutical' OR 'behavior therapeutics'
OR 'behavior therapeutist' OR 'behavior therapeutists' OR 'behavior treatment' OR 'behavior treatments' OR 'behaviors
therapies' OR 'behaviors therapy' OR 'behaviors therapeutics' OR 'behaviors therapeutic' OR 'behaviors therapeutical'
OR 'behaviors therapeutist' OR 'behaviors therapeutists' OR 'behaviors treatment' OR 'behaviors treatments' OR
'behavioral therapies' OR 'behavioral therapy' OR 'behavioral therapeutics' OR 'behavioral therapeutic' OR 'behavioral
therapeutical' OR 'behavioral therapeutist' OR 'behavioral therapeutists' OR 'behavioral treatment' OR 'behavioral
treatments' OR 'behaviour therapeutic' OR 'behaviour therapeutical' OR 'behaviour therapeutics' OR 'behaviour
therapeutist' OR 'behaviour therapeutists' OR 'behaviour treatment' OR 'behaviour treatments' OR 'behaviours therapies'
OR 'behaviours therapy' OR 'behaviours therapeutics' OR 'behaviours therapeutic' OR 'behaviours therapeutical' OR
'behaviours therapeutist' OR 'behaviours therapeutists' OR 'behaviours treatment' OR 'behaviours treatments' OR
'behavioural therapies' OR 'behavioural therapy' OR 'behavioural therapeutics' OR 'behavioural therapeutic' OR
'behavioural therapeutical' OR 'behavioural therapeutist' OR 'behavioural therapeutists' OR 'behavioural treatment' OR
'behavioural treatments' OR 'behavior activation' OR 'behaviors activation' OR 'behavioral activation' OR 'behaviour
activation' OR 'behaviours activation' OR 'behavioural activation' OR 'psychoanalytic therapy'/exp OR 'psychodynamic'
OR 'psychodynamical' OR 'psychoanalysis' OR 'psychoanalytical' OR 'counselling'/exp OR 'counseling'/exp OR
'counselling' OR 'counseling' OR 'problem-solving' OR 'problem solving' OR 'supportive therapy' OR 'metacognitive
therapy' OR 'metacognitive therapies' OR 'metacognitive therapeutic' OR 'metacognitive therapeutics' OR
'metacognitive therapeutical' OR 'metacognitive therapeutist' OR 'metacognitive therapeutists' OR 'metacognitive
treatment' OR 'metacognitive treatments' OR 'meta-cognitive therapy' OR 'meta-cognitive therapies' OR

'meta-cognitive
therapeutic' OR 'meta-cognitive therapeutics' OR 'meta-cognitive therapeutical' OR 'meta-cognitive therapeutist' OR
'meta-cognitive therapeutists' OR 'meta-cognitive treatment' OR 'meta-cognitive treatments' OR 'solution-focused
therapies' OR 'solution focused therapies' OR 'solution-focussed therapies' OR 'solution focused therapies' OR 'solution-
focused therapy' OR 'solution focused therapy' OR 'solution-focussed therapy' OR 'solution focused therapy' OR
'solution-focused therapeutic' OR 'solution focused therapeutic' OR 'solution-focussed therapeutic' OR 'solution
focussed therapeutic' OR 'solution-focused therapeutics' OR 'solution focused therapeutics' OR 'solution-focussed
therapeutics' OR 'solution focused therapeutics' OR 'solution-focused therapeutical' OR 'solution focused therapeutical'
OR 'solution-focussed therapeutical' OR 'solution focused therapeutical' OR 'self-control therapies' OR 'self control
therapies' OR 'self-control therapy' OR 'self control therapy' OR 'self-control therapeutics' OR 'self control therapeutics'
OR 'self-control therapeutical' OR 'self control therapeutical' OR 'self-control therapeutic' OR 'self control therapeutic'
OR 'self-control training' OR 'self control training' OR 'self control trainings' OR 'self-control trainings' OR
'mindfulness' OR 'acceptance commitment' OR 'acceptance and commitment' OR 'assertiveness training'
#2
'compassion-focused' OR 'compassion-focussed' OR 'compassion focused' OR 'compassion focussed' OR 'constructivist'
OR 'constructivists'
#3
'therapies' OR 'therapy' OR 'therapeutics' OR 'therapist' OR 'treatment' OR 'treatments'
#4
Combine: #2 AND #3
#5: #1 OR #4
#6
'depressive disorder'/exp OR 'depression'/exp OR 'depressive' OR 'major depression'/exp OR 'major depressive
disorder'/exp OR 'depression' OR 'depressions' OR 'depressive' OR 'dysthymic disorder'/exp OR 'dysthymic disorder'
OR 'dysthymia'/exp OR 'dysthymic' OR 'mood disorder'/exp OR 'affective disorder'/exp OR 'affective disorder' OR
'affective disorders' OR 'mood disorder' OR 'mood disorders'
Combine: #5 AND #6
Limits: RCTs
PsycINFO
(DE "Psychotherapy" OR "Psychotherapy" OR "psychotherapies" OR "psychotherapeutic" OR "psychotherapeutical"
OR "psychotherapeutics" OR DE "Behavior Therapy" OR DE "Cognitive Behavior Therapy" OR "CBT" OR "behavior
therapies" OR "behavior therapy" OR "behavior therapeutic" OR "behavior therapeutical" OR "behavior therapeutics"
OR "behavior therapeutist" OR "behavior therapeutists" OR "behavior treatment" OR "behavior treatments" OR
"behaviors therapies" OR "behaviors therapy" OR "behaviors therapeutics" OR "behaviors therapeutic" OR "behaviors
therapeutical" OR "behaviors therapeutist" OR "behaviors therapeutists" OR "behaviors treatment" OR "behaviors
treatments" OR "behavioral therapies" OR "behavioral therapy" OR "behavioral therapeutics" OR "behavioral
therapeutic" OR "behavioral therapeutical" OR "behavioral therapeutist" OR "behavioral therapeutists" OR "behavioral

treatment" OR "behavioral treatments" OR "behaviour therapies" OR "behaviour therapy" OR "behaviour therapeutic"
OR "behaviour therapeutical" OR "behaviour therapeutics" OR "behaviour therapeutist" OR "behaviour therapeutists"
OR "behaviour treatment" OR "behaviour treatments" OR "behaviours therapies" OR "behaviours therapy" OR
"behaviours therapeutics" OR "behaviours therapeutic" OR "behaviours therapeutical" OR "behaviours therapeutist"
OR "behaviours therapeutists" OR "behaviours treatment" OR "behaviours treatments" OR "behavioural therapies" OR
"behavioural therapy" OR "behavioural therapeutics" OR "behavioural therapeutic" OR "behavioural therapeutical" OR
"behavioural therapeutist" OR "behavioural therapeutists" OR "behavioural treatment" OR "behavioural treatments" OR
"cognition therapies" OR "cognition therapie" OR "cognition therapy" OR "cognition therapeutical" OR "cognition
therapeutic" OR "cognition therapeutics" OR "cognition therapeutist" OR "cognition therapeutists" OR "cognition
treatment" OR "cognition treatments" OR "cognitive therapies" OR "cognitive therapy" OR "cognitive therapeutic" OR
"cognitive therapeutics" OR "cognitive therapeutical" OR "cognitive therapeutist" OR "cognitive therapeutists" OR
"cognitive treatment" OR "cognitive treatments" OR "cognitive restructuring" OR DE "Emotion Focused Therapy" OR
DE "Psychoanalysis" OR "psychoanalysis" OR "psychoanalytic" OR "psychoanalytical "OR DE "Psychodynamic
Psychotherapy" OR "psychodynamic" OR DE "Psychotherapeutic Counseling" OR "counselling" OR "counseling" OR
"problem-solving" OR "problem solving" OR "mindfulness" OR ("acceptance" AND "commitment") OR "assertiveness
training" OR "behavior activation" OR "behaviors activation" OR "behavioral activation" OR "behaviour activation"
OR "behaviours activation" OR "behavioural activation" OR "metacognitive therapies" OR "metacognitive therapy" OR
"metacognitive therapeutic" OR "metacognitive therapeutics" OR "metacognitive therapeutical" OR "metacognitive
therapeutist" OR "metacognitive therapeutists" OR "metacognitive treatment" OR "metacognitive treatments" OR
"meta-cognitive therapies" OR "meta-cognitive therapy" OR "meta-cognitive therapeutic" OR "meta-cognitive
therapeutics" OR "meta-cognitive therapeutical" OR "meta-cognitive therapeutist" OR "meta-cognitive therapeutists"
OR "meta-cognitive treatment" OR "meta-cognitive treatments" OR DE "Solution Focused Therapy" OR "solution-
focused therapies" OR "solution-focused therapy" OR "solution-focused therapeutic" OR "solution-focused
therapeutics" OR "solution-focused therapeutical" OR "solution-focussed therapies" OR "solution-focussed therapy"
OR "solution-focussed therapeutic" OR "solution-focussed therapeutics" OR "solution-focussed therapeutical" OR
"solution focused therapies" OR "solution focused therapy" OR "solution focused therapeutic" OR "solution focused
therapeutics" OR "solution focused therapeutical" OR "solution focussed therapies" OR "solution focussed therapy" OR
"solution focussed therapeutic" OR "solution focussed therapeutics" OR "solution focussed therapeutical" OR "self-
control therapies" OR "self-control therapy" OR "self-control therapeutics" OR "self-control therapeutical" OR "self-
control therapeutic" OR "self-control training" OR "self-control trainings" OR "self control therapies" OR "self control

therapy" OR "self control therapeutics" OR "self control therapeutical" OR "self control therapeutic" OR "self control
training" OR "self control trainings" OR (("compassion-focused" OR "compassion-focussed" OR "compassion focused"
OR "compassion focussed") AND ("therapies" OR "therapy" OR "therapie" OR "therapist" OR "therapists" OR
"therapeut" OR "treatment" OR "treatments")) OR ("constructivist" AND ("therapies" OR "therapy" OR "therapie" OR
"therapist" OR "therapists" OR "therapeut" OR "treatment" OR "treatments")))
AND
(DE "Depression (Emotion)" "depressive disorder" OR "depression" OR "depressions" OR "depressive" OR DE "Major
Depression" OR "major depression" OR "major depressive disorder" OR DE "Dysthymic Disorder" OR "Dysthymia"
OR " dysthymic disorder" OR DE "Affective Disorders" OR "Affective Disorder" OR "affective disorders" OR "Mood
Disorder" OR "Mood disorders")
Limits: Methodology is ME=(treatment outcome/clinical trial): papers (December 2014)

Cochrane
#1 MeSH descriptor: [Depressive Disorder] explode all trees : 6, 777
#2 "depress*" (Word variations have been searched) : 51, 768
#3 #1 or #2 : 51, 783
#4 "major depressive disorder" (Word variations have been searched) : 5, 435
#5 #3 or #4 : 51, 783
#6 MeSH descriptor: [Dysthymic Disorder] explode all trees : 129
#7 "dysthymi*" (Word variations have been searched) : 649
#8 #6 or #7 : 649
#9 #5 or #8 : 51, 800
#10 "mood disorder" (Word variations have been searched) :4, 034
#11 "affective disorder" (Word variations have been searched) : 2, 882
#12 #10 or #11 : 6, 055
#13 #9 or #12 : 53, 227
#14 MeSH descriptor: [Psychotherapy] explode all trees : 13, 568
#15 "psychotherap*" (Word variations have been searched) : 7, 758
#16 "CBT" (Word variations have been searched) : 2, 029
#17 "Cognitive Behav* therap* (Word variations have been searched) : 8, 893
#18 #14 or #15 or #16 or #17 : 20, 795
#19 'psychodynamic" (Word variations have been searched) : 469
#20 MeSH descriptor: [Psychoanalysis] explode all trees : 13
#21 "psychoanaly*" (Word variations have been searched) : 345
#22 MeSH descriptor: [Counseling] explode all trees : 2, 783
#23 "counseling*" (Word variations have been searched) : 6, 913
#24 "problem solving" (Word variations have been searched) : 2, 867
#25 #18 or #19 or #20 or #21 or #22 or #23 or #24 : 28, 149
#26 "acceptance commitment" (Word variations have been searched) : 168
#27 "assertiveness training" (Word variations have been searched) :231
#28 "behavior activation" (Word variations have been searched) : 663
#29 "mindfulness" (Word variations have been searched) : 466
#30 "metacognitive therap*" (Word variations have been searched) :56
#31 "solution focused therap*" (Word variations have been searched) :858
#32 "self control training" (Word variations have been searched): 5850
#33 #25 or #26 or #27 or #28 or #29 or #30 or #31 or #32 : 32, 748
#34 "Randomized Controlled Trial":ti,ab,kw (Word variations have been searched) : 120, 901
#35 #13 and #33 and #34 in Trials: 4,614
Limit: publication year: 1970-2018

eMethods 2. Detailed list of Which Factors.

Target group
These seven target groups receiving treatment for depression were included: 1) adults (adults in general with no specific demographic characteristic), 2) general medical group, 3) student populations, 4) women suffering from perinatal depression, 5) older adults, 6) other groups, or 7) all these target groups.

Type
We compared interventions for different types of therapies: 1) CBT-based therapies (cognitive behavior therapy, problem-solving therapy, third-wave cognitive therapies, behavioral activation therapy, nondirective supportive therapy, 2) non-CBT-based therapies (psychodynamic therapy, interpersonal therapy, life review therapy, other types of therapy), or 3) all types of therapy.

Format
Interventions could be delivered in 1) group, 2) individual, 3) guided self-help, or 4) other formats (e.g. couples therapy).

Diagnosis
We compared interventions that based their inclusion of participants on different types of diagnoses. These specifications include 1) clinical diagnosis (including mood disorder, chronic depression), or 2) scoring above a clinically relevant cutoff score, 3) subclinical depression or 4) all diagnoses.

Control conditions
We compared interventions for different types of control conditions. These specifications include: 1) care as usual, 2) waiting list, 3) other control conditions, or 4) all types of control conditions.

Risk of bias
We compared studies with different degrees of risk of bias assessed with the risk of bias assessment tool, which assesses possible sources of bias, such as the adequate generation of allocation sequence, the concealment of allocation to conditions, the prevention of knowledge of the allocated intervention, and dealing with incomplete outcome data. We decided to include meta-analyses that were either restrictive in allowing 1) only low risk of bias studies, 2) less restrictive in including all but high risk of bias studies, or 3) meta-analyses including all studies, regardless of bias assessment.

Plessen CY, *et al. BMJ Ment Health* 2023; 26:e300626. doi: 10.1136/bmjment-2022-300626

eMethods 3. Deviations from protocol.

We deviated in four instances from our protocol to ensure the interpretability of our visualizations. In most cases, we reduced *Which* factor categories to broader categories as those categories did not contain enough studies to include in our analyses:
First, we combined psychological interventions into either CBT-based or not-CBT based interventions; Second, we also included couples therapy in the other therapy format category. Third, we did not include two risk of bias assessments (some concern or high risk) as reasonable specifications. Forth, we combined USA and Canada to a new region called North America and included the UK in Europe. **Furthermore, we also included two new *How* factors (3-level and PET-PEESE to account for effect size dependency, included PET-PEESE as an additional method to inspect publication bias, and excluded the unweighted estimate, as this does not seem reasonable to us anymore.**

The text from our protocol is *italic*, the changes we made is **bold**.

Which factors: Which data to analyze?
The decisions of which groups to compare in a meta-analysis are manifold; we decided to specify the following seven relevant study features:

- *Target group. Researchers may decide to compare specific populations only. Included are the categories: 1) adults (adults in general with no specific demographic characteristic), 2) general medical group, 3) perinatal depression, 4) student population, 5) older adults, 6) other groups, or 7) all target groups.*
- *Format. Researchers might compare interventions for different types of therapy delivery: 1) group, 2) individual, 3) guided self-help, 4) couples, 5) telephone, 6) other formats, or 7) all formats.* → **We also included couples therapy in the other therapy format category.**
- *Diagnosis. We compare interventions that based their inclusion of participants on different types of diagnoses. These specifications include the following diagnoses: 1). major depression according to criteria by DSM-V, DSM-IV, DSM-III- R, DSM-III, Research Diagnostic Criteria (RDC) for major depression, or Feighner criteria for depressive disorder. We will also include diagnoses of 2.) mood disorder or other diagnosed disorders (e.g., dysthymia; depression Not Otherwise Specified (NOS); minor depression according to Research Diagnostic Criteria). 3). Chronic depression, i.e., participants meet criteria for chronic or treatment-resistant depression, according to any definition given by the authors of the study. We also include diagnoses defined as 4). scoring above a clinically relevant cutoff score, or 5) diagnosed subclinical depression, i.e. participants score above a cut-off on a self-rating scale, but do not meet criteria for a depressive disorder according to a diagnostic interview (such as the MINI, CIDI or SCID).*
- *Type. Researchers might compare interventions for different types of therapies: 1) cognitive behavior therapy, 2) problem-solving therapy, 3) third-wave cognitive therapies, 4) behavioral activation therapy, 5) nondirective supportive therapy, 6) psychodynamic therapy, 7) interpersonal therapy, 8) life review therapy, 9) other types of therapy, or 10) all types of therapy.* → **We combined theise psychological interventions into either 1) CBT-based or 2) not-CBT based interventions.**
- *Control conditions. Researchers might compare interventions for different types of control conditions. These specifications include: 1) care as usual, 2) waiting list, 3) other types of control conditions, or 4) all types of control conditions.*
- *Country. Researchers might compare studies conducted in different regions: 1) EU, 2) the USA, 3) the UK, 4) Canada, 5) East Asia, 6) Australia, 7) other countries, or 8) all countries.* → **We combined USA and Canada to a new region called North America and included the UK in Europe.**
- *Risk of bias. Researchers might compare studies with different degrees of risk of bias assessed with the risk of bias assessment tool.[416] Based on the assessment of allocation concealment, blinding of assessors, intention-to-treat analyses, and sequence generation, we will create a dichotomous variable indicating whether the study is graded as 1) low risk of bias (if each aspect is rated as low risk) or 2) some concern (if any or all aspects are rated as high risk). We will also include a specification including 3) all studies, regardless of bias assessment.* → **We did not include two risk of bias assessments (some concern or high risk) as reasonable specifications.**
